# Supplementary material for: New Haloterpenes from the Marine Red Alga Laurencia papillosa: Structure Elucidation and Biological Activity
Source: Mar Drugs. 2021 Jan 14;19(1):35. doi: 10.3390/md19010035 (PMC7830550; doi:10.3390/md19010035)
Supplement: Supplementary file 1 [file marinedrugs-19-00035-s001.pdf]

New Haloterpenes from the Marine Red Alga *Laurencia papillosa*: Structure Elucidation and Biological Activity

Mohamed Shaaban[1,2,*], Ghada S. E. Abou-El-Wafa[3], Christopher Golz[2], and Hartmut Laatsch[2,*]

[1] Chemistry of Natural Compounds Department, Division of Pharmaceutical Industries, National Research Centre, El-Behoos St. 33, Dokki-Cairo 12622, Egypt

[2] Institute of Organic and Biomolecular Chemistry, University of Göttingen, Tammannstrasse 2, D-37077 Göttingen, Germany

[3] Department of Botany, Faculty of Science, Mansoura University, Algomhuria st. 60, El-Mansoura 35516, Egypt.

* Correspondence: mshaaba@gmail.com; Tel.: +2-(0)1019644009, +202-2701728-1550; hlaatsc@gwdg.de; Tel.: +49-(0)551-393211

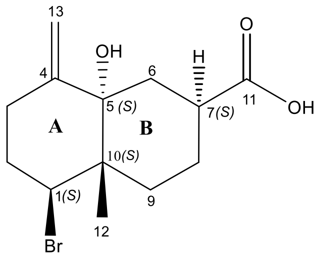

1

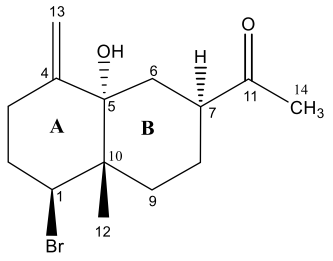

2

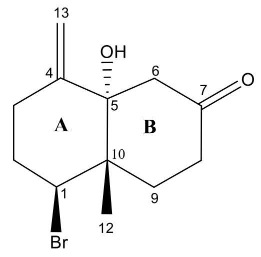

3

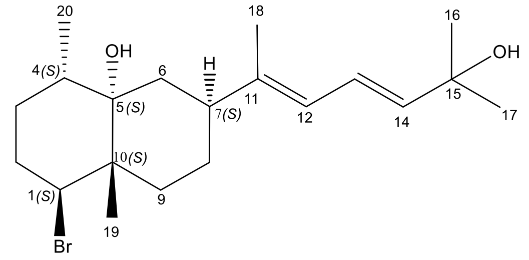

4

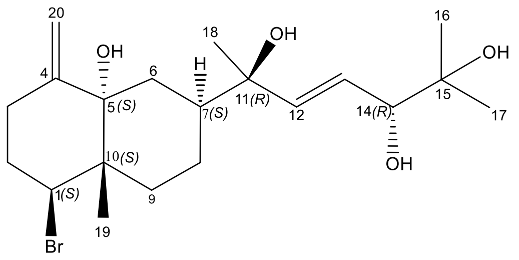

5a

5b: C-11 or C-14 epimer of **5a**

5c: 1 additional OH at unknown pos.

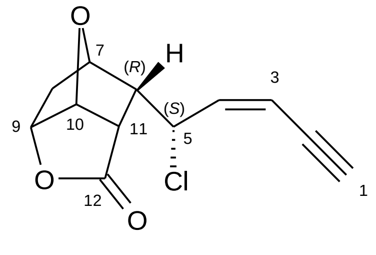

6

Figure S1: Structures of the new haloterpenes (**1-3, 5a-c, 6**) from *Laurencia papillosa*

List of Content

1 The determination of the relative configuration of aplysiolic acid 6

2 The determination of the absolute configuration of aplysiolic acid 8

3 Determination of the absolute configuration of aplysiolic acid from chiroptical data 9

4 7-Acetyl-aplysiol 11

5 X-ray diffraction of 7-acetyl-aplysiol 12

6 Epimeric 11,14-dihydroaplysia-5,11,14,15-tetrols (**5a/b**) 13

7 11,14-Dihydro-11-hydroperoxyaplysia-5,14,15-triol or 11,14-Dihydroaplysia-x,5,11,14,15-pentol........ 14

8 Alternative structures of 5-*epi*-Maneolactone (**6**) 30

9 Crystal structure determination of 5-*epi*-Maneolactone (**6**) 31

Tables

Table S1: DFT-calculated H,H-distances [Å] in the main conformer of all-(*S*)-aplysiolic acid (**1**) (n = 3, Boltzmann factor = 0.815). Experimental NOE contacts are drawn with green arrows in the structures and highlighted in the table; see also the main part Figure 2. 7

Table S2: Experimental ^{13}C NMR data (300 MHz, $CDCl_3$) of aplysiolic acid (1) in comparison with the calculated shifts of eight 1-diastereomers (relative configurations). The calculated shifts are Boltzmann-weighted averages of (n) conformers with Boltzmann factors > 0.01. Yellow columns = *cis*-decalins; blue columns = *trans*-decalins. Pink cells = value out of the confidence limit. 8

Table S3: Experimental and calculated H,H coupling constants (300 MHz, $CDCl_3$) of the eight diastereomers of aplysiolic acid (1). 9

Table S4: Experimental optical rotations and calculated ORD data for the all-(*S*)-configured stereoisomers of aplysiolic acid (**1**), 7-acetylaplysiol (**2**), aplysiadiol (**4**), and anhydroaplysiadiol. 9

Table S5: Calculated ^{13}C shifts for the side chain of the hypothetical isomers of hydroperoxide 5c with (*S*,*S*,*S*,*S*)-configured decalin core. In each of the four diastereomers, one OH group of the trihydroxy-methylheptyl side chain is substituted by a OOH group. Shifts of hydroperoxy carbons are highlighted yellow, of hydroxy carbons blue; especially the methyl groups C-18 at hydroperoxy carbons C-11 are showing a strong upfield shift and are labeled pink. 16

Table S6: Atom distances [in Å] of the 11,14-diastereomers of (1*S*,5*S*,7*S*,10*S*)-aplysiadiol (5a). The molecule geometries were calculated with SPARTAN'18 using ®B97X-D/6-31G*, the Boltzmann factors additionally with wB97X-V/6-311+G(2df,2p). 17

Table S7: Crystal data and structure refinement for **2** and **6** 31

Figures

Figure S1: Structures of the new haloterpenes (**1-3**, **5a-c, 6**) from *Laurencia papillosa* 1

Figure S2: Alignment of the four diastereomers of **1** with (5*R*,10*S*)-*cis*-decalin core (red/blue stereo view); values are averaged distances (in [Å]) calculated for the main conformers; for the rotatable methyl group, the shortest possible H,H distance was used. Green arrow: NOE visible and expected; red arrows: NOE not visible and not expected. 6

Figure S3: Alignment of the four diastereomers of **1** with a (5*S*,10*S*)-*trans*-decalin core (red/blue stereo view); values are averaged distances (in [Å]) calculated for the main conformers; for the rotatable methyl group, the shortest possible H,H distance was used. Green arrows: NOE visible and expected; red arrows: NOE not visible and not expected for the *trans*-decalins. 6

Figure S4: Experimental ECD spectrum of aplysiolic acid (**1**) in methanol (green line) in comparison with the curve calculated for the all-(*R*)-isomer (red line). 11

Figure S5: Experimental ECD spectrum of 7-acetyl-aplysiol (**2**) in methanol in comparison with the curves calculated for the all-(*R*)- and all-(*S*)-enantiomers. 12

Figure S6: Crystal structure of all-(*S*)-7-acetyl-aplysiol (**2**) by X-ray diffraction. 12

Figure S7: Experimental NOESY correlations of 11,14-dihydroaplysia-5,11,14,15-tetrol [(1*S*,5*S*,7*S*,10*S*,11*R*, 14*R*)-configured main isomer **5a**]. In the stereo view on top, observed correlations agreeing with DFT calculations are indicated by red connecting lines. 14

Figure S8: Experimental shifts of 1,1,3-trimethyl-3-(4-methylphenyl)butyl hydroperoxide in $CDCl_3$ (red values) and calculated shifts for vacuum (SPARTAN, black values and MP2, 6-31G(d,p), blue values from Ref.[2]). For comparison see the respective alcohol on the right side, also with calculated shifts. 15

Figure S9: Stereo views of the four (11,14)-diastereomers with (SSSS)-configured decalin core. 21

Figure S10: Stereo views of the four pairs of aligned diastereomers of 11,14-dihydroaplysiatetrols (**5**) with pseudoenantiomeric side chains. 23

Figure S11: According to the ^{1}H NMR double bond signal of H-12 and H-13, **3** is a mixture of three similar compounds, each with *trans*-configured side chains. 24

Figure S12: Preparative HPLC of the MSGL2 (5a-c) mixture on Nucleodur C18; detection by light scattering (figure on the left) and UV absorption (on the right). 25

Figure S13: HPLC/MS of the MSGL2 mixture; detection by MS and SIM (selective ion monitoring) 26

Figure S14: Analytical HPLC/HRMS of dihydroaplysia-5,11,14,15-tetrol (**3**) mixture with a) mass and UV detection and b) SIM mode at 434 and 450 Dalton. 28

Figure S15: Structure of dihydroaplysia-5,11,14,15-tetrol (**5**) with all experimental H,H COSY ( ) and HMBC ( ) correlations, shown for the (1*R*,5*R*,7*R*,10*R*,11*R*,14*S*)-stereoisomer. 29

Figure S16: Alternative structures of 5-*epi*-maneolactone (**6**), calculated with COCON from the experimental COSY and HMBC correlations, using ((*Z*)-pent-2-en-4-ynyl)-cyclohexane as core structure. 30

Figure S17: (-)-ESI mass spectrum of aplysiolic acid (**1**). 33

Figure S18: (-)-ESI HR mass spectrum of aplysiolic acid (**1**). 34

Figure S19: (-)-ESI HR mass spectrum of aplysiolic acid (**1**). 35

Figure S20: ^{1}H NMR spectrum (300 MHz, $CDCl_3$) of aplysiolic acid (**1**) 36

Figure S21: ^{1}H NMR spectrum (600 MHz, $CDCl_3$) of aplysiolic acid (**1**) 37

Figure S22: ^{13}C NMR spectrum (125 MHz, $CDCl_3$) of aplysiolic acid (**1**) 38

Figure S23: H,H COSY (^3J , ^4J) and HMBC ( ) correlations of aplysiolic acid (**1**). Geminal COSY couplings are not depicted. 39

Figure S24: H,H COSY spectrum (500 MHz, $CDCl_3$) of aplysiolic acid (**1**) 40

Figure S25: HMQC spectrum (500 MHz, $CDCl_3$) of aplysiolic acid (**1**) 41

Figure S26: HSQC spectrum (500 MHz, $CDCl_3$) of aplysiolic acid (**1**). 42

Figure S27: HMBC spectrum (500 MHz, $CDCl_3$) of aplysiolic acid (**1**) 43

Figure S28: NOESY spectrum (500 MHz, $CDCl_3$) of aplysiolic acid (**1**). 44

Figure S29: (+)-ESI mass spectrum of 7-acetyl-aplysiol (**2**) 45

Figure S30. (-)-ESI mass spectrum of 7-acetyl-aplysiol (**2**) 46

Figure S31: (-)-ESI mass spectrum of 7-acetyl-aplysiol (**2**) 47

Figure S32: (+)-ESI HR mass spectrum of 7-acetyl-aplysiol (**2**) 48

Figure S33: (+)-ESI HR mass spectrum of 7-acetyl-aplysiol (**2**) 49

Figure S34: (-)-ESI HR mass spectrum of 7-acetyl-aplysiol (**2**) 50

Figure S35: ^{1}H NMR spectrum (600 MHz, $CDCl_3$) of 7-acetyl-aplysiol (**2**) 51

Figure S36: ^{1}H NMR spectrum (300 MHz, $CDCl_3$) of 7-acetyl-aplysiol (**2**) 52

Figure S37: ^{13}C NMR spectrum (125 MHz, $CDCl_3$) of 7-acetyl-aplysiol (**2**) 53

Figure S38: H,H COSY (^{3}J , ^{2}J,^{4}J) and HMBC ( ) correlations of 7-acetyl-aplysiol (**2**) and aplysiol-7-one (**3**)........ 54

Figure S39: H,H COSY spectrum (600 MHz, $CDCl_3$) of 7-acetyl-aplysiol (**2**) 55

Figure S40: H,H COSY spectrum (600 MHz, $CDCl_3$) of 7-acetyl-aplysiol (**2**) 56

Figure S41: HMQC spectrum (600 MHz, $CDCl_3$) of 7-acetyl-aplysiol (**2**) 57

Figure S42: HSQC spectrum (600 MHz, $CDCl_3$) of 7-acetyl-aplysiol (**2**) 58

Figure S43: HMBC spectrum (600 MHz, $CDCl_3$) of 7-acetyl-aplysiol (**2**) 59

Figure S44: HMBC spectrum (600 MHz, $CDCl_3$) of 7-acetyl-aplysiol (**2**) 60

Figure S45: NOESY spectrum (600 MHz, $CDCl_3$) of 7-acetyl-aplysiol (**2**) 61

Figure S46: NOESY spectrum (600 MHz, $CDCl_3$) of 7-acetyl-aplysiol (**2**) 62

Figure S47: ^{1}H NMR spectrum (600 MHz, $CDCl_3$) of aplysiol-7-one (**3**) and 10-hydroxykahukuene B 63

Figure S48: ^{13}C NMR spectrum (125 MHz, $CDCl_3$) of aplysiol-7-one (**3**) and 10-hydroxykahukuene B........ 64

Figure S49: H,H COSY spectrum (600 MHz, $CDCl_3$) of aplysiol-7-one (**3**) and 10-hydroxykahukuene B 65

Figure S50: HMQC spectrum (600 MHz, $CDCl_3$) of aplysiol-7-one (**3**) and 10-hydroxykahukuene B........ 66

Figure S51: HMBC spectrum (600 MHz, $CDCl_3$) of aplysiol-7-one (**3**) and 10-hydroxykahukuene B 67

Figure S52: NOESY spectrum (600 MHz, $CDCl_3$) of aplysiol-7-one (**3**) and 10-hydroxykahukuene B 68

Figure S53: (+)-ESI mass spectrum of dihydroaplysia-5,11,14,15-tetrol (**5**)........ 69

Figure S54: (+)-ESI mass spectrum of dihydroaplysia-5,11,14,15-tetrol (**5**)........ 70

Figure S55: (-)-ESI mass spectrum of dihydroaplysia-5,11,14,15-tetrol (**5**) 71

Figure S56: (+)-ESI HR mass spectrum of dihydroaplysia-5,11,14,15-tetrol (**5**)........ 72

Figure S57: ^{1}H NMR spectrum (300 MHz, CD_3OD) of dihydroaplysia-5,11,14,15-tetrol (**5**) 73

Figure S58: ^{1}H NMR spectrum (600 MHz, CD_3OD) of 10,13-dihydroxy-11-ene-aplysiadiol (**5**) 74

Figure S59: ^{13}C NMR spectrum (125 MHz, CD_3OD) of dihydroaplysia-5,11,14,15-tetrol (**5**) with magnified section of the two signals between 77-81 ppm. 75

Figure S60: Magnified ^{13}C NMR spectrum (125 MHz, CD_3OD) of dihydroaplysia-5,11,14,15-tetrol (**5**) 76

Figure S61: H,H COSY ( ) and HMBC ( ) connectivities of 11,14-dihydroaplysia-5,11,14,15-tetrols (**5**); correlations in the decalin ring were the same as in **1-3**........ 82

Figure S62: H,H COSY spectrum (500 MHz, CD_3OD) of dihydroaplysia-5,11,14,15-tetrol (**5**) 83

Figure S63: HMQC spectrum (500 MHz, CD_3OD) of dihydroaplysia-5,11,14,15-tetrol (**5**) 84

Figure S64: HSQC spectrum (500 MHz, CD_3OD) of dihydroaplysia-5,11,14,15-tetrol (**5**) 85

Figure S65: HMBC spectrum (500 MHz, CD_3OD) of dihydroaplysia-5,11,14,15-tetrol (**5**) 86

Figure S66: NOESY spectrum (500 MHz, CD_3OD) of dihydroaplysia-5,11,14,15-tetrol (**5**)........ 87

Figure S67: (+)-ESI mass spectrum of 5-*epi*-maneolactone (**6**) 88

Figure S68: (+)-ESI HR mass spectrum of 5-*epi*-maneolactone (**6**) 89

Figure S69: (+)-ESI HR mass spectrum of 5-*epi*-maneolactone (**6**) 90

Figure S70: ^{1}H NMR spectrum (300 MHz, $CDCl_3$) of 5-*epi*-maneolactone (**6**) 91

Figure S71: ^{13}C NMR spectrum (125 MHz, $CDCl_3$) of 5-*epi*-maneolactone (**6**)........ 92

Figure S72: APT NMR spectrum (125 MHz, $CDCl_3$) of 5-*epi*-maneolactone (**6**)........ 93

Figure S73: H,H COSY ( ) and selected HMBC ( ) correlations of 5-*epi*-maneolactone (**6**)........ 94

Figure S74: H,H COSY spectrum (500 MHz, $CDCl_3$) of 5-*epi*-maneolactone (**6**) 95

Figure S75: HMQC spectrum (500 MHz, $CDCl_3$) of 5-*epi*-maneolactone (**6**) 96

Figure S76: HSQC spectrum (500 MHz, $CDCl_3$) of 5-*epi*-maneolactone (**6**) 97

Figure S77: HMBC spectrum (500 MHz, $CDCl_3$) of 5-*epi*-maneolactone (**6**) 98

Figure S78: NOESY spectrum (500 MHz, $CDCl_3$) of 5-*epi*-maneolactone (**6**) 99

1 The determination of the relative configuration of aplysiolic acid

The NOESY spectrum of aplysiolic acid (**1**) showed strong correlations between (*Z*)-H13 (δ 4.81) and *both* H_2-6 protons (δ_{eq} 1.83, δ_{ax} 2.04) and an NOE signal between Me-12 (δ 0.96) and the CH_{ax}-6 proton at δ 2.04). With respect to *ab initio* calculated atom distances in the molecule, both correlations should not be visible in the *cis*-isomer (Figure S2), but are definitely requiring a *trans*-configuration of the decalin ring system (see Figure S3 and Table S1).

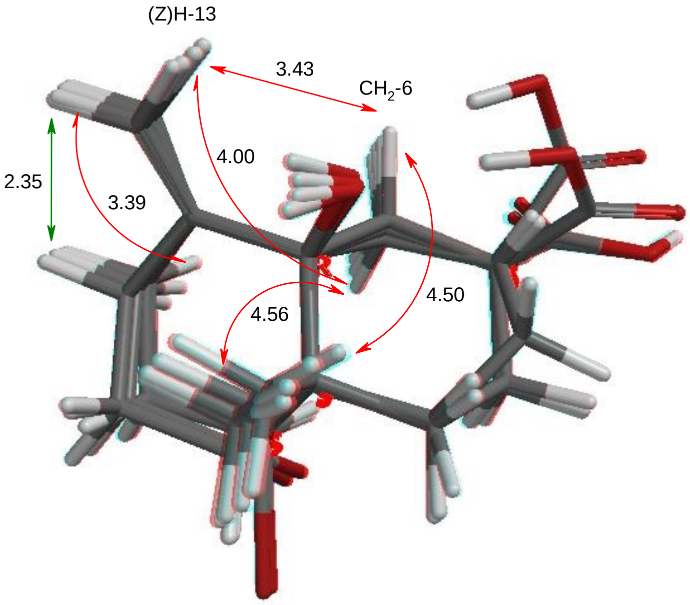

Figure S2: Alignment of the four diastereomers of **1** with (5*R*,10*S*)-*cis*-decalin core (red/blue stereo view); values are averaged distances (in [Å]) calculated for the main conformers; for the rotatable methyl group, the shortest possible H,H distance was used. Green arrow: NOE visible and expected; red arrows: NOE not visible and not expected.

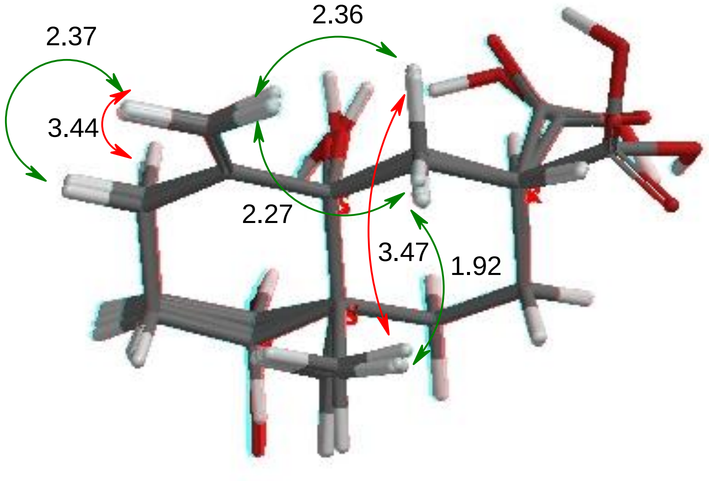

Figure S3: Alignment of the four diastereomers of **1** with a (5*S*,10*S*)-*trans*-decalin core (red/blue stereo view); values are averaged distances (in [Å]) calculated for the main conformers; for the rotatable methyl group, the shortest possible H,H distance was used. Green arrows: NOE visible and expected; red arrows: NOE not visible and not expected for the *trans*-decalins.

Table S1: DFT-calculated H,H-distances [Å] in the main conformer of all-(*S*)-aplysiolic acid (**1**) (n = 3, Boltzmann factor = 0.815). Experimental NOE contacts are drawn with green arrows in the structures and highlighted in the table; see also the main part Figure 2.

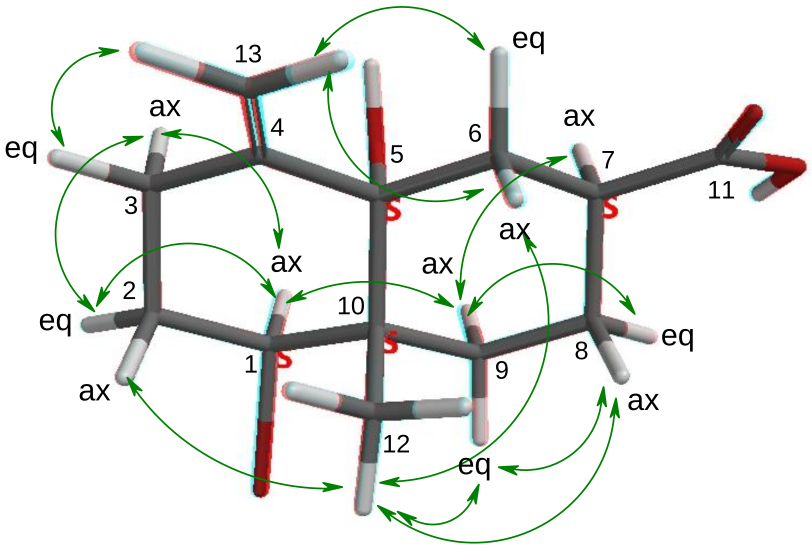

H, H	Shift [δ]	distance [Å]	H, H	Shift [δ]	distance [Å]
$1_{\alpha ax}$, $2_{ßax}$	4.72, 2.20	3.06	$6_{ßax}$, $8_{ßeq}$	2.04, 1.90	3.82
$1_{\alpha ax}$, $2_{\alpha eq}$	4.72, 2.13	2.46	6_{aeq}, $8_{\beta ax}$	1.83, 1.66	3.81
$1_{\alpha ax}$, $3_{ßeq}$	4.72, 2.70	3.81	6_{aeq}, 8_{aeq}	1.83, 1.90	4.31
$1_{\alpha ax}$, $3_{\alpha ax}$	4.72, 2.15	2.65	$6_{\beta ax}$, ßMe12	2.04, 0.96	1.95
$1_{\alpha ax}$, $9_{ßeq}$	4.72, 1.77	3.12	6_{aeq}, ßMe12	1.83, 0.96	3.48
$1_{\alpha ax}$, $9_{\alpha ax}$	4.72, 1.77	2.37	$6_{\beta ax}$, 13(*Z*)	2.04, 4.81	2.31
$1_{\alpha ax}$, ßMe12	4.72, 0.96	3.58	6_{aeq}, 13(*Z*)	1.83, 4.81	2.33
$2_{ßax}$, $3_{ßeq}$	2.20, 2.15 not separated	2.46	7_{aax}, $9_{\beta eq}$ overlapping with next entry	2.94, 1.77	3.79
$2_{ßax}$, $3_{\alpha ax}$	2.20, 2.70	3.06	7_{aax}, 9_{aax}	2.94, 1.77	2.63
$2_{\alpha eq}$, $3_{ßeq}$	2.13, 2.15 not separated	2.54	$8_{\beta ax}$, $9_{\beta eq}$	1.66, 1.77	2.46
$2_{\alpha eq}$, $3_{\alpha ax}$	2.13, 2.70	2.43	$8_{\beta ax}$, 9_{aax}	1.66, 1.77	3.07
$2_{ßax}$, ßMe12	2.20, 0.96	1.94	8_{aeq}, $9_{\beta eq}$	1.90, 1.77	2.48
$2_{\alpha eq}$, ßMe12	2.13, 0.96	3.50	8_{aeq}, 9_{aax}	1.90, 1.77	2.44
$3_{ßeq}$, 13(*E*)	2.15, 4.90	2.37	$8_{\beta ax}$, ßMe12	1.66, 0.96	2.04
3_{aax}, 13(*E*)	2.70, 4.90	3.41	8_{aeq}, ßMe12	1.90, 0.96	3.55
$6_{ßax}$, 7_{aax}	2.04, 2.94	3.05	$9_{\beta eq}$, ßMe12	1.77, 0.96	2.31
6_{aeq}, 7_{aax}	1.83, 2.94	2.46	9_{aax}, ßMe12	1.77, 0.96	3.55
$6_{ßax}$, $8_{ßax}$	2.04, 1.66 weak?	2.67	ßMe12, 13(*Z*)	0.96, 4.81 (weak)	2.93

2 The determination of the absolute configuration of aplysiolic acid

The relative configuration of aplysiolic acid (**1**) was derived as *rel*-(*SSSS*) from NOE interactions (see main part). Trials to confirm this result additionally by calculating the ^{13}C NMR shifts were weak, as the correlation coefficients and the sum of deviations between experimental and calculated ^{13}C shifts of the *rel*-(2*R*,4a*S*,5*S*,8a*R*), (2*R*,4a*R*,5*R*,8a*R*), and (2*S*,4a*R*,5*R*,8a*R*)-diastereomers were too similar (Table S2).

Table S2: Experimental ^{13}C NMR data (300 MHz, $CDCl_3$) of aplysiolic acid (**1**) in comparison with the calculated shifts of eight **1**-diastereomers (relative configurations). The calculated shifts are Boltzmann-weighted averages of (n) conformers with Boltzmann factors > 0.01. Yellow columns = *cis*-decalins; blue columns = *trans*-decalins. Pink cells = value out of the confidence limit.

atom no.	δ_C (exp) **1**	1*R*,5*R*,7*R*, 10*S*-**1** (n = 6)	1*R*,5*R*,7*S*, 10*S*-**1** (n = 10)	1*S*,5*R*,7*S*, *10S*-**1** (n = 3)	1*S*,5*R*,7*R*, 10*S*-**1** (n = 6)	1*R*,5*R*,7*R*, 10*R*-**1** (n = 3)	1*R*,5*R*,7*S*, 10*R*-**1** (n = 3)	1*S*,5*R*,7*S*, 10*R*-**1** (n = 1)	1*S*,5*R*,7*R*, 10*R*-**1** (n = 3)
1	62,9	61,4	63,2	53,0	54,5	58,4	59,1	65,3	65,9
2	33,9	35,1	32,8	33,5	34,0	34,2	34,2	33,3	33,6
3	32,5	28,8	29,0	31,9	31,7	32,1	32,1	27,3	27,0
4	148,5	152,9	150,5	148,4	150,7	149,1	150,1	147,2	148,5
5	76,2	76,7	77,1	79,3	78,4	78,5	76,9	78,8	76,8
6	34,1	37,0	33,4	31,4	35,1	35,2	29,9	29,8	34,5
7	38,2	35,9	40,1	41,1	37,1	39,7	39,5	40,3	39,6
8	23,5	24,6	22,9	22,9	23,4	24,8	23,3	24,3	23,8
9	32,0	35,3	32,6	29,4	31,9	31,2	28,5	30,0	31,2
10	43,0	43,8	43,5	44,8	44,8	44,6	45,0	42,6	43,0
11	181,0	175,9	174,5	172,8	176,1	172,6	175,3	173,1	173,3
12	14,8	27,2	25,4	16,8	17,0	14,5	13,7	22,2	22,5
13	110,2	110,3	113,0	113,9	111,1	113,3	110,6	111,9	111,6
correl. coeff.		0,99670	0,99740	0,99689	0,99827	0,99851	0,99885	0,99741	0,99788
$\Sigma \lvert \delta_{exp}-\delta_{calc} \rvert$		39,3	32,0	38,6	25,8	26,2	25,2	38,7	29,7

Analysis on basis of the individual shifts was more convincing. With exception of carbonyl values, ^{13}C shifts predicted by DFT calculations are very reliable, and deviations from the experimental value of more than $\Delta\delta \pm 5$ ppm are suspicious, and differences of $\Delta\delta > \pm 10$ ppm are usually indicating wrong structures. It follows that of the *trans*-isomers, only the *rel*-(1*R*,5*R*,7*R*,10*R*)- and perhaps the *rel*-(1*R*,5*R*,7*S*,10*R*)-(**1**) diastereomers should be taken into consideration: Amongst these, only in *rel*-(1*R*,5*R*,7*R*,10*R*)-**1**, H_{ax}-6 is in a diaxial position with H-7 and should show a large coupling constant with the latter one, as found experimentally (Table S3); this confirmed the NOE-derived relative configuration.

Table S3: Experimental and calculated H,H coupling constants (300 MHz, $CDCl_3$) of the eight diastereomers of aplysiolic acid (**1**)

J_{exp} [Hz]	1*R*,5*R*,7*R*, 10*S*-**1** $J_{x,y}$	1*R*,5*R*,7*S*, 10*S*-**1** $J_{x,y}$	1*S*,5*R*,7*S*, 10*S*-**1** $J_{x,y}$	1*S*,5*R*,7*R*, 10*S*-**1** $J_{x,y}$	1*R*,5*R*,7*R*, 10*R*-**1** $J_{x,y}$	1*R*,5*R*,7*S*, 10*R*-**1** $J_{x,y}$	1*S*,5*R*,7*S*, 10*R*-**1** $J_{x,y}$	1*S*,5*R*,7*R*, 10*R*-**1** $J_{x,y}$
x/z	6α/6β<br>$6\alpha_{ax}/7\beta_{ax}$<br>$6\beta_{eq}/7\beta_{ax}$	6α/6β<br>$6\alpha_{ax}/7\alpha_{eq}$<br>$6\beta_{eq}/7\alpha_{eq}$	6α/6β<br>$6\alpha_{ax}/7\alpha_{eq}$<br>$6\beta_{eq}/7\alpha_{eq}$	6α/6β<br>$6\alpha_{ax}/7\beta_{ax}$<br>$6\beta_{eq}/7\beta_{ax}$	6α/6β<br>$6\alpha_{ax}/7\beta_{ax}$<br>$6\beta_{eq}/7\beta_{ax}$	6α/6β<br>$6\alpha_{ax}/7\alpha_{eq}$<br>$6\beta_{eq}/7\alpha_{eq}$	6α/6β<br>$6\alpha_{ax}/7\alpha_{eq}$<br>$6\beta_{eq}/7\alpha_{eq}$	6α/6β<br>$6\alpha_{ax}/7\beta_{ax}$<br>$6\beta_{eq}/7\beta_{ax}$
12.8*)	19.9	-2.6	-15.9	6.8	-26.2	-21.2	-17.8	-40.4
13.8*)	13.5	8.5	7.3	13.3	13.4	6.3	7.4	13.4
mult.	3.0	0.97	0.8	3.7	3.1	1.2	0.7	3.6

*) values may be exchanged

3 Determination of the absolute configuration of aplysiolic acid from chiroptical data

From a chiral compound with known relative configuration, the absolute chirality can be determined usually straightforward by comparing the calculated ECD spectra and/or the optical rotation dispersion (ORD) with the experimental values.

As previously derived from NOE, ^{13}C and ^{1}H NMR spectra, aplysiolic acid (**1**) is having the all-(*R*)- or all-(*S*)-configuration. From the positive sign calculated for the all-(*R*)-enantiomer in comparison with the negative experimental OR it follows, that the natural product should have the all-(*S*)-configuration.

Table S4: Experimental optical rotations and calculated ORD data for the all-(*S*)-configured stereoisomers of aplysiolic acid (**1**), 7-acetylaplysiol (**2**), aplysiadiol (**4**), and anhydroaplysiadiol.

name	confor-mer	Boltzmann factor *)	589 nm	weighted OR at 589 nm	578 nm	546 nm	436 nm	365 nm
Aplysiolic acid (**1**)	1	0.798	-21.62	-17.25	-22.71	-26.40	-50.42	-91.30
	2	0.166	-112.80	-18.72	-117.51	-133.08	-220.58	-335.56
	3	0.036	-96.55	-3.48	-100.73	-114.63	-195.61	-310.71
weighted average $[\alpha]_D$			Σ	**-39.45**				
opt. rotation $[\alpha]_D$			**exp**	**-34.8**				
7-Acetylaplysiol (**2**)	01	0.401549	-78.46	-31.50	-82.30	-95.40	-182.35	-351.00
	02	0.345118	-30.46	-10.51	-32.23	-38.39	-85.06	-196.74
	03	0.115940	-101.92	-11.82	-105.77	-118.17	-176.07	-196.01
	04	0.066986	-88.47	-5.93	-91.62	-101.63	-142.99	-128.91
	05	0.027813	-105.32	-2.93	-110.11	-126.22	-226.28	-397.65
	06	0.026744	-106.28	-2.84	-111.12	-127.38	-228.42	-401.64
	07	0.015849	-55.26	-0.88	-57.55	-65.07	-105.79	-148.71

weighted average [α]D			Σ	**-66.41**				
opt. rotation [α]D			**exp.**	**-55.4**				
Aplysiadiol (**4**)	01	0.168043	-39.60	-6.65	-41.46	-47.70	-86.63	-149.63
	02	0.143058	-132.12	-18.90	-138.15	-158.42	-282.74	-480.09
	03	0.075779	-193.54	-14.67	-202.18	-231.09	-404.84	-669.85
	04	0.069697	-103.38	-7.21	-107.95	-123.23	-214.07	-349.02
	05	0.065337	-86.25	-5.64	-90.28	-103.84	-188.47	-326.72
	06	0.063227	-86.78	-5.49	-90.73	-103.97	-184.81	-311.81
	07	0.058958	164.06	9.67	171.53	196.59	350.38	595.95
	08	0.058771	65.04	3.82	68.07	78.28	142.28	248.75
	09	0.046360	-93.07	-4.31	-97.36	-111.79	-200.93	-343.84
	10	0.045196	-84.23	-3.81	-87.70	-99.14	-162.31	-241.00
	11	0.028392	-147.57	-4.19	-154.19	-176.36	-310.13	-515.75
	12	0.028122	-170.70	-4.80	-178.30	-203.69	-355.84	-586.37
	13	0.025647	129.24	3.31	135.09	154.70	274.41	463.90
	14	0.023613	94.56	2.23	98.92	113.61	204.96	355.03
	15	0.022019	-141.24	-3.11	-147.51	-168.46	-293.55	-481.61
	16	0.017741	-182.75	-3.24	-190.87	-218.00	-380.21	-624.56
	17	0.017703	107.43	1.90	112.32	128.74	229.54	390.97
	18	0.007468	-118.58	-0.89	-124.00	-142.21	-254.00	-431.39
	19	0.007429	-32.15	-0.24	-33.66	-38.72	-70.32	-121.69
	20	0.007413	-117.87	-0.87	-123.21	-141.15	-250.55	-422.67
	21	0.007227	-95.34	-0.69	-99.74	-114.56	-206.11	-353.04
	22	0.006081	-63.15	-0.38	-66.01	-75.61	-133.98	-224.81
	23	0.003491	-273.79	-0.96	-287.02	-331.86	-619.85	-1115.13
	24	0.003228	-370.23	-1.20	-387.81	-447.23	-823.37	-1453.70
weighted average [α]D			Σ	**-66.29**				
opt. rotation [α]D			**exp.**	**-60.7**				
Anhydroaplysiadiol	01	0.381076	-104.13	-39.68	-109.16	-126.25	-237.77	-442.63
	02	0.263605	69.66	18.36	72.97	84.21	156.99	289.96
	03	0.177018	-146.84	-25.99	-153.12	-173.97	-293.40	-455.08
	04	0.161437	162.26	26.19	169.85	195.49	358.62	645.94
	05	0.016864	-92.99	-1.57	-97.55	-113.04	-215.48	-408.66
weighted average [α]D			Σ	**-22.69**				
exp. rotation [α]D			**exp.**	**-116.2**				

) calculated with ωB97X-V/6-311+G(2df,2p)[6-311G]

For a further confirmation, also the ECD data were measured and compared with calculated spectra. Surprisingly, the opposite absolute all-(*R*)-configuration resulted with this method (Figure S4)!

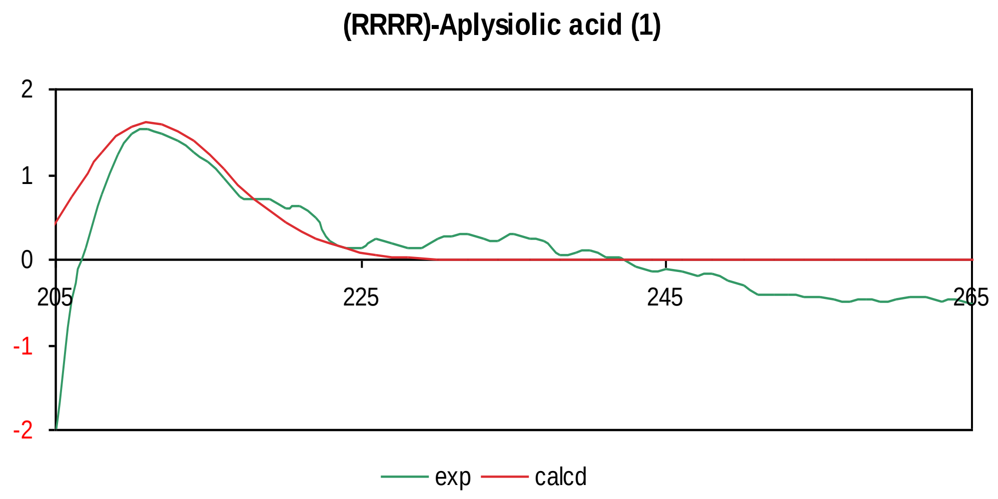

Figure S4: Experimental ECD spectrum of aplysiolic acid (**1**) in methanol (green line) in comparison with the curve calculated for the all-(*R*)-isomer (red line).

Although both chiroptical parameters are correlated *via* the Kramers-Kronig equation and are reflecting identical facts just from different points of view, ECD and ORD calculations may yield contradictory results and thereby opposite absolute configurations. With respect to ECD, this may happen if the chromophor is not close enough to the chirality center(s) or the absorption is weak or not in the observable wavelength range; both may be the case for **1**. On the other side, if optical rotations with different signs are predicted for the individual conformers, the Boltzmann-weighted optical rotation may be a small difference between large values, having a low accuracy. In this case, the sign of the OR will strongly depend on the accuracy of the calculation; also solvent effects and hydrogen bridging may influence the conformer equilibrium and thereby the result.

As the calculated rotations of **1** and **2** are having the same sign for all conformers and all calculated wavelengths (Table S4), their ORD-derived absolute configurations are reliable.[1] However, we could not exclude the same contradiction for aplysiadiol (**4**) and anhydroaplysiadiol, as for these compounds ECD spectra were not available.

4 7-Acetyl-aplysiol

NOE correlations confirmed the same relative configuration for 7-acetylaplysiol (**2**) as for **1**. The ORD-calculation predicted for the all-(*S*)-isomer of **2** a negative OR value, which agreed with the negative experimental value, as for **1** (Table S4). However, the experimental ECD spectrum required also here the enantiomeric all-(*R*)-isomer (Figure S5).

---

[1] Mándi, A.; Kurtán, T.; Applications of OR/ECD/VCD to the structure elucidation of natural products. *Nat. Prod. Rep.* **2019**, *36*, 889–918.

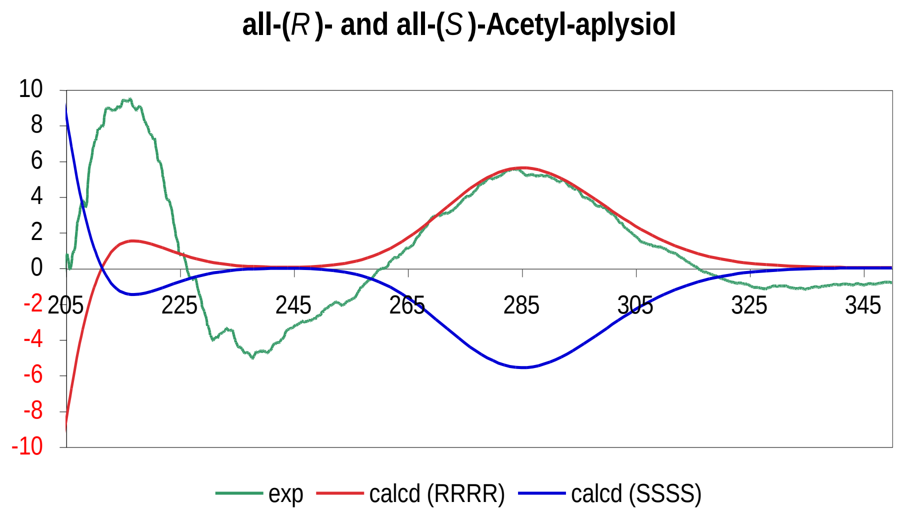

Figure S5: Experimental ECD spectrum of 7-acetyl-aplysiol (**2**) in methanol in comparison with the curves calculated for the all-(*R*)- and all-(*S*)-enantiomers.

5 X-ray diffraction of 7-acetyl-aplysiol

As for all four compounds in Table S4, ORD values were negative (indicating the all-(*S*)-configuration), but ECD calculations gave a peak at 285 nm, pointing to the opposite all-(*R*)-configuration of 7-acetyl-aplysiol (**2**), the latter was additionally analyzed by x-ray diffraction. The result confirmed clearly the NOE-derived relative configuration and also the ORD-derived absolute all-(*S*)-configuration of **2** (Figure S6, Table S7). For biosynthetic reasons and in agreement with the ORD data we are assuming therefore, that the related derivatives **1**, **3**, **4**, and **5a-c** are having the same absolute configuration of the decalin core as well. A reason for the discrepancy between ORD and ECD-derived configurations was not yet found.

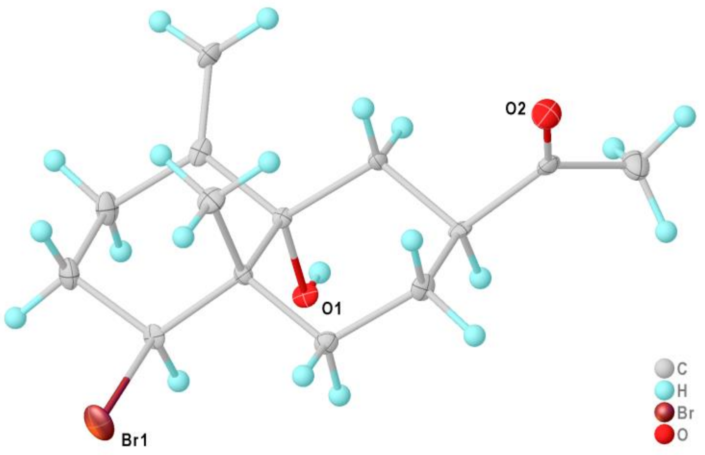

Figure S6: Crystal structure of all-(*S*)-7-acetyl-aplysiol (**2**) by X-ray diffraction.

6 Epimeric 11,14-dihydroaplysia-5,11,14,15-tetrols (5a/b)

The planar structure of compound **5**[*)] was determined by analysis of the 1D and 2D NMR spectra (see main part). A close inspection of the ^{13}C NMR spectrum (Figure S59 ff) of **5** showed, that all signals were groups of mostly each four individual lines with shift differences of < 0.1 ppm, pointing to a mixture of four stereoisomers or otherwise closely related compounds. This was confirmed by the ^{1}H NMR signal of the Δ^{12} *trans*-double bond at δ 5.77 (Figure S11), and by analytical HPLC-MS. The latter method indicated at least two isomers $C_{20}H_{33}BrO_4$ and additionally at least one isomer of compound $C_{20}H_{33}BrO_5$; a preparative separation (Figure S12-Figure S14) failed, however, so that the structures of these compounds had to be analyzed from the mixture.

NOE spectra of mixture **5** displayed a long-range interaction of the terminal isopropyl unit with the exomethylene protons at C-20. According to extensive DFT calculations, hydrogen bridges between C^{5}-OH and 15-OH and between 15-OH and 14-OH are forcing the side chain of all four (11,14)-diastereomers into scorpion-shaped conformations (Figure S9). This was not predicted for the (12*Z*)-isomer (which was excluded by the H,H-coupling constant of the double bond anyway), and was also not expected for the (7*R*)-epimer of **5** (because of the X-ray analysis of **2**).

It followed from the calculations that only for the two (14*R*)-diastereomers the distances between the isopropyl groups Me-16/17 and (*Z*)H-20 were short enough (2.42-2.55 Å; analyzed were the main conformers) to explain the observed NOE effect (see Figure S7 - Figure S10). The intensity of the correlation between Me-18 and both H-12 (weak, 3.61 Å) and H-13 (stronger, 2.01 Å) fitted on the (11*S*,14*S*) and (11*R*,14*R*) isomer. Only the latter one agrees with the (14*R*)-configuration derived above, resulting in the *abs*-(1*S*,5*S*,7*S*,10*S*,11*R*,14*R*)-configuration for the main isomer **5a**. This was confirmed by strong correlations between H-7/H-12 (but not between H-7/H-13) and between CH_{ax}-6/Me-18. In a similar way, observed NOEs from Me-18 with H_{α}-6 (2.39 Å), with H_{β}-6 (2.91 Å), or 13-H (2.36 Å), and between H-7 and H-12 (2.72 Å) respectively, are requiring the (11*R*)-isomers and should not occur in the (11*S*)-configuration (3.62, 3.64 Å). The configuration of the other isomers remained speculative.

[*)] **5** = mixture of **5a**-**5c**

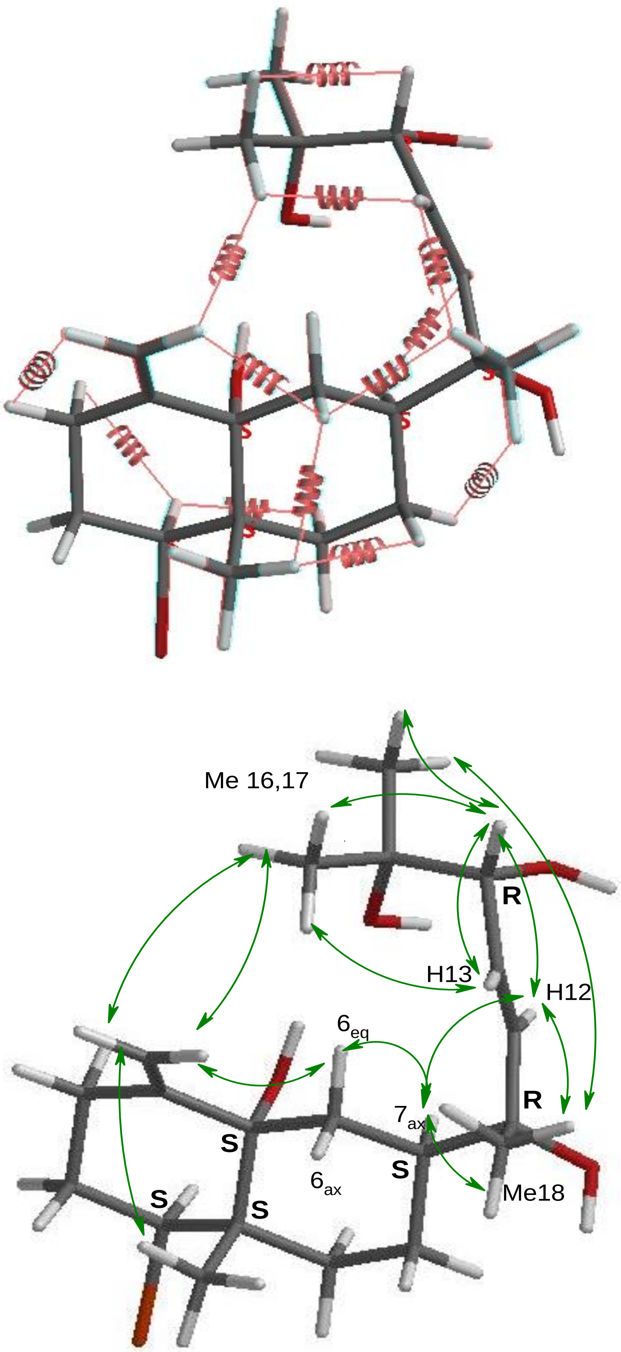

Figure S7: Experimental NOESY correlations of 11,14-dihydroaplysia-5,11,14,15-tetrol [(1*S*,5*S*,7*S*,10*S*,11*R*,14*R*)-configured main isomer **5a**]. In the stereo view on top, observed correlations agreeing with DFT calculations are indicated by red connecting lines.

7 11,14-Dihydro-11-hydroperoxyaplysia-5,14,15-triol or 11,14-Dihydroaplysia-x,5,11,14,15-pentol

According to HPLC/MS data and the discussion above, compound **5** consisted of at least two isomers **5a**/**5b** with the empirical formula $C_{20}H_{33}BrO_4$ and at least one homologue $C_{20}H_{33}BrO_5$ (Figure S13). The ^{13}C NMR spectrum of the mixture showed 20 broadened signals, which appeared on magnification as groups of up to four signals with shift differences of

Δδ < 1 ppm. As $C_{20}H_{33}BrO_4$ and $C_{20}H_{33}BrO_5$ are having the same number of double bond equivalents, the additional oxygen atom cannot form a carbonyl group, an epoxide or another cyclic ether. As no additional low-field carbon signals were found with respect to **5a**, also a further OH group or linear ether was less plausible.

It seemed that the only remaining option was a hydroperoxide group (R-OOH) instead of one of the four OH groups in **5a**. However, we did not see the expected low-field shift of the hydroperoxide carbon of usually >10 ppm, compared with the respective alcohol. Also the strong up-field shift of methyl groups (e.g. of Me-16/17, 18) at the hydroperoxy carbon was missing (see Table S5).

To test the accuracy of our NMR predictions, *ab initio* calculated shifts of 1,1,3-trimethyl-3-(4-methylphenyl)butyl hydroperoxide were compared with the experimental shifts and previously calculated values[2]. It came out that the SPARTAN predictions were closer to the experimental shifts than previous calculations and close to the error range of measurements (Figure S8). Therefore, also the predictions for the hydroperoxides of **5a** were assumed to be reliable.

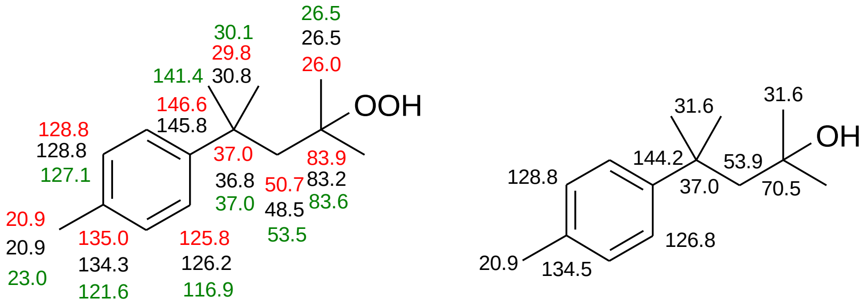

Figure S8: Experimental shifts of 1,1,3-trimethyl-3-(4-methylphenyl)butyl hydroperoxide in $CDCl_3$ (red values) and calculated shifts for vacuum (SPARTAN, black values and MP2, 6-31G(d,p), blue values from Ref.[2]). For comparison see the respective alcohol on the right side, also with calculated shifts.

As no hints for hydroperoxides were seen in the ^{13}C NMR spectrum of **5**, and as also the HPLC/MS spectrum did not change after treating the mixture **5** with $NaBH_4$ or sodium iodide, the peroxide hypothesis was abandoned. The only remaining explanation is therefore a fifth hydroxy group attached to a carbon, whose NMR signal is overlapping with one of the other low-field signals and therefore not distinguishable. As the remaining amount of **5** was insufficient for further separation trials, we must postpone the elucidation of $C_{20}H_{33}BrO_5$ to a later occasion.

[2] Turovskiy, N. A.; Raksha, E. V.; Berestneva, Y. C. NMR ^{13}C Spectra of the 1,1,3-trimethyl-3-(4-methylphenyl)butyl hydroperoxide in various solvents: molecular modelling. *Science J. Volgograd State Univ. Technol. Innov.* 2015, 18, 65–72; (DOI: http:// dx.doi.org/10.15688/jvolsu10.2015.3.7.

Table S5: Calculated ^{13}C shifts for the side chain of the hypothetical isomers of hydroperoxide **5c** with (*S*,*S*,*S*,*S*)-configured decalin core. In each of the four diastereomers, one OH group of the trihydroxy-methylheptyl side chain is substituted by a OOH group. Shifts of hydroperoxy carbons are highlighted yellow, of hydroxy carbons blue; especially the methyl groups C-18 at hydroperoxy carbons C-11 are showing a strong upfield shift and are labeled pink.

		**(11*R*, 14*R*)**				**(11*R*, 14*S*)**				**(11*S*, 14*R*)**				**(11*S*, 14*S*)**			
**at.-no**	**experi-mental**	**triol*)**	**11-OOH**	**14-OOH**	**15-OOH**	**triol*)**	**11-OOH**	**14-OOH**	**15-OOH**	**triol*)**	**11-OOH**	**14-OOH**	**15-OOH**	**triol*)**	**11-OOH**	**14-OOH**	**15-OOH**
7	43.5	42.1	38.7	42.9	44.3	46.8	46.1	46.1	43.2	40.3	40.8	41.6	43.1	49.7	41.6	45.5	39.9
11	75.3	75.1	88.2	75.0	74.7	74.7	86.9	74.6	75.4	74.8	86.6	75.3	75.2	75.9	87.1	75.6	74.9
12	139.8	140.6	137.1	143.1	141.9	139.3	136.3	140.4	136.7	140.0	136.6	144.3	141.8	144.5	138.9	146.5	143.8
13	128.1	128.8	129.0	125.2	130.2	129.5	131.4	126.1	130.1	127.6	130.3	123.5	127.3	126.8	129.6	123.2	127.0
14	80.3	79.0	76.9	92.2	77.4	76.6	76.2	90.7	76.8	76.4	76.8	91.1	76.5	76.8	76.4	92.3	78.9
15	73.7	73.6	74.5	74.3	86.4	74.2	74.8	74.4	86.0	74.8	74.5	75.2	86.3	75.0	74.3	74.1	87.1
16	25.1	25.4	25.5	25.9	19.5	25.0	24.9	24.6	21.2	25.5	25.3	26.0	20.7	24.7	24.6	25.0	18.1
17	26.0	26.8	26.8	26.7	22.4	27.2	27.2	26.7	22.2	27.2	26.6	26.5	22.0	27.2	26.7	27.0	22.8
18	26.3	24.4	16.6	25.6	25.1	25.8	17.7	26.1	27.4	26.9	18.4	27.2	26.4	25.5	19.8	26.4	26.3

*) Calculated side chain shifts of the 11,14,15-triol stereoisomers for comparison.

Table S6: Atom distances [in Å] of the 11,14-diastereomers of (1*S*,5*S*,7*S*,10*S*)-aplysiadiol (**5a**). The molecule geometries were calculated with SPARTAN'18 using ωB97X-D/6-31G*, the Boltzmann factors additionally with wB97X-V/6-311+G(2df,2p).

Calculated diastereomers

11S,14S SSSS

conformer no.	below: Boltzmann factors*)		Me₂15/ Me19	Me₂15/ (*Z*)H20	H6ax/ Me₂15	H6eq/ Me₂15	H6ax/ H12	H6ax/ H13	H6eq/ H12	H6eq/ H13	H6ax/ Me18	H6eq/ Me18	H7/ Me₂15	H7/H12	H7/H13	H12/ Me18	H13/ Me18
		¹H Shifts	1,15 0,89	1,15 4,74	1,75 1,15	1,66 1,15	1,75 5,79	1,75 5,74	1,66 5,79	1,66 5,74	1,75 1,26	1,66 1,26	1,95 1,15	1,95 5,79	1,95 5,74	5,79 1,26	5,74 1,26
1	0,5562	0,4631	>6	5,641	5,064	3,726	3,259	4,873	2,287	3,735	4,413	4,437	2,582	3,360	2,523	3,452	2,091
2	0,2343	0,2953	>6	5,647	5,060	3,726	3,237	4,921	2,248	3,790	4,410	4,442	2,558	3,324	2,580	3,483	2,071
3	0,1298	0,1283	>6	5,599	5,066	3,742	3,404	4,880	2,361	3,733	4,399	4,453	2,586	3,367	2,446	3,413	2,142
4	0,0107	0,0342	>5	2,418	3,457	2,193	4,302	3,242	3,266	2,732	4,297	4,502	3,118	2,592	4,117	2,264	4,017
5	0,0138	0,0293	>5	2,424	3,397	2,253	4,271	3,233	3,239	2,809	4,313	4,487	3,177	2,615	4,165	2,258	3,981
6	0,0165	0,0239	>5	2,420	3,421	2,233	4,278	3,256	3,242	2,806	4,309	4,491	3,162	2,603	4,163	2,261	3,982
7	0,0215	0,0131	>7	5,641	5,211	3,847	3,469	4,679	2,531	3,491	4,417	4,432	2,793	3,500	2,278	3,301	2,279
8	0,0172	0,0129	>6	5,701	5,225	3,862	3,412	4,735	2,458	3,551	4,419	4,426	2,783	3,460	2,346	3,356	2,236
expected NOE for 11S,14S																	
observed experimental NOE				visible		visible			weak	weak	???			visible	weak	visible	visible

) left column: calculation with ωB97XD/6-31G,
right column calculated with ωB97X-V/6-311+G(2df,2p)

11R,14S SSSS

		Distances	Me₂15/ Me19	Me₂15/ (*Z*)H20	H6ax/ Me₂15	H6eq/ Me₂15	H6ax/ H12	H6ax/ H13	H6eq/ H12	H6eq/ H13	H6ax/ Me18	H6eq/ Me18	H7/ Me₂15	H7/H12	H7/H13	H12/ Me18	H13/ Me18
		¹H Shifts	1,15 0,89	1,15 4,74	1,75 1,15	1,66 1,15	1,75 5,79	1,75 5,74	1,66 5,79	1,66 5,74	1,75 1,26	1,66 1,26	1,95 1,15	1,95 5,79	1,95 5,74	5,79 1,26	5,74 1,26
1	0,5524	0,3556	>6	5,681	5,063	3,761	3,291	4,972	2,240	3,865	2,181	2,961	2,513	3,300	2,605	2,278	4,282
2	0,1479	0,1053	>6	5,651	5,029	3,431	3,297	5,000	2,229	3,896	2,163	2,923	2,484	3,276	2,620	2,263	4,298
3	0,0222	0,0849	2,983	2,032	2,260	2,968	3,139	3,076	4,110	3,126	4,479	4,358	5,019	3,883	4,402	2,375	3,963
4	0,0243	0,0796	2,505	2,328	2,122	3,179	3,305	2,872	4,251	3,108	4,513	4,284	5,047	3,893	4,403	2,318	4,054
5	0,0144	0,0650	3,809	1,839	2,634	2,826	2,853	3,275	3,906	3,200	4,403	4,447	5,023	3,858	4,397	2,465	3,826

6	0,0241	0,0605	2,011	2,602	1,958	3,519	3,244	2,836	4,246	3,075	4,461	4,320	4,839	3,961	4,364	2,304	4,021
7	0,0370	0,0490	3,444	2,190	2,624	3,231	3,227	2,921	4,212	3,096	4,468	4,335	5,272	3,950	4,369	2,316	3,992
8	0,0119	0,0442	3,610	1,858	2,499	2,824	2,815	3,387	3,879	3,344	4,419	4,428	4,980	3,825	4,484	2,497	3,732
9	0,0825	0,0413	6,323	>6	5,081	3,770	3,215	5,110	2,147	3,999	2,227	2,992	2,523	3,234	2,826	2,298	4,220
10	0,0273	0,0410	2,027	2,569	1,949	3,492	3,217	2,875	4,220	3,066	4,452	4,335	5,956	3,962	4,358	2,312	4,006
11	0,0134	0,0372	2,033	2,556	2,013	3,605	3,171	2,954	1,064	3,055	4,446	4,359	4,848	3,949	4,352	2,329	3,988
12	0,0158	0,0243	3,811	1,959	2,757	3,078	3,139	2,889	4,143	2,995	4,458	4,379	5,235	3,931	4,308	2,352	4,013
13	0,0267	0,0120	>8	>6	>5	3,832	3,418	4,844	2,400	3,697	2,214	2,974	2,648	3,412	2,436	2,225	4,356
expected NOE for 11R,14S									????		????		????		????		
observed experimental NOE				visible		visible			weak	weak	???			visible	weak	visible	visible

**11S,14R SSSS**		Distances	Me$_2$15/ Me19	Me$_2$15/ (*Z*)H20	H6ax/ Me$_2$15	H6eq/ Me$_2$15	H6ax/ H12	H6ax/ H13	H6eq/ H12	H6eq/ H13	H6ax/ Me18	H6eq/ Me18	H7/ Me$_2$15	H7/H12	H7/H13	H12/ Me18	H13/ Me18
		^{1}H Shifts	1,15 0,89	1,15 4,74	1,75 1,15	1,66 1,15	1,75 5,79	1,75 5,74	1,66 5,79	1,66 5,74	1,75 1,26	1,66 1,26	1,95 1,15	1,95 5,79	1,95 5,74	5,79 1,26	5,74 1,26
1	0,6730	0,3805	>4	2,420	3,374	1,952	4,388	3,534	3,278	2,527	4,238	4,524	4,174	2,351	3,974	2,307	4,070
2	0,0424	0,1550	>5	2,464	3,354	1,971	4,091	3,201	3,038	3,073	4,380	4,399	3,879	2,645	4,308	2,315	3,856
3	0,0672	0,1225	>4	2,287	3,225	3,050	4,001	3,140	2,924	3,100	4,424	4,352	5,247	2,645	4,329	2,339	3,860
4	0,0800	0,1184	>4	1,942	2,878	3,539	3,802	3,691	2,605	3,546	4,466	4,370	4,794	2,432	4,514	2,557	3,527
5	0,0355	0,0817	>5	3,554	3,554	2,158	4,305	3,192	3,268	2,695	4,299	4,495	3,074	2,618	4,099	2,262	4,024
6	0,0360	0,0539	>5	>7	>4	>6	2,391	4,424	3,196	5,251	2,976	1,905	5,309	3,951	4,382	2,315	3,996
7	0,0310	0,0480	>5	2,654	3,448	1,868	3,984	4,176	3,067	2,867	4,383	4,442	3,933	3,698	1,914	2,873	2,914
8	0,0349	0,0400	>5	2,675	3,525	1,922	4,246	3,954	3,291	2,593	4,329	4,477	3,991	3,705	1,850	2,668	3,254
expected NOE for 11S,14R									????	????							
observed experimental NOE				visible		visible			weak	weak	???			visible	weak	visible	visible

**11R,14R SSSS**		Distances	Me$_2$15/ Me19	Me$_2$15/ (*Z*)H20	H6ax/ Me$_2$15	H6eq/ Me$_2$15	H6ax/ H12	H6ax/ H13	H6eq/ H12	H6eq/ H13	H6ax/ Me18	H6eq/ Me18	H7/ Me$_2$15	H7/H12	H7/H13	H12/ Me18	H13/ Me18
		^{1}H Shifts	1,15 0,89	1,15 4,74	1,75 1,15	1,66 1,15	1,75 5,79	1,75 5,74	1,66 5,79	1,66 5,74	1,75 1,26	1,66 1,26	1,95 1,15	1,95 5,79	1,95 5,74	5,79 1,26	5,74 1,26
1	0,4067	0,1983	>4	2,546	3,502	2,000	4,479	3,535	3,414	2,433	1,958	2,565	4,122	2,397	3,878	3,611	2,011
2	0,2444	0,1659	>4	2,510	3,473	1,992	4,442	3,576	3,359	2,502	1,959	2,579	4,157	2,366	3,936	3,626	2,011
3	0,0291	0,1569	2,166	2,460	1,942	3,348	3,289	2,846	4,261	3,116	4,493	4,304	4,859	3,933	4,406	2,298	4,066
4	0,0358	0,1420	2,062	2,572	2,090	3,615	3,202	2,962	4,183	3,095	4,467	4,347	4,924	3,935	4,379	2,324	3,995
5	0,0630	0,0880	2,005	2,362	2,351	3,334	3,303	2,996	4,207	3,047	4,493	4,337	4,852	3,953	4,367	2,295	4,017
6	0,1237	0,0670	>6	2,499	3,466	1,990	4,455	3,545	3,360	2,473	1,989	2,607	4,153	2,408	3,931	3,605	2,014
7	0,0318	0,0480	>7	5,503	>5	3,874	3,279	4,918	2,299	3,763	2,214	3,029	2,788	3,370	2,587	2,245	4,323
8	0,0075	0,0348	2,331	>5	3,818	>5	2,306	4,122	2,791	5,043	4,427	4,430	>5	3,829	4,510	2,588	1,937
9	0,0128	0,0341	1,956	>5	4,492	>5	2,435	3,903	2,859	4,923	4,408	4,446	>5	3,887	4,428	3,518	2,050
10	0,0083	0,0237	2,185	2,478	1,900	3,361	3,261	2,840	4,239	3,051	4,461	4,330	4,800	3,963	4,371	2,276	4,054
11	0,0077	0,0195	2,049	2,547	2,065	3,616	3,200	2,938	4,188	3,073	4,463	4,349	4,909	3,939	4,351	2,320	3,979
12	0,0134	0,0120	>5	2,731	3,557	1,955	4,370	3,838	3,414	2,459	2,060	2,671	4,019	3,717	1,851	2,210	4,164
13	0,0159	0,0098	2,529	>5	3,955	>5	2,473	4,082	2,889	5,007	4,445	4,420	>5	3,895	4,477	3,539	1,982
expected NOE for 11R,14R																???	
observed experimental NOE				visible		visible			weak	weak	???			visible	weak	visible	visible

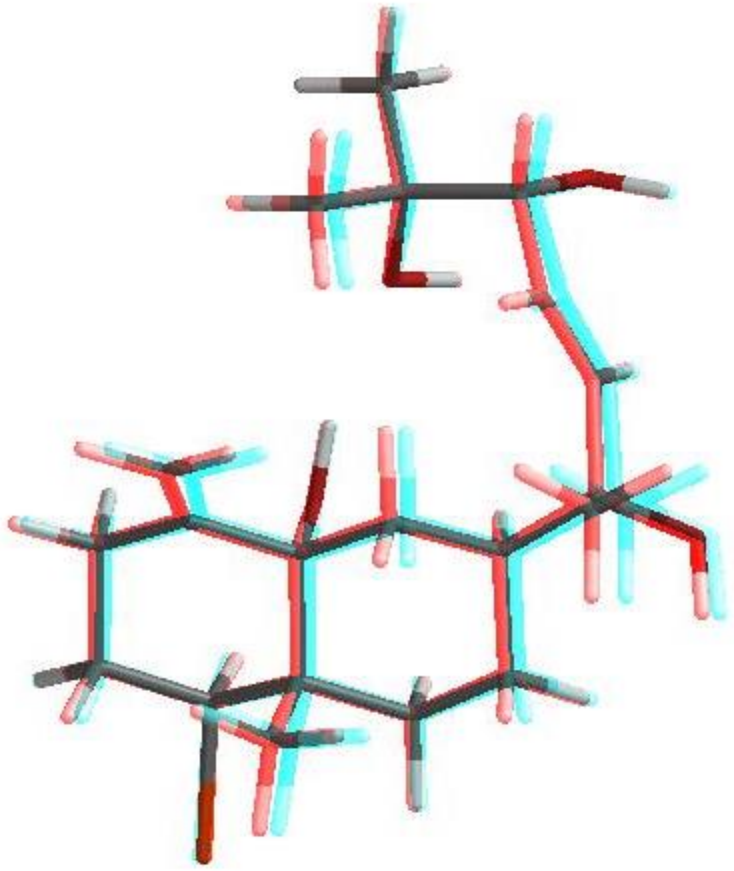	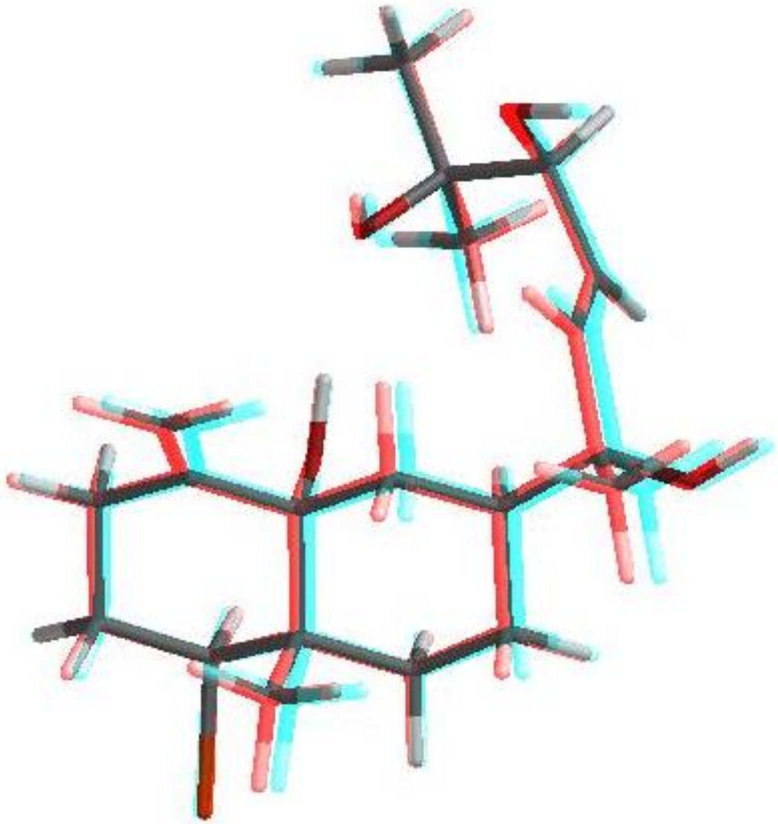
**(11R,14R, SSSS) isomer**	**(11R,14S, SSSS) isomer**

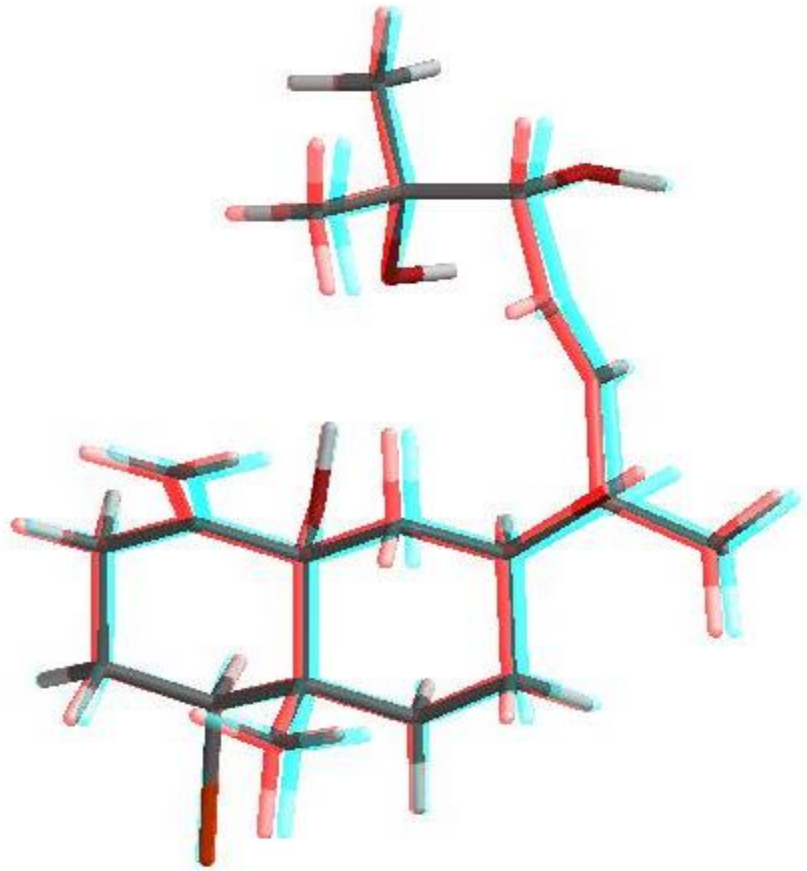	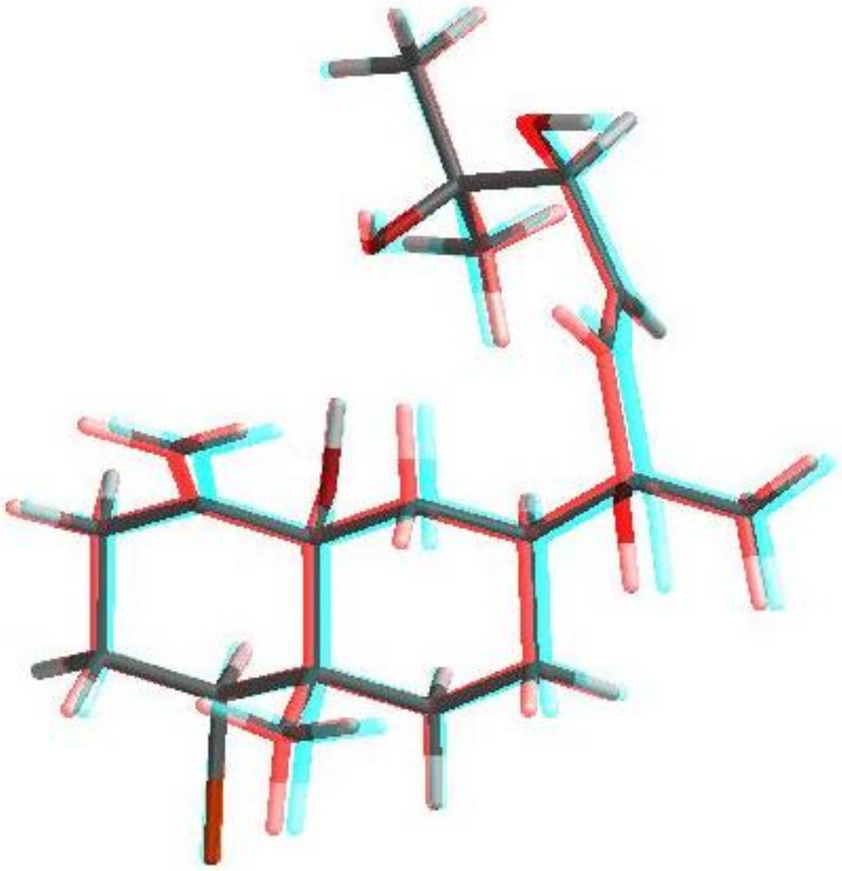
**(11S,14R, SSSS) isomer**	**(11S,14S, SSSS) isomer**

Figure S9: Stereo views of the four (11,14)-diastereomers with (SSSS)-configured decalin core.

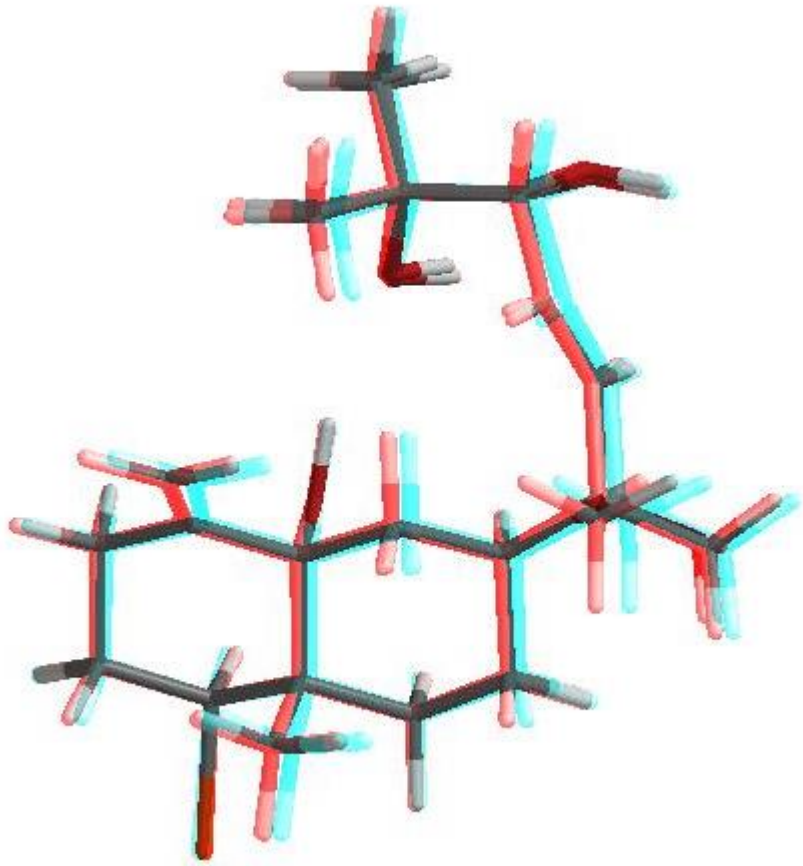	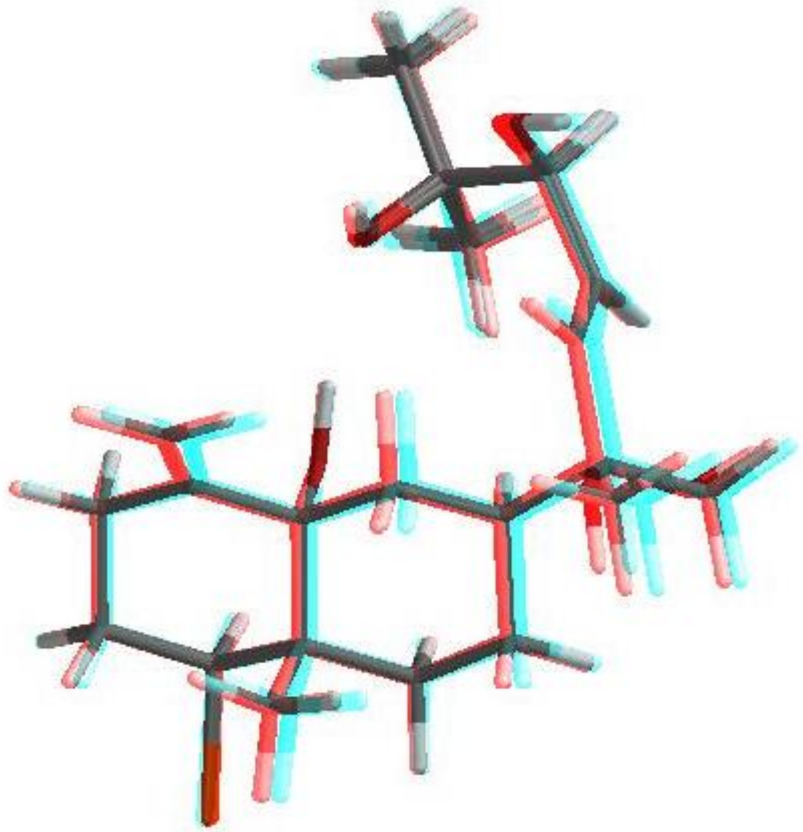
**(11R,14R) aligned with (11S,14R) isomer**	**(11S,14S) aligned with (11R,14S) isomer**

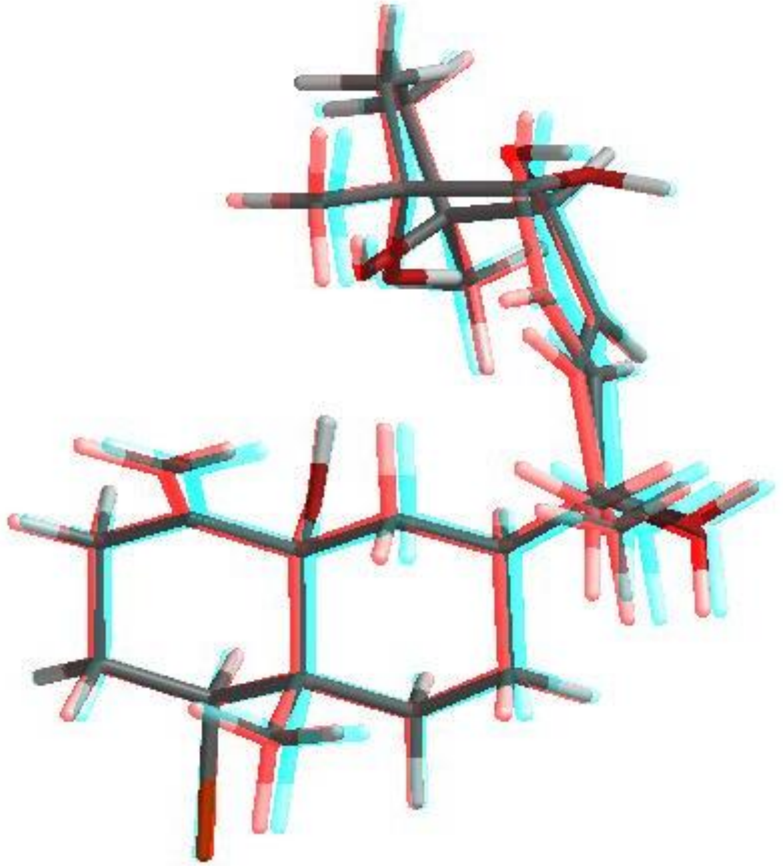	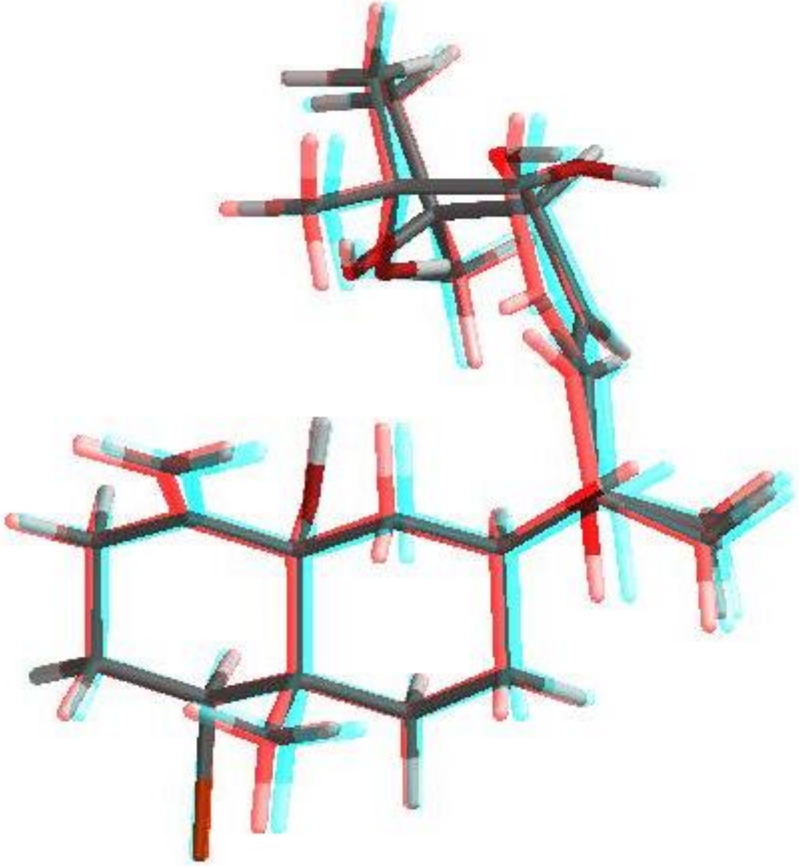
**(11R,14R) aligned with (11R,14S) isomer**	**(11S,14S) aligned with (11S,14R) isomer**

Figure S10: Stereo views of the four pairs of aligned diastereomers of 11,14-dihydroaplysiatetrols (**5**) with pseudoenantiomeric side chains.

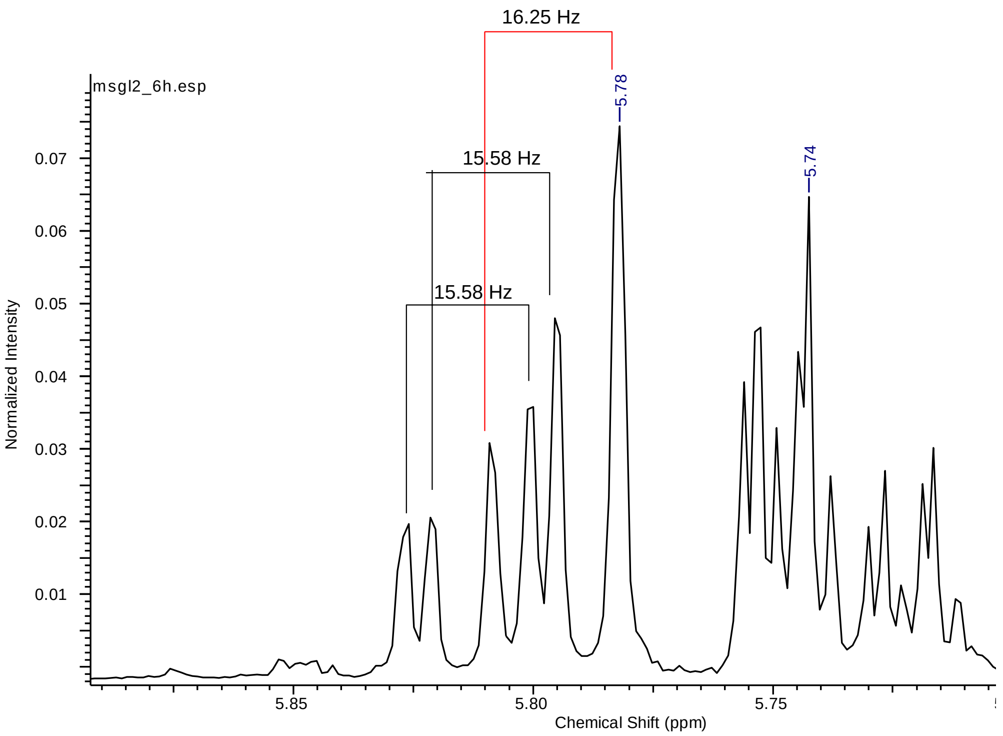

Figure S11: According to the ^{1}H NMR double bond signal of H-12 and H-13, **3** is a mixture of three similar compounds, each with *trans*-configured side chains.

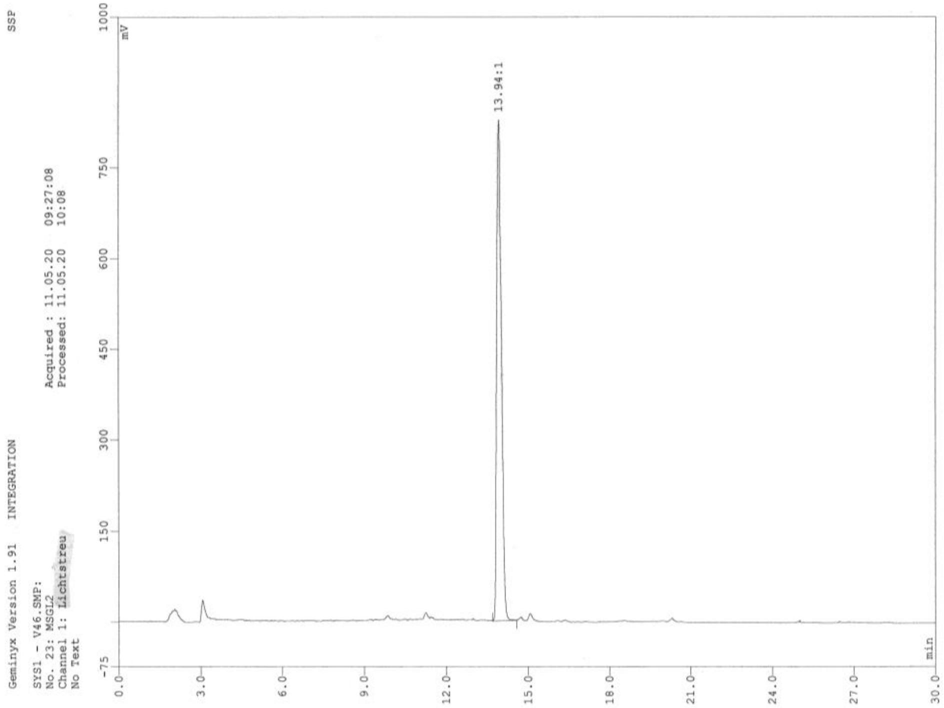

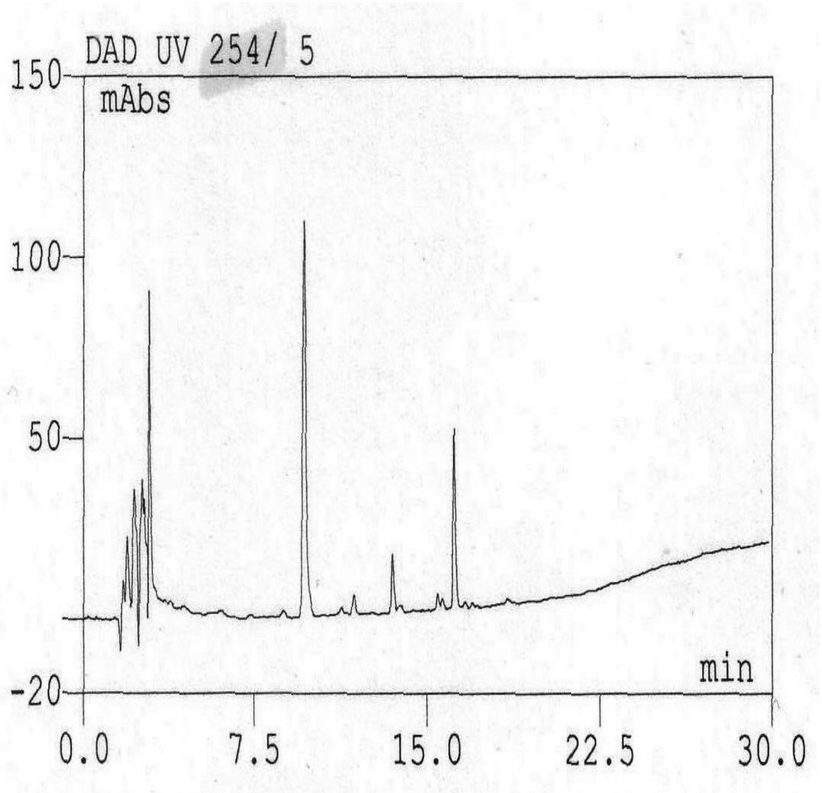

Figure S12: Preparative HPLC of the MSGL2 (**5a-c**) mixture on Nucleodur C18; detection by light scattering (figure on the left) and UV absorption (on the right).

HPLC: Instrumentelle Analytik Goebel GmbH. HPLC Pump 420, Autosampler SA 360, HPLC Detector Celeno DAD UV, light scattering detector ELSD-Sedex 85, ERC.

Column: Nucleodur 100-5 C18 ec, 250 mm x 3 mm. **Solvent system**: A = H_2O + 0.1 % TFA. B = MeCN + 0.1 % TFA. Flow rate 0.5 ml/min; gradient: start with 20% B, 00-20 min: 20% B to 100% B, 20-30 min: 100% B. **Detection**: light scattering and DAD 200 – 610 nm.

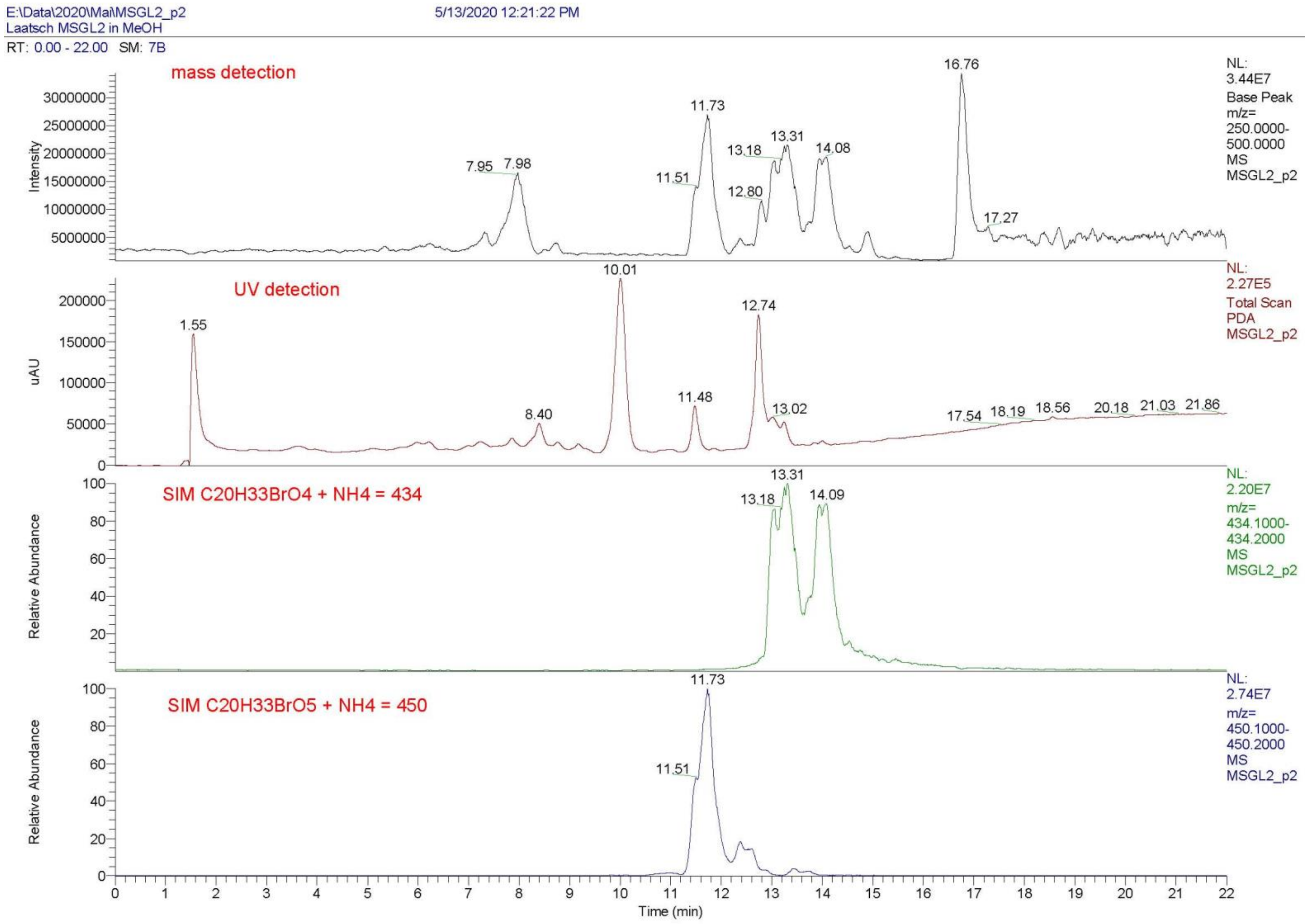

Figure S13: HPLC/MS of the MSGL2 mixture; detection by MS and SIM (selective ion monitoring)

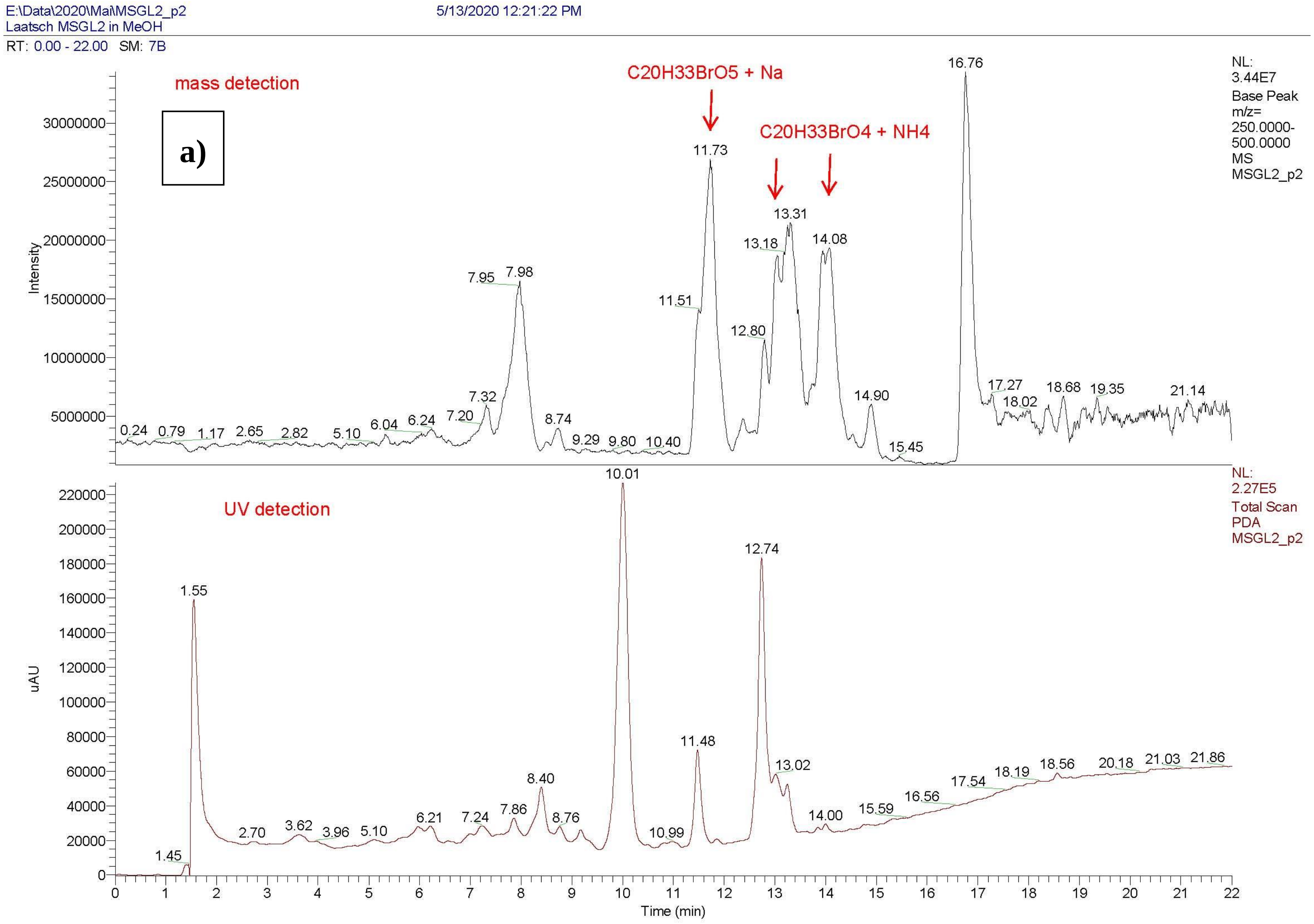
E:\Data\2020\Mai\MSGL2_p2
Laatsch MSGL2 in MeOH
5/13/2020 12:21:22 PM
RT: 0.00 - 22.00 SM: 7B
mass detection
a)
C20H33BrO5 + Na
C20H33BrO4 + NH4
NL: 3.44E7 Base Peak m/z= 250.0000-500.0000 MS MSGL2_p2
Intensity
UV detection
NL: 2.27E5 Total Scan PDA MSGL2_p2
uAU
Time (min)

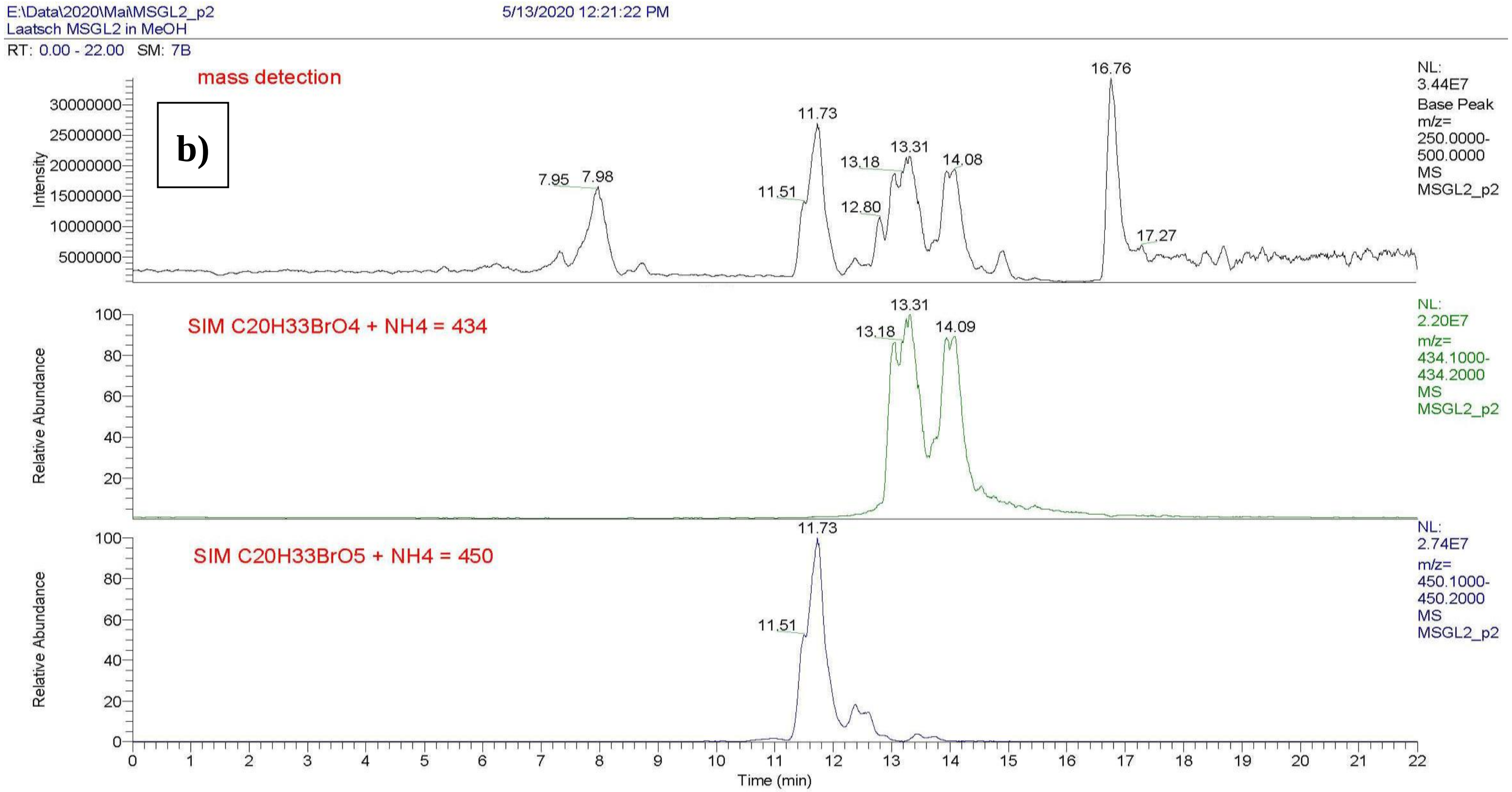

Figure S14: Analytical HPLC/HRMS of dihydroaplysia-5,11,14,15-tetrol (**3**) mixture with a) mass and UV detection and b) SIM mode at 434 and 450 Dalton.

HPLC instrument: Accela (Thermo) with HPLC pump, autoinjector and Surveyor PDA. column: Kinetex (Phenomenx) C18 150 x 2.1 mm, 5 µm particle size. eluent A: H_2O + 0.05% HCOOH; eluent B: MeOH + 0.05% HCOOH; gradient: 0 min 80/20 A/B, 0-15 min to 0/100 A/B, 15-22 min 0/100 A/B; flow: 0.2 mL/min, injection volume: 10 µL; PDA: 200-600 nm. MS instrument: LTQ Orbitrap XL (Thermo), Ionisation: (+)-ESI; mass analyser: Orbitrap (R ~ 60.000), mass range: *m/z* 100 – 2000

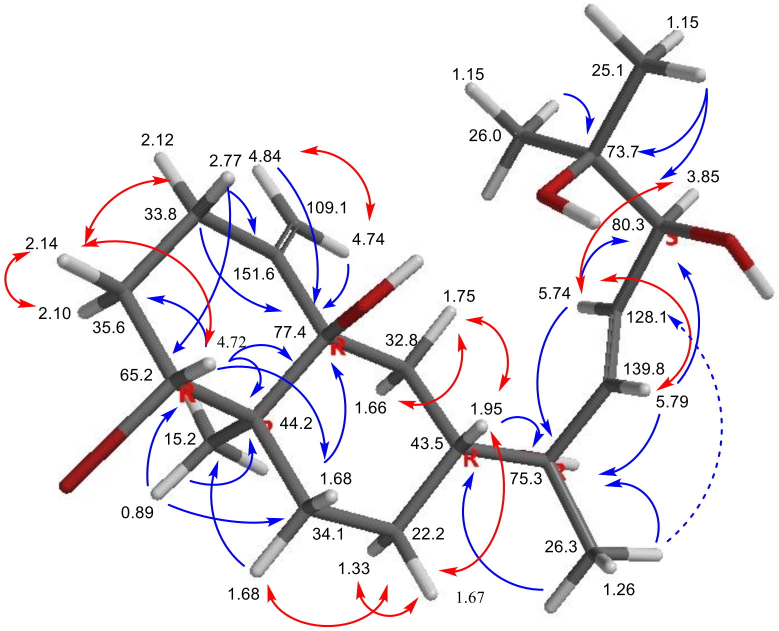

Figure S15: Structure of dihydroaplysia-5,11,14,15-tetrol (**5**) with all experimental H,H COSY ( ) and HMBC ( ) correlations, shown for the (1*R*,5*R*,7*R*,10*R*,11*R*,14*S*)-stereoisomer.

8 Alternative structures of 5-*epi*-maneolactone (6)

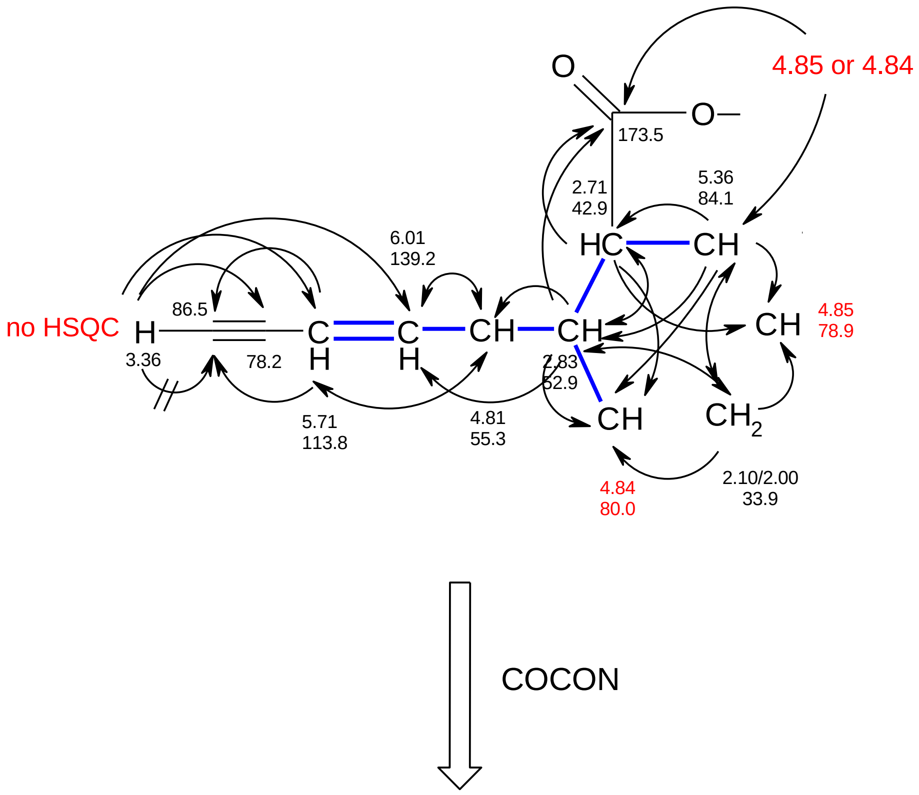

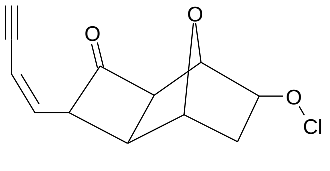

a

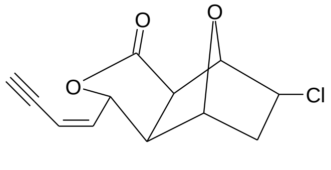

b

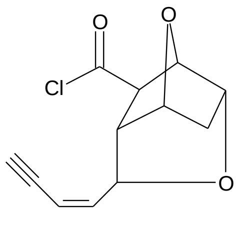

c

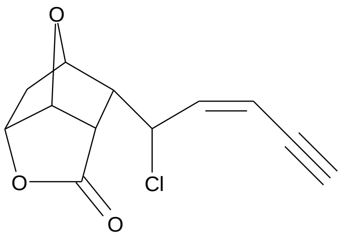

d = 4

Figure S16: Alternative structures of 5-*epi*-maneolactone (**6**), calculated with COCON from the experimental COSY and HMBC correlations, using ((*Z*)-pent-2-en-4-ynyl)-cyclohexane as core structure.

9 Crystal structure determination details of 7-acetyl-aplysiol (2) and 5-*epi*-Maneolactone (6)

Data collection was done on two dual source equipped *Bruker D8 Venture* four-circle-diffractometer from *Bruker AXS GmbH*; used X-ray sources: microfocus *IµS 2.0* Mo (for **6**) and microfocus *IµS 3.0* Mo (for **2**) from *Incoatec GmbH* with mirror optics *HELIOS* and single-hole collimator from *Bruker AXS GmbH;* used detector: *Photon III CE14* (Cu/Mo) and Photon III HE (Ag/Mo) from *Bruker AXS GmbH.*

Used programs: *APEX3 Suite* (v2018.7-2) for data collection and therein integrated programs *SAINT* V8.38A (Integration) und *SADABS* 2016/2 (Absorption correction) from *Bruker AXS GmbH*; structure solution was done with *SHELXT*, refinement with *SHELXL*-2018/3 (Both: G.M. Sheldrick, *Acta Cryst*. **2008,** *A64*, 112-122.); OLEX^2 was used for data finalization (O.V. Dolomanov, L.J. Bourhis, R.J Gildea, J.A.K. Howard, H. Puschmann, *J. Appl. Cryst*. **2009,** *42*, 339-341.).

Special Utilities: *SMZ1270* stereomicroscope from *Nikon Metrology GmbH* was used for sample preparation; crystals were mounted on *MicroMounts* or *MicroLoops* from *MiTeGen* in NVH oil; crystals were cooled to given temperature with *Cryostream 800* from *Oxford Cryosystems.*

Table S7: Crystal data and structure refinement for **2** and **6**.

	**2**	**6**
Empirical formula	$C_{14}H_{21}BrO_2$	$C_{12}H_{11}ClO_3$
Formula weight	301.22	238.66
Temperature/K	110	100
Crystal system	orthorhombic	monoclinic
Space group	$P2_12_12_1$	$P2_1$
a/Å	6.3955(10)	7.4664(6)
b/Å	11.5862(18)	5.6266(4)
c/Å	18.379(3)	12.6915(10)
α/°	90	90
β/°	90	97.449(3)
γ/°	90	90
Volume/Å^3	1361.9(4)	528.68(7)
Z	4	2
ρ_{calc}g/cm^3	1.469	1.499
μ/mm^{-1}	3.007	0.348
F(000)	624.0	248.0
Crystal size/mm^3	0.161 × 0.13 × 0.052	0.405 × 0.297 × 0.124
Radiation	MoKα (λ = 0.71073)	MoKα (λ = 0.71073)
2Θ range for data collection/°	4.16 to 56.7	5.502 to 59.2
Index ranges	$-8 \leq h \leq 8$, $-15 \leq k \leq 15$,	$-10 \leq h \leq 10$, $-7 \leq k \leq 7$,

	$-24 \leq l \leq 24$	$-17 \leq l \leq 17$
Reflections collected	18643	22890
Independent reflections	3400 [R_{int} = 0.0376, R_{sigma} = 0.0282]	2897 [R_{int} = 0.0185, R_{sigma} = 0.0140]
Data/restraints/parameters	3400/0/167	2897/1/149
Goodness-of-fit on F^2	1.101	1.121
Final R indexes [I>=2σ (I)]	R_1 = 0.0315, wR_2 = 0.0718	R_1 = 0.0204, wR_2 = 0.0559
Final R indexes [all data]	R_1 = 0.0351, wR_2 = 0.0734	R_1 = 0.0205, wR_2 = 0.0559
Largest diff. peak/hole / e $Å^{-3}$	0.57/-0.66	0.28/-0.18
Flack parameter	0.001(5)	-0.014(7)

Display Report

Analysis Info		Acquisition Date	15.02.2016 08:03:13	
Analysis Name	Z:\Data\2016\1602\sam150216\neg\mshaaba00048 low_1_01_86199.d			
Method	hystar_nl.m	Operator	BDAL@DE	
Sample Name	mshaaba00048 low	Instrument / Ser#	micrOTOF	10237
Comment				

Acquisition Parameter

Source Type	ESI	Ion Polarity	Negative	Set Nebulizer	1.6 Bar
Focus	Not active			Set Dry Heater	180 °C
Scan Begin	50 m/z	Set Capillary	3800 V	Set Dry Gas	8.0 l/min
Scan End	1600 m/z	Set End Plate Offset	-500 V	Set Divert Valve	Source

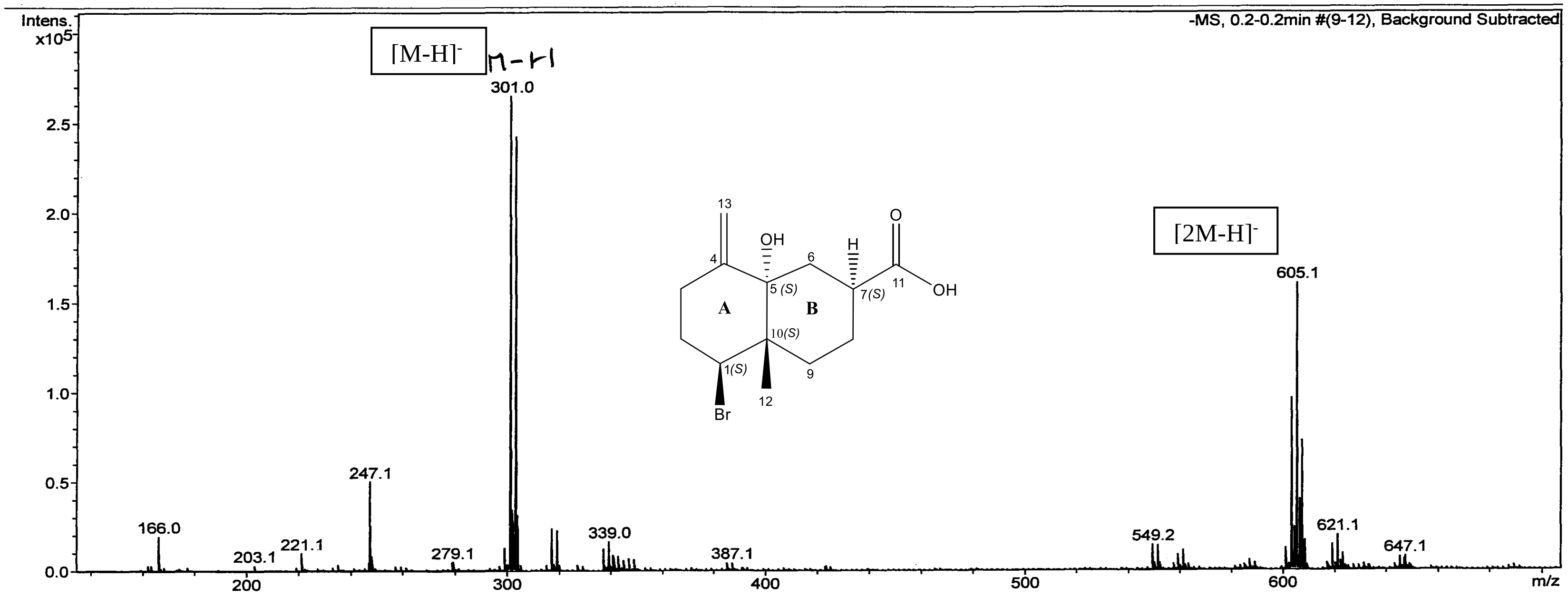

Bruker Compass DataAnalysis 4.0 printed: 15.02.2016 09:23:58 Page 1 of 1

Figure S17: (-)-ESI mass spectrum of aplysiolic acid (**1**)

Mass Spectrum SmartFormula Report

Analysis Info		Acquisition Date	15.02.2016 08:03:13	
Analysis Name	Z:\Data\2016\1602\sam150216\neg\mshaaba00048 low_1_01_86199.d			
Method	hystar_nl.m	Operator	BDAL@DE	
Sample Name	mshaaba00048 low	Instrument / Ser#	micrOTOF	10237
Comment				

Acquisition Parameter

Source Type	ESI	Ion Polarity	Negative	Set Nebulizer	1.6 Bar
Focus	Not active			Set Dry Heater	180 °C
Scan Begin	50 m/z	Set Capillary	3800 V	Set Dry Gas	8.0 l/min
Scan End	1600 m/z	Set End Plate Offset	-500 V	Set Divert Valve	Source

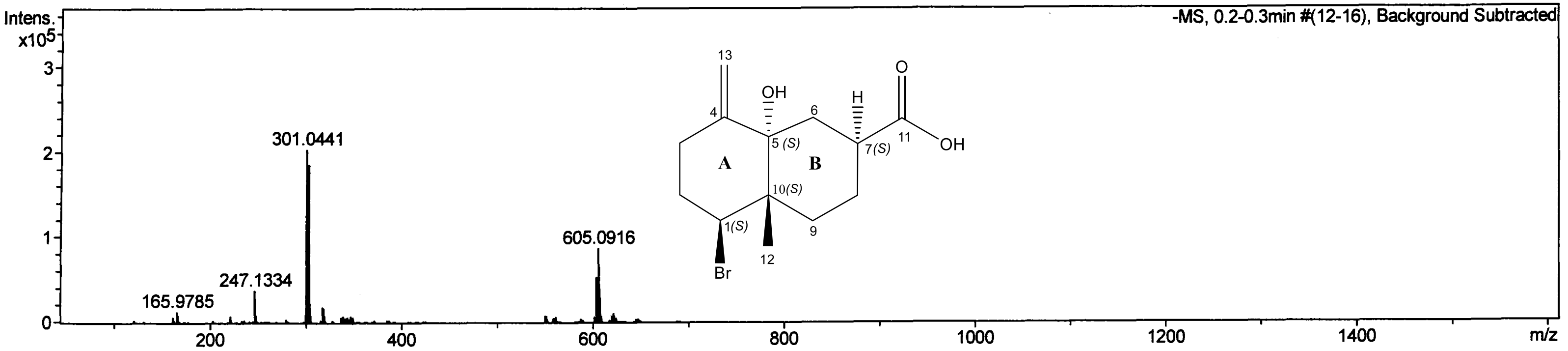

Meas. m/z	#	Formula	m/z	err [ppm]	Mean err [ppm]	rdb	N-Rule	e⁻ Conf	mSigma	Std I	Std Mean m/z	Std I VarNorm	Std m/z Diff	Std Comb Dev
301.0441														
	1	C 9 H 14 Br N 6 O	301.0418	-7.5	-7.9	5.5	ok	even	29.98	0.0240	0.0025	0.0069	0.0018	0.8397
	2	C 13 H 18 Br O 3	301.0445	1.4	1.0	4.5	ok	even	34.83	0.0259	0.0009	0.0071	0.0018	0.7422
	3	C 8 H 18 Br N 2 O 5	301.0405	-12.0	-12.4	0.5	ok	even	37.29	0.0374	0.0038	0.0124	0.0018	0.9277

Figure S18: (-)-ESI HR mass spectrum of aplysiolic acid (**1**)

Display Report

Analysis Info

Analysis Name	Z:\Data\2016\1602\sam150216\neg\mshaaba00048 low_1_01_86199.d	Acquisition Date	15.02.2016 08:03:13
Method	hystar_nl.m	Operator	BDAL@DE
Sample Name	mshaaba00048 low	Instrument / Ser#	micrOTOF 10237
Comment			

Acquisition Parameter

Source Type	ESI	Ion Polarity	Negative	Set Nebulizer	1.6 Bar
Focus	Not active			Set Dry Heater	180 °C
Scan Begin	50 m/z	Set Capillary	3800 V	Set Dry Gas	8.0 l/min
Scan End	1600 m/z	Set End Plate Offset	-500 V	Set Divert Valve	Source

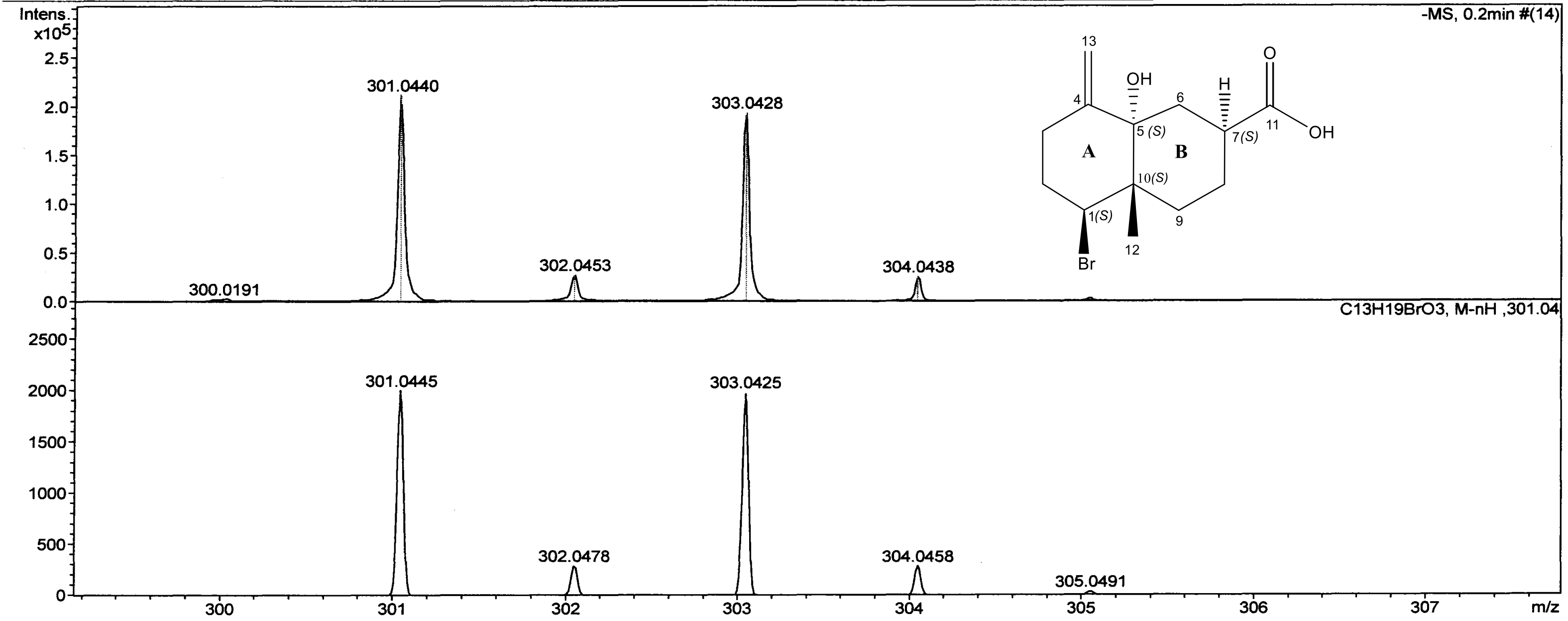

Bruker Compass DataAnalysis 4.0 printed: 24.02.2016 13:00:10 Page 1 of 1

Figure S19: (-)-ESI HR mass spectrum of aplysiolic acid (**1**)

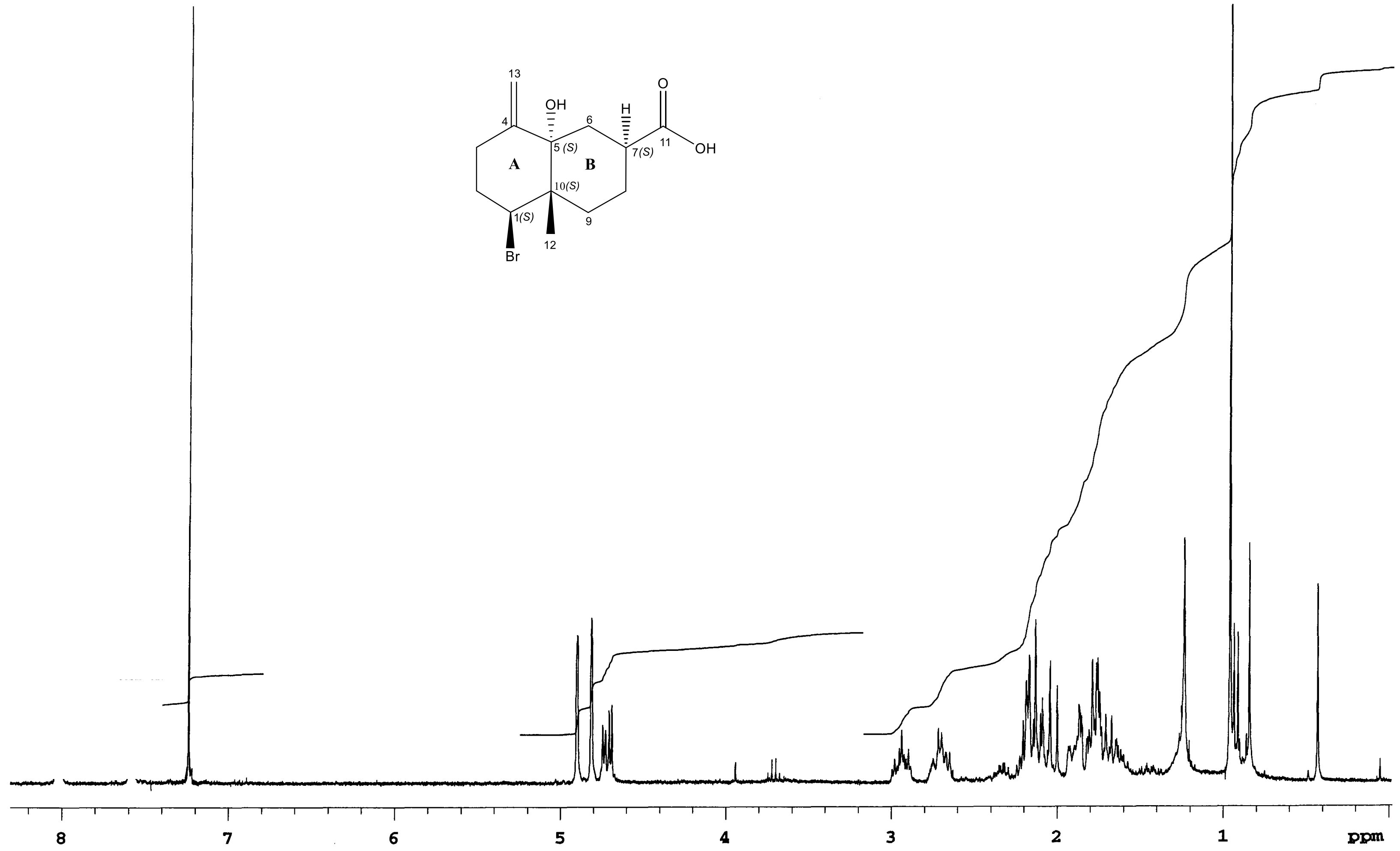

Figure S20: ^{1}H NMR spectrum (300 MHz, $CDCl_3$) of aplysiolic acid (**1**)

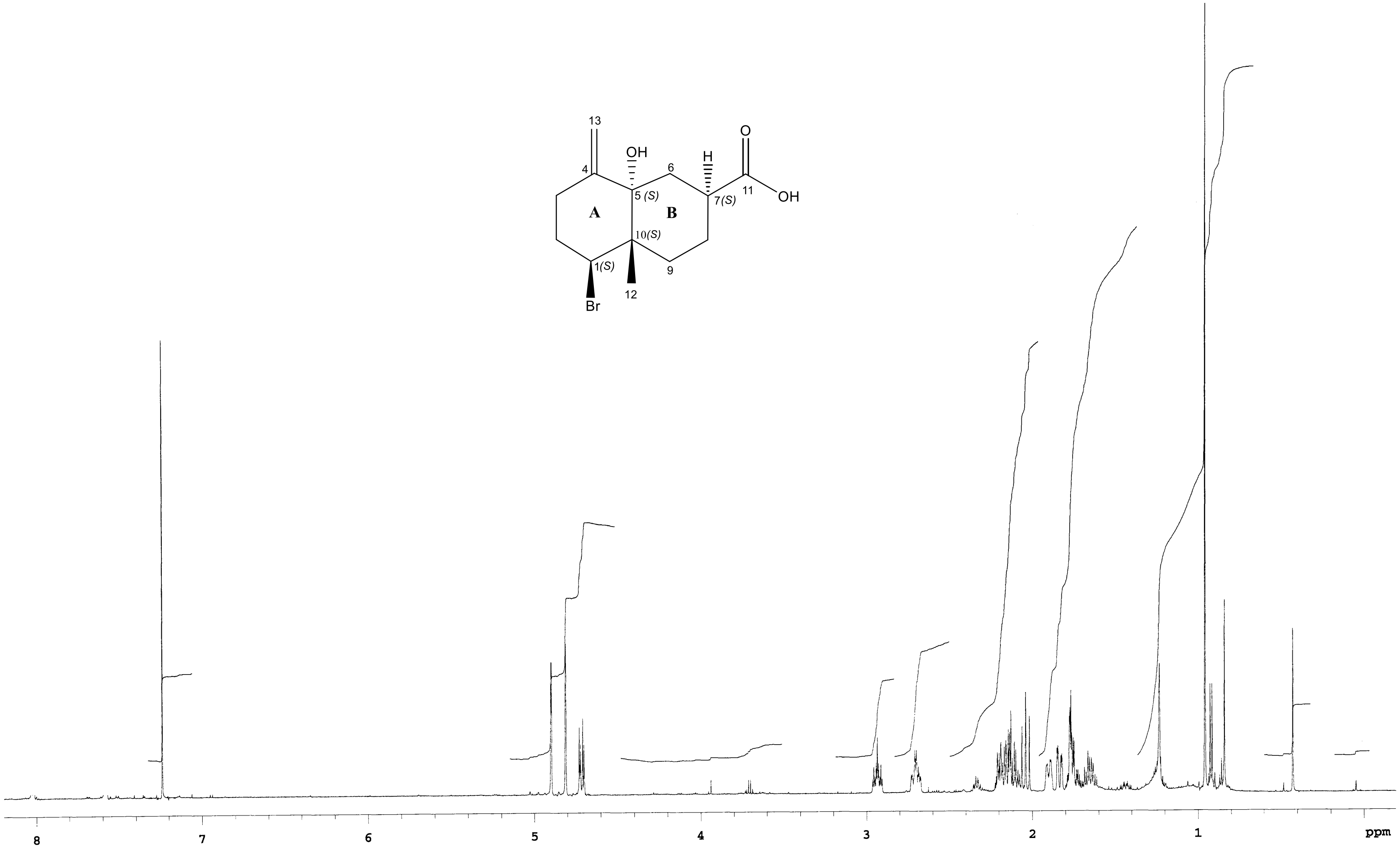

Figure S21: ^{1}H NMR spectrum (600 MHz, $CDCl_3$) of aplysiolic acid (**1**)

MSGL1a cdcl3
Shaaban / Laatsch / mw

SPECTRAL LINES FOR TH= 4.3
FROM -2.5 PPM TO 197.5 PPM
RFL= 1931.6 RFP= 0.0

INDEX	FREQ	PPM	HEIGHT
1	22748.5	180.98	5.8
2	18661.1	148.46	18.5
3	16246.4	129.25	13.3
4	15967.4	127.03	15.7
5	13852.8	110.21	25.5
6	10947.0	87.09	5.0
7	9709.7	77.25	392.8
8	9677.9	77.00	400.0
9	9646.2	76.74	391.3
10	9580.8	76.22	21.3
11	7911.3	62.94	32.3
12	6084.0	48.40	6.0
13	5400.0	42.96	21.2
14	5202.0	41.39	8.0
15	4806.6	38.24	17.3
16	4616.5	36.73	7.7
17	4291.6	34.14	30.0
18	4264.9	33.93	29.2
19	4089.0	32.53	36.1
20	4017.7	31.96	34.7
21	3740.1	29.76	5.0
22	3597.9	28.62	7.3
23	2956.7	23.52	30.2
24	2376.3	18.91	8.1
25	1855.7	14.76	36.0

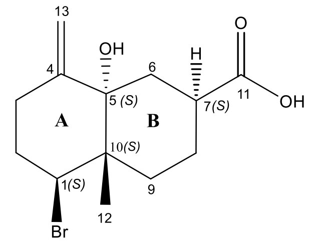

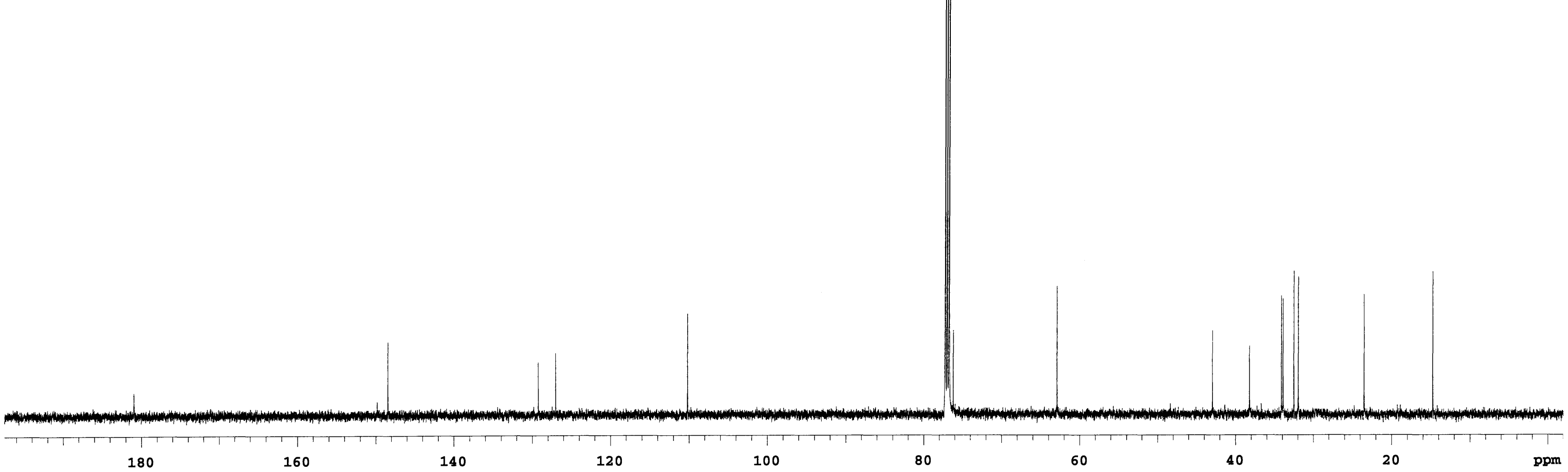

Figure S22: ^{13}C NMR spectrum (125 MHz, $CDCl_3$) of aplysiolic acid (**1**)

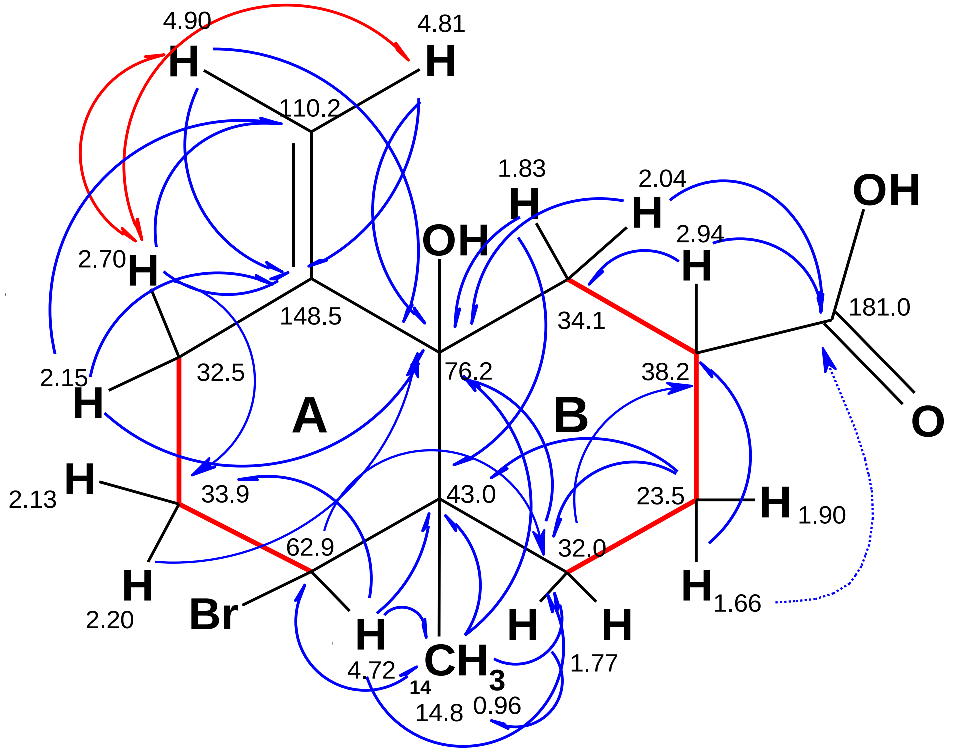

Figure S23: H,H COSY (^{3}J —, ^{4}J ⌒) and HMBC (⌒) correlations of aplysiolic acid (**1**). Geminal COSY couplings are not depicted.

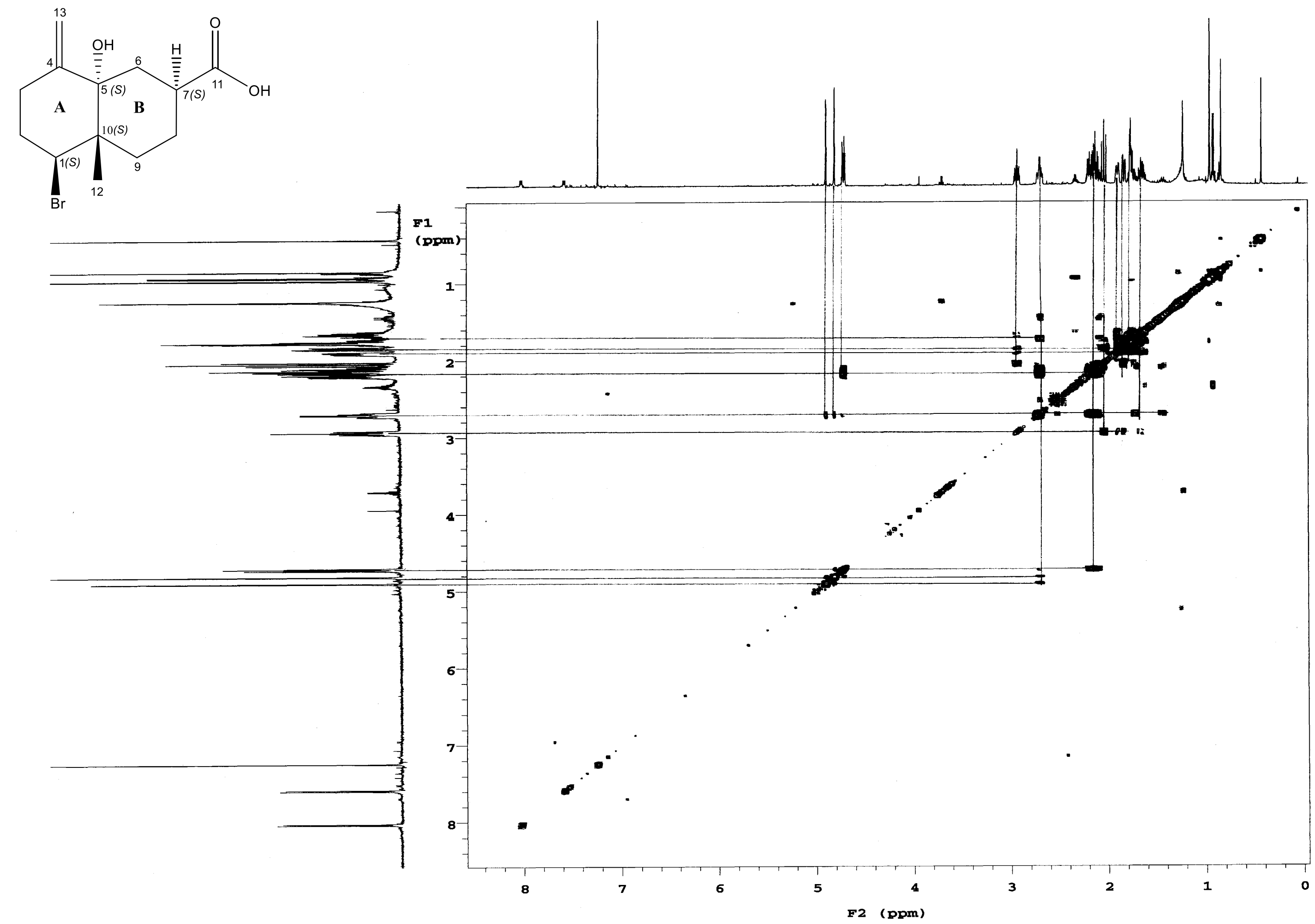

Figure S24: H,H COSY spectrum (500 MHz, $CDCl_3$) of aplysiolic acid (**1**)

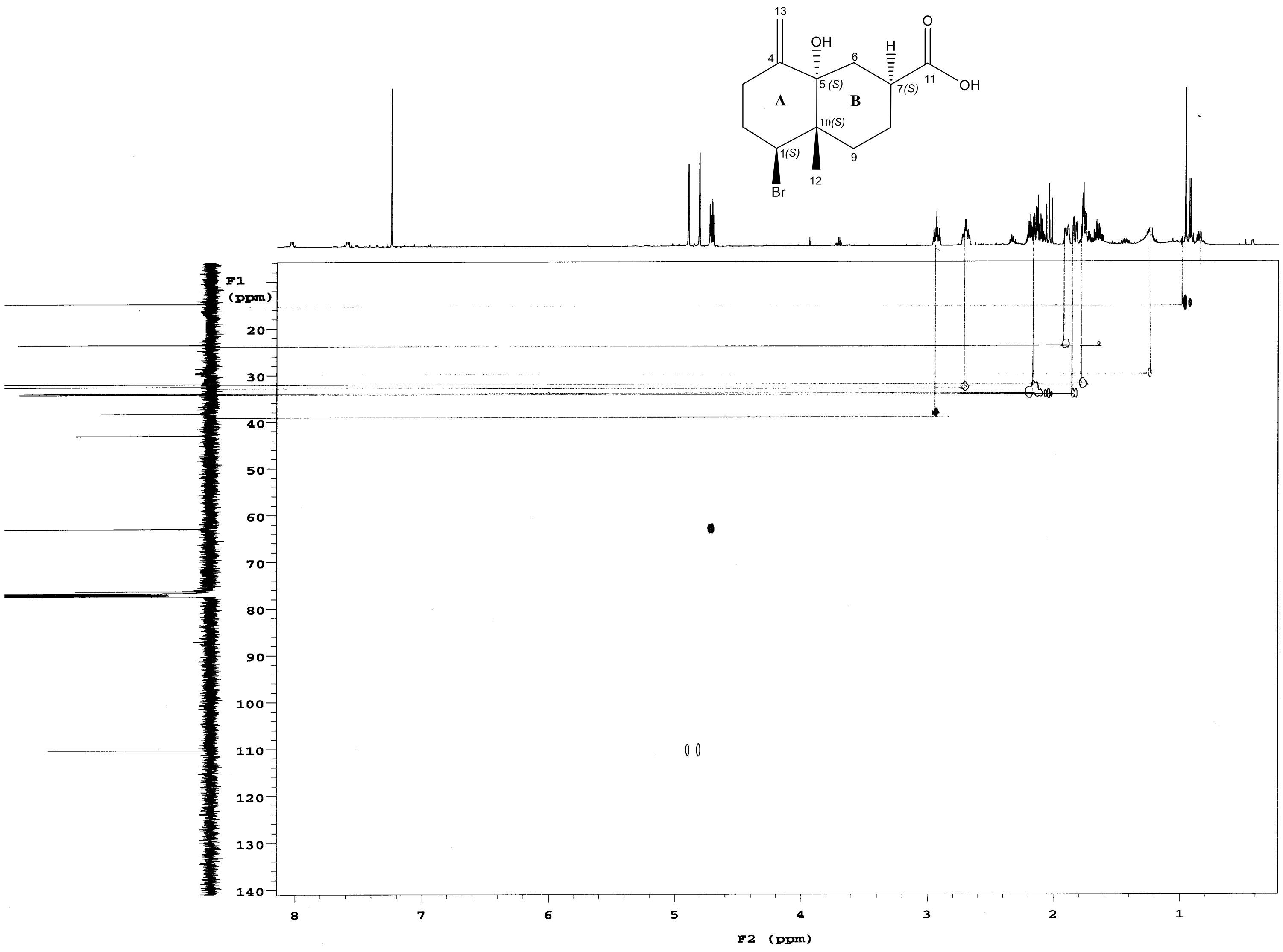

Figure S25: HMQC spectrum (500 MHz, $CDCl_3$) of aplysiolic acid (**1**)

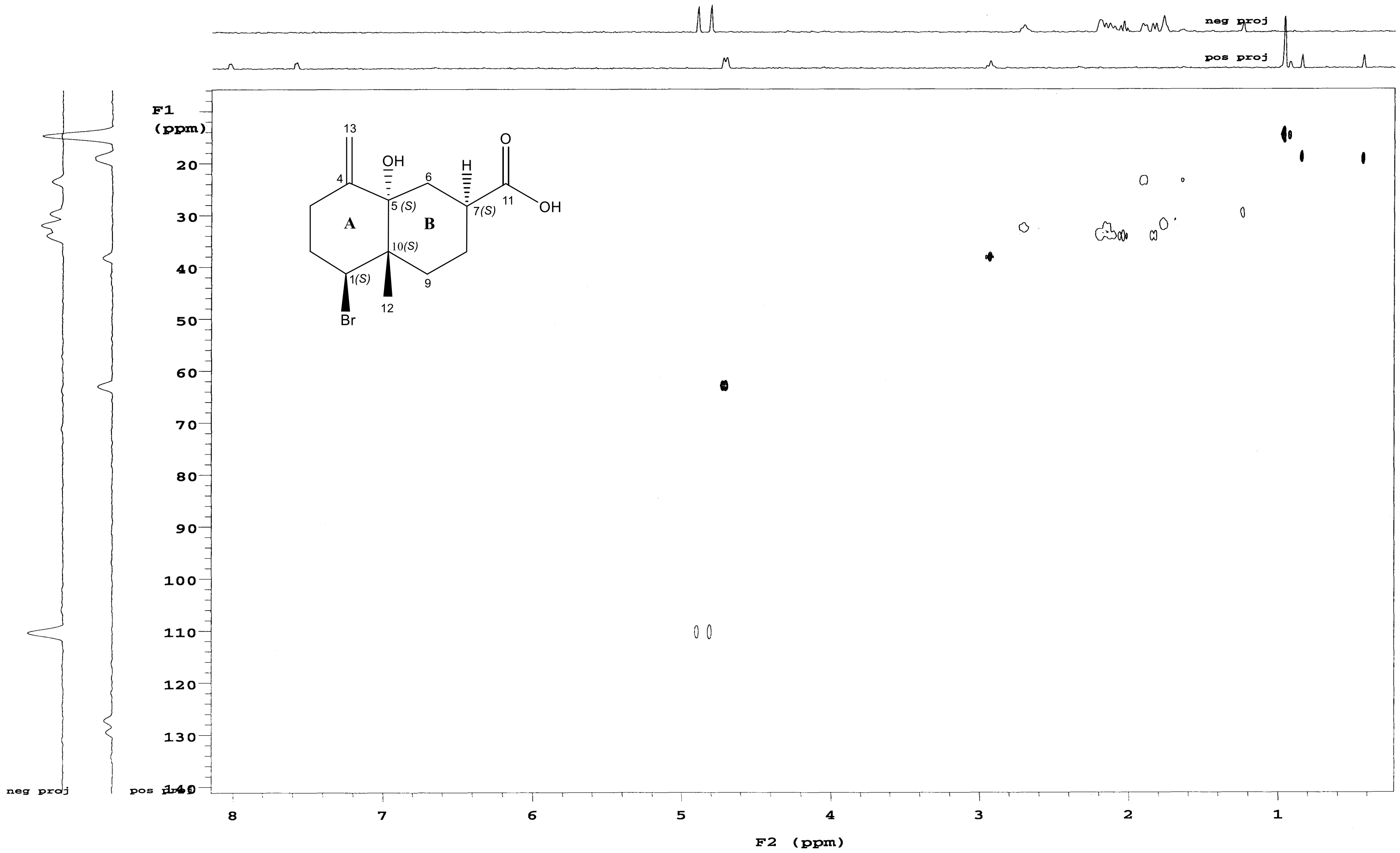

Figure S26: HSQC spectrum (500 MHz, $CDCl_3$) of aplysiolic acid (**1**)

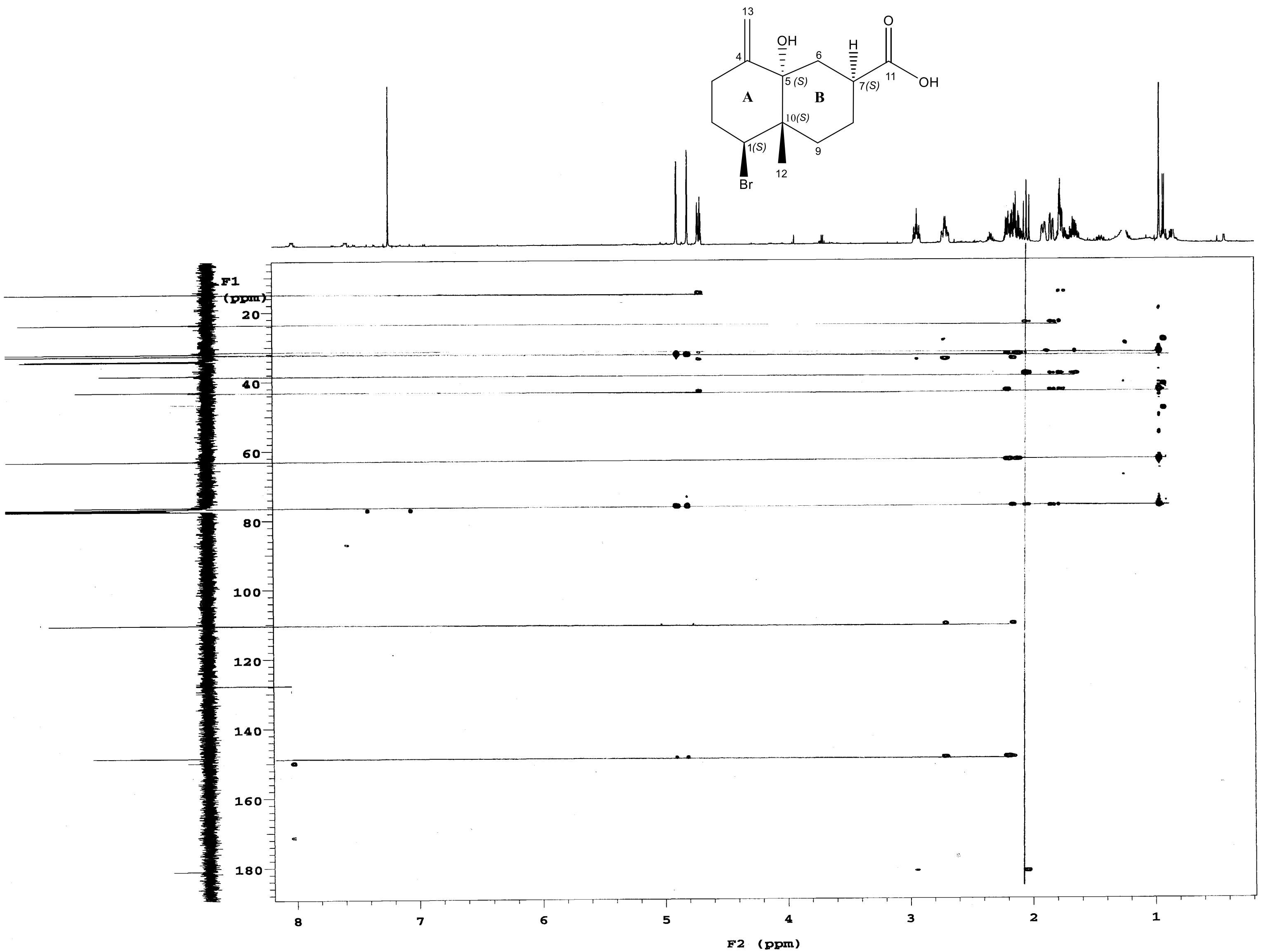

Figure S27: HMBC spectrum (500 MHz, $CDCl_3$) of aplysiolic acid (**1**)

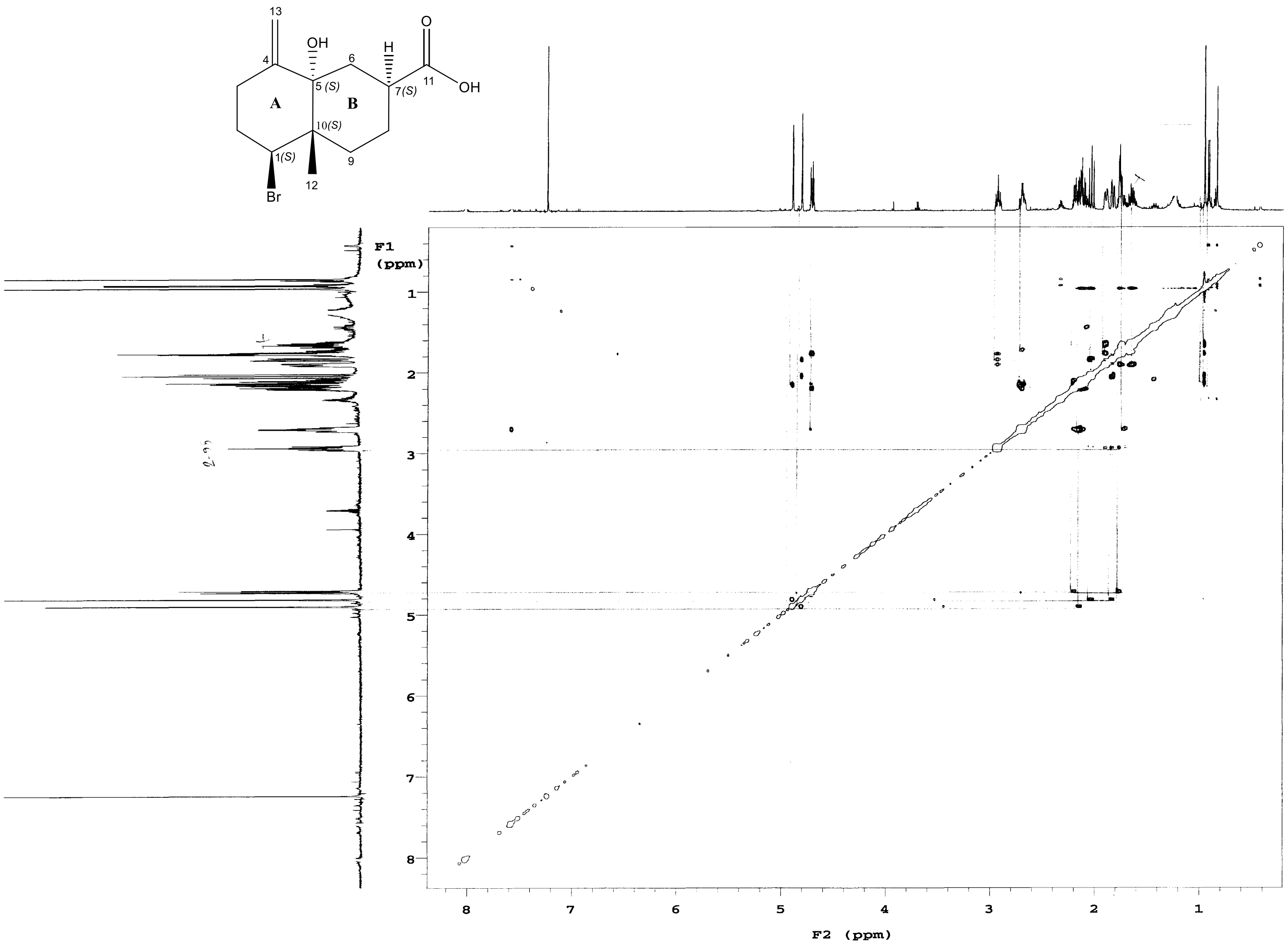

Figure S28: NOESY spectrum (500 MHz, $CDCl_3$) of aplysiolic acid (**1**)

Display Report

Analysis Info

Analysis Name	Z:\Data\2016\1602\sam250216\mshaaba00065 low_1_01_86571.d	Acquisition Date	25.02.2016 10:31:18
Method	hystar_pl.m	Operator	BDAL@DE
Sample Name	mshaaba00065 low	Instrument / Ser#	micrOTOF 10237
Comment			

Acquisition Parameter

Source Type	ESI	Ion Polarity	Positive	Set Nebulizer	1.2 Bar
Focus	Not active			Set Dry Heater	180 °C
Scan Begin	50 m/z	Set Capillary	4500 V	Set Dry Gas	6.0 l/min
Scan End	1600 m/z	Set End Plate Offset	-500 V	Set Divert Valve	Source

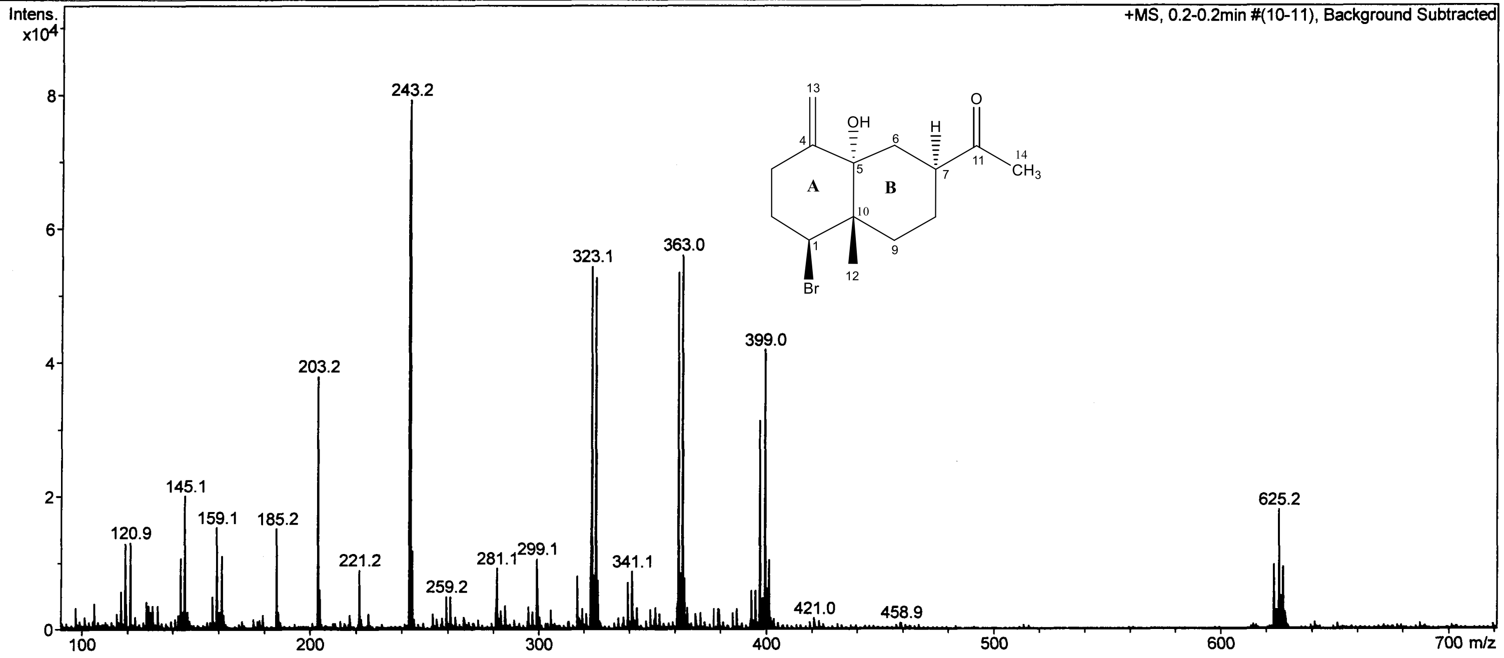

Bruker Compass DataAnalysis 4.0 printed: 25.02.2016 11:20:56 Page 1 of 1

Figure S29: (+)-ESI mass spectrum of 7-acetyl-aplysiol (**2**)

Display Report

Analysis Info

Analysis Name	Z:\Data\2016\1602\sam250216\neg\mshaaba00065 low_1_01_86552.d	Acquisition Date	25.02.2016 07:54:04
Method	hystar_nl.m	Operator	BDAL@DE
Sample Name	mshaaba00065 low	Instrument / Ser#	micrOTOF 10237
Comment			

Acquisition Parameter

Source Type	ESI	Ion Polarity	Negative	Set Nebulizer	1.6 Bar
Focus	Not active			Set Dry Heater	180 °C
Scan Begin	50 m/z	Set Capillary	3800 V	Set Dry Gas	8.0 l/min
Scan End	1600 m/z	Set End Plate Offset	-500 V	Set Divert Valve	Source

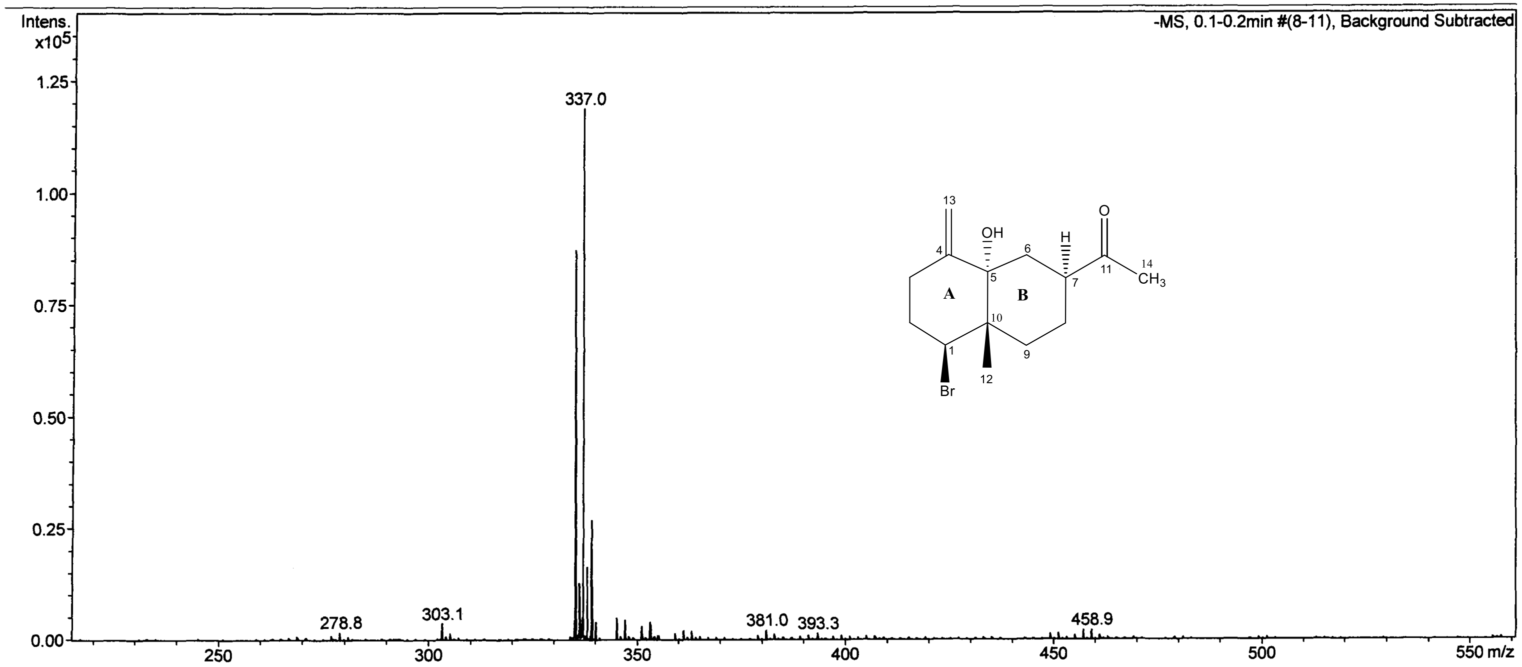

Bruker Compass DataAnalysis 4.0 printed: 25.02.2016 09:29:13 Page 1 of 1

Figure S30. (-)-ESI mass spectrum of 7-acetyl-aplysiol (**2**)

Display Report

Analysis Info

Analysis Name	Z:\Data\2016\1602\sam250216\neg\mshaaba00065 low_1_01_86552.d	Acquisition Date	25.02.2016 07:54:04
Method	hystar_nl.m	Operator	BDAL@DE
Sample Name	mshaaba00065 low	Instrument / Ser#	micrOTOF 10237
Comment			

Acquisition Parameter

Source Type	ESI	Ion Polarity	Negative	Set Nebulizer	1.6 Bar
Focus	Not active			Set Dry Heater	180 °C
Scan Begin	50 m/z	Set Capillary	3800 V	Set Dry Gas	8.0 l/min
Scan End	1600 m/z	Set End Plate Offset	-500 V	Set Divert Valve	Source

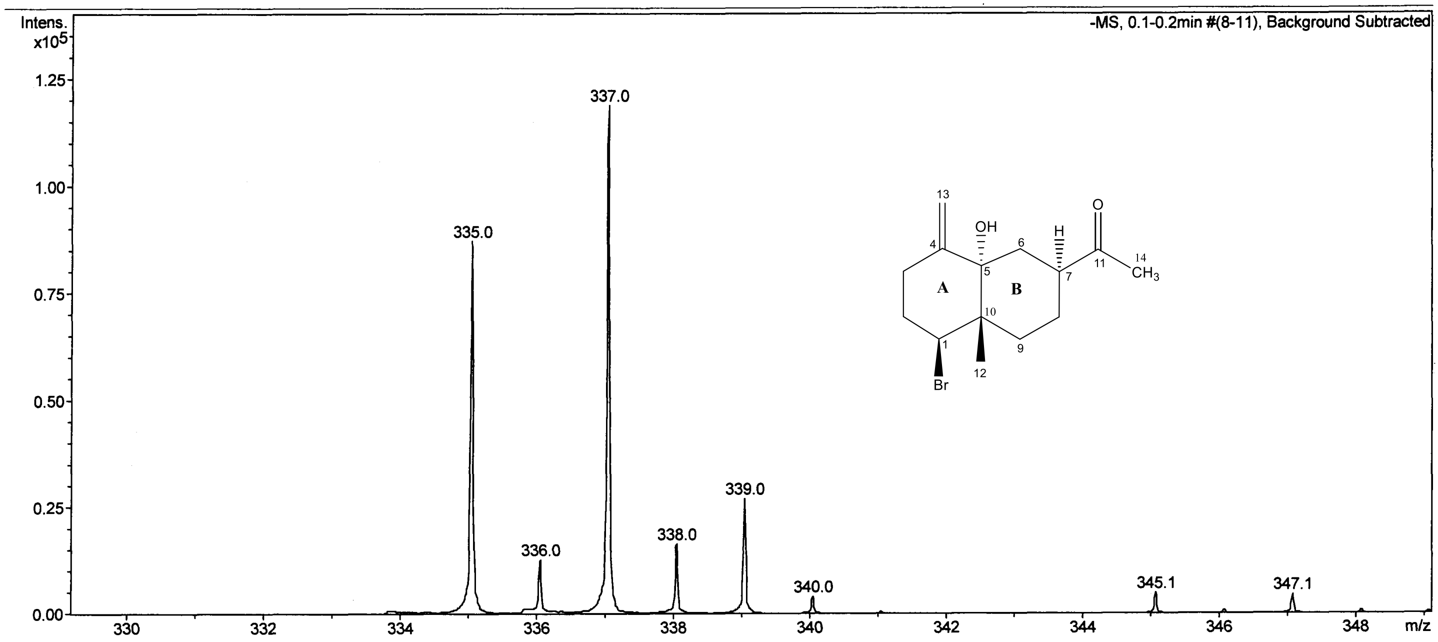

Bruker Compass DataAnalysis 4.0 printed: 25.02.2016 09:29:17 Page 1 of 1

Figure S31: (-)-ESI mass spectrum of 7-acetyl-aplysiol (**2**)

Mass Spectrum SmartFormula Report

Analysis Info

Analysis Name	Z:\Data\2016\1602\sam250216\mshaaba00065 low_1_01_86571.d	Acquisition Date	25.02.2016 10:31:18
Method	hystar_pl.m	Operator	BDAL@DE
Sample Name	mshaaba00065 low	Instrument / Ser#	micrOTOF 10237
Comment			

Acquisition Parameter

Source Type	ESI	Ion Polarity	Positive	Set Nebulizer	1.2 Bar
Focus	Not active			Set Dry Heater	180 °C
Scan Begin	50 m/z	Set Capillary	4500 V	Set Dry Gas	6.0 l/min
Scan End	1600 m/z	Set End Plate Offset	-500 V	Set Divert Valve	Source

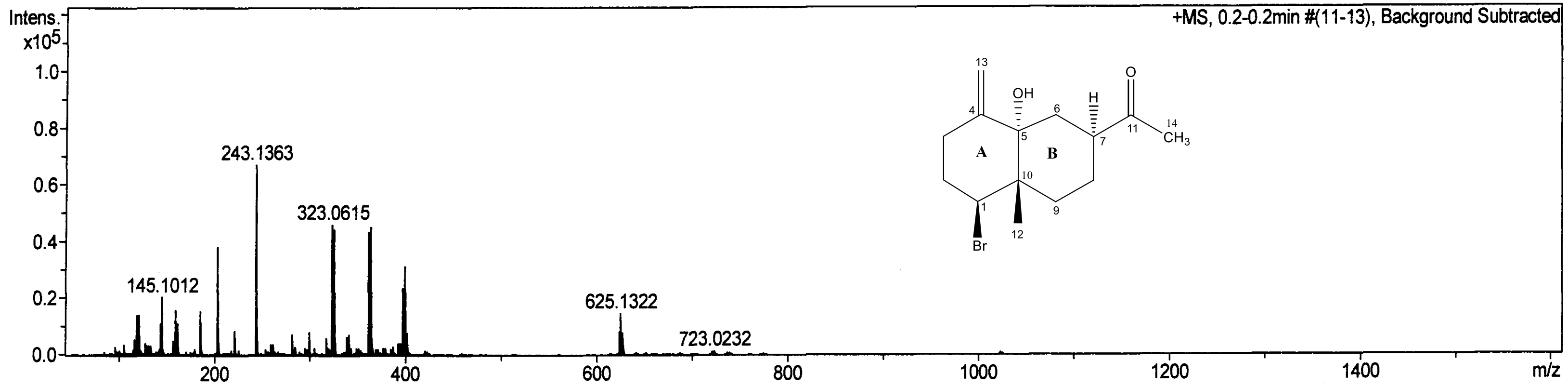

Meas. m/z	#	Formula	m/z	err [ppm]	Mean err [ppm]	rdb	N-Rule	e⁻ Conf	mSigma	Std I	Std Mean m/z	Std I VarNorm	Std m/z Diff	Std Comb Dev
323.0615														
	1	C 14 H 21 Br Na O 2	323.0617	0.7	0.9	3.5	ok	even	12.97	0.0097	0.0009	0.0038	0.0015	0.8427
	2	C 10 H 17 Br N 6 Na	323.0590	-7.6	-7.5	4.5	ok	even	14.98	0.0186	0.0026	0.0073	0.0014	0.9722

Figure S32: (+)-ESI HR mass spectrum of 7-acetyl-aplysiol (**2**)

Display Report

**Analysis Info**		Acquisition Date	25.02.2016 10:31:18
Analysis Name	Z:\Data\2016\1602\sam250216\mshaaba00065 low_1_01_86571.d		
Method	hystar_pl.m	Operator	BDAL@DE
Sample Name	mshaaba00065 low	Instrument / Ser#	micrOTOF 10237
Comment			

Acquisition Parameter

Source Type	ESI	Ion Polarity	Positive	Set Nebulizer	1.2 Bar
Focus	Not active			Set Dry Heater	180 °C
Scan Begin	50 m/z	Set Capillary	4500 V	Set Dry Gas	6.0 l/min
Scan End	1600 m/z	Set End Plate Offset	-500 V	Set Divert Valve	Source

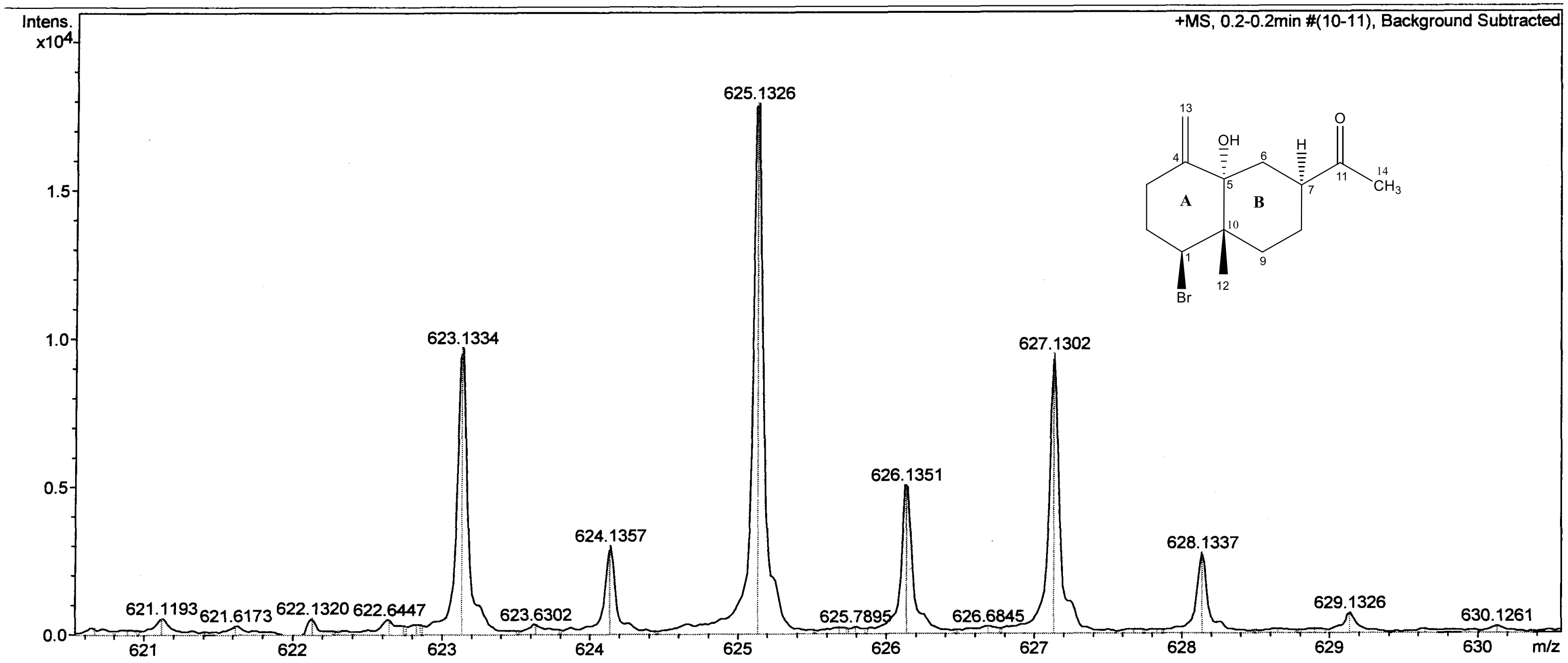

Bruker Compass DataAnalysis 4.0 printed: 25.02.2016 11:24:01 Page 1 of 1

Figure S33: (+)-ESI HR mass spectrum of 7-acetyl-aplysiol (**2**)

Mass Spectrum SmartFormula Report

Analysis Info

Analysis Name	Z:\Data\2016\1602\sam250216\neg\mshaaba00065 low_1_01_86552.d	Acquisition Date	25.02.2016 07:54:04
Method	hystar_nl.m	Operator	BDAL@DE
Sample Name	mshaaba00065 low	Instrument / Ser#	micrOTOF 10237
Comment			

Acquisition Parameter

Source Type	ESI	Ion Polarity	Negative	Set Nebulizer	1.6 Bar
Focus	Not active			Set Dry Heater	180 °C
Scan Begin	50 m/z	Set Capillary	3800 V	Set Dry Gas	8.0 l/min
Scan End	1600 m/z	Set End Plate Offset	-500 V	Set Divert Valve	Source

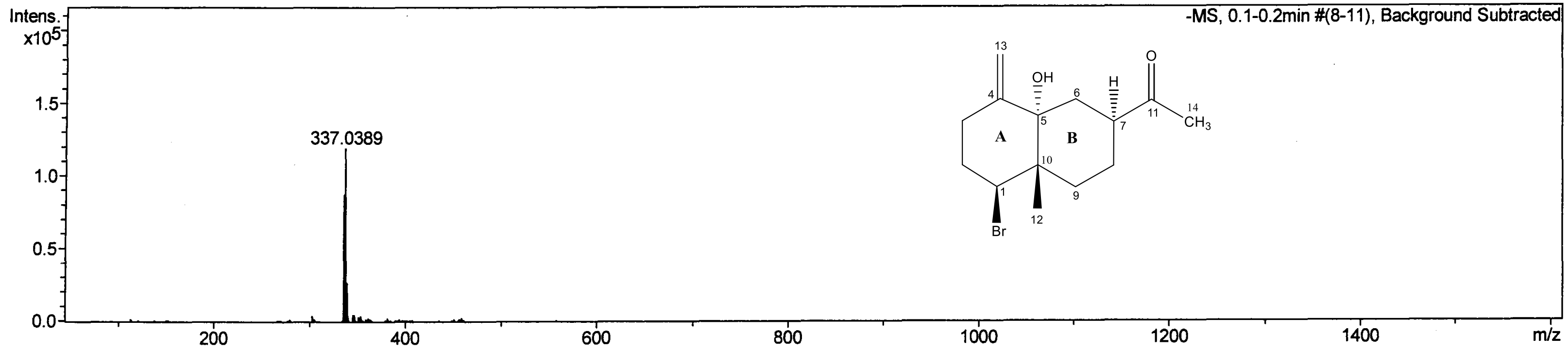

Meas. m/z	#	Formula	m/z	err [ppm]	Mean err [ppm]	rdb	N-Rule	e⁻ Conf	mSigma	Std I	Std Mean m/z	Std I VarNorm	Std m/z Diff	Std Comb Dev
335.0418														
	1	C 10 H 17 Br Cl N 6	335.0392	-7.7	-5.8	4.5	ok	even	17.07	0.0156	0.0020	0.0049	0.0008	0.9224
	2	C 14 H 21 Br Cl O 2	335.0419	0.4	2.2	3.5	ok	even	17.40	0.0175	0.0009	0.0051	0.0008	0.8452

Figure S34: (-)-ESI HR mass spectrum of 7-acetyl-aplysiol (**2**)

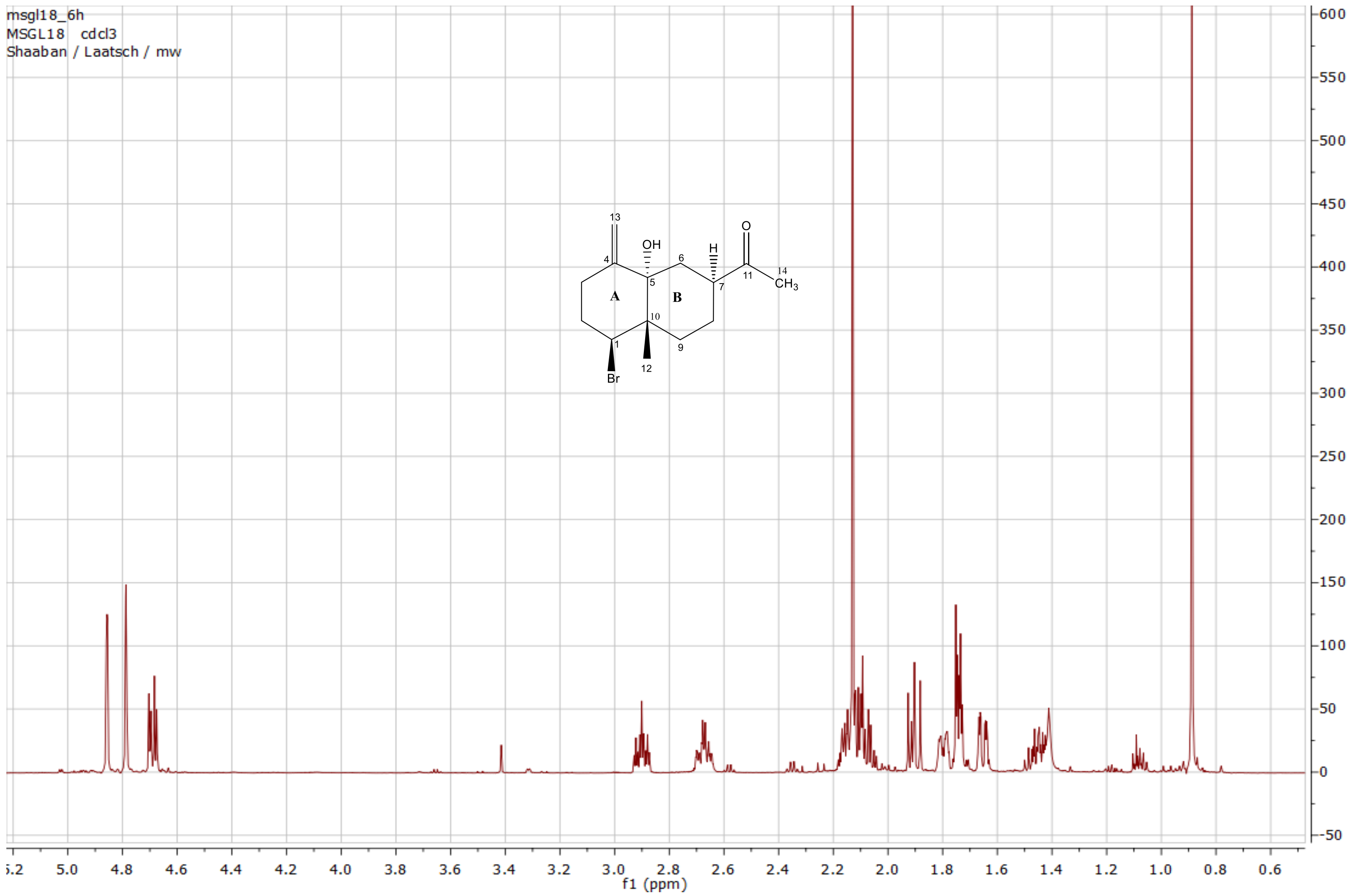

Figure S35: ^{1}H NMR spectrum (600 MHz, $CDCl_3$) of 7-acetyl-aplysiol (**2**)

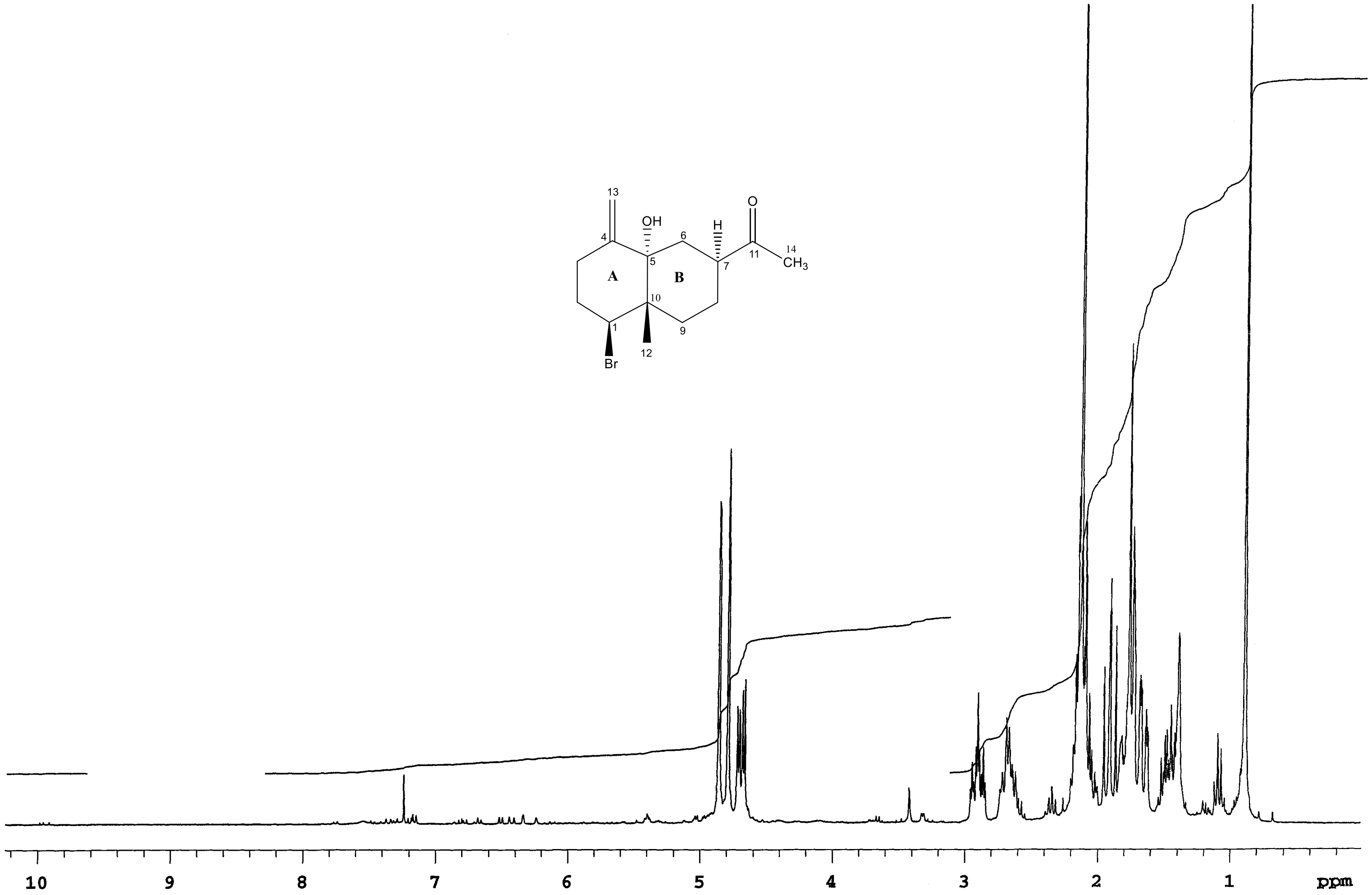

Figure S36: ^{1}H NMR spectrum (300 MHz, $CDCl_3$) of 7-acetyl-aplysiol (**2**)

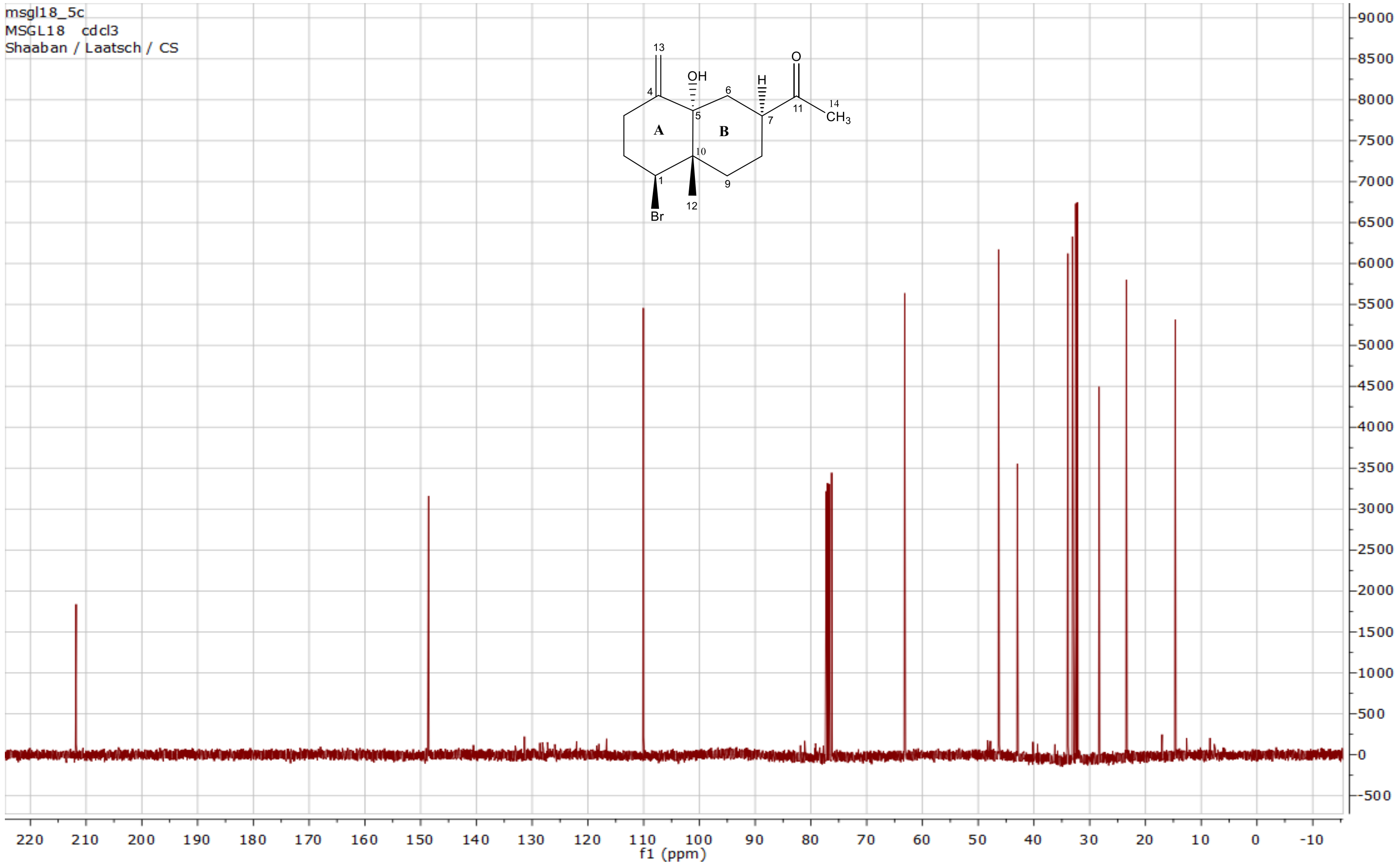

Figure S37: ^{13}C NMR spectrum (125 MHz, $CDCl_3$) of 7-acetyl-aplysiol (**2**)

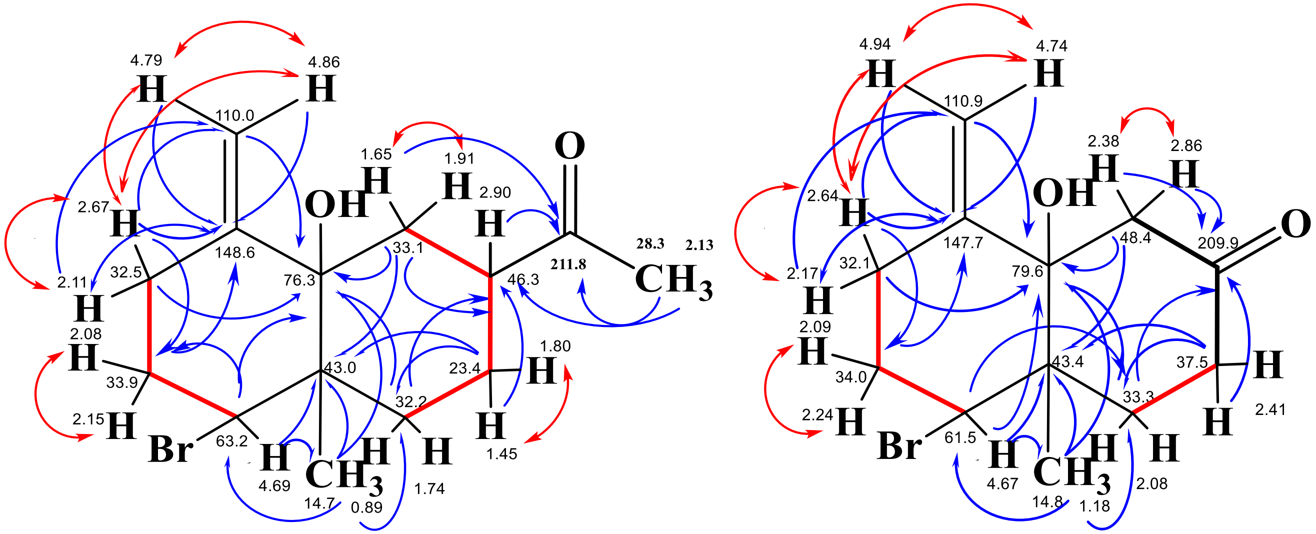

Figure S38: H,H COSY (^3J ▬, ^2J,^4J ↶) and HMBC (↷) correlations of 7-acetyl-aplysiol (**2**) and aplysiol-7-one (**3**)

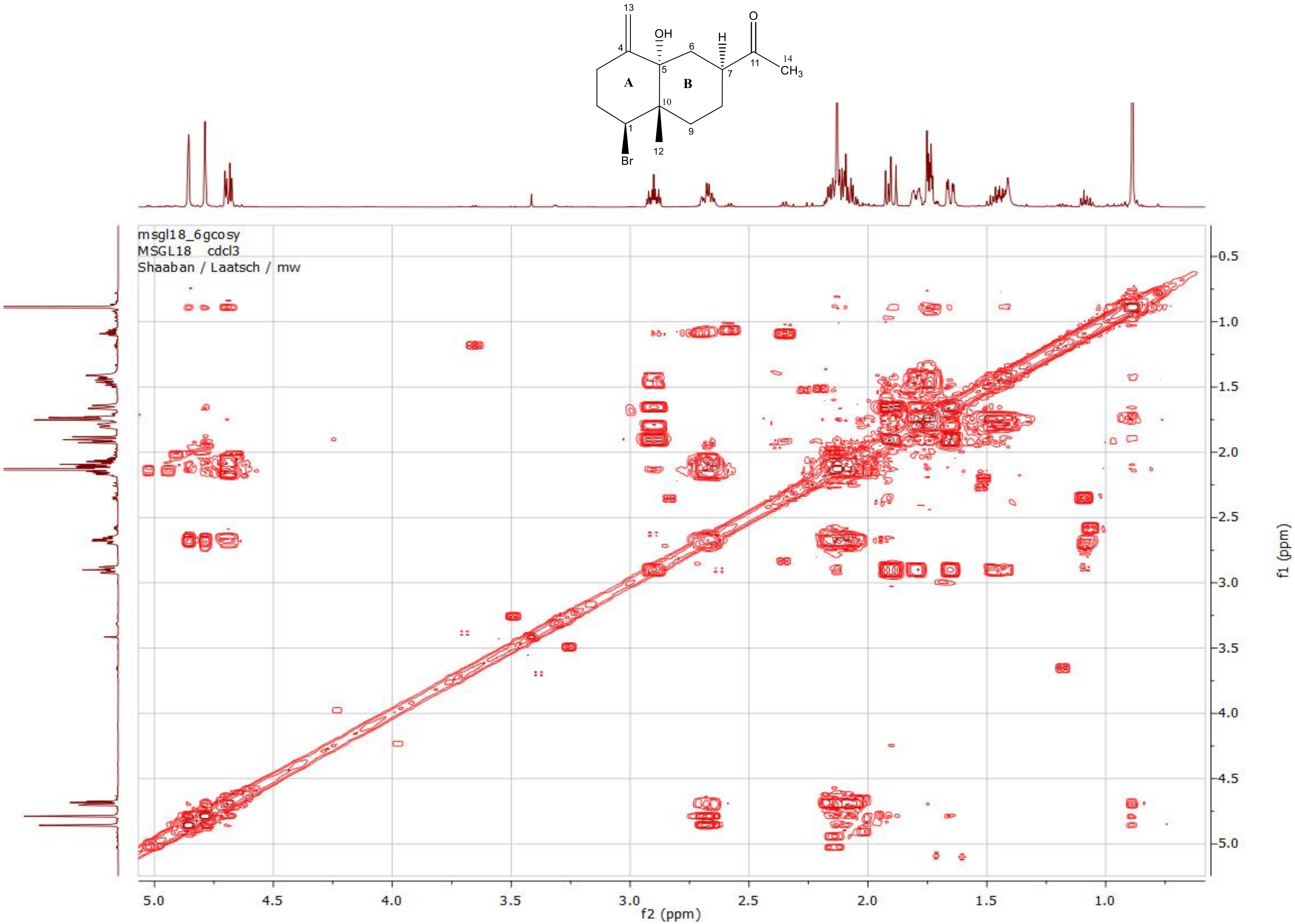

Figure S39: H,H COSY spectrum (600 MHz, $CDCl_3$) of 7-acetyl-aplysiol (**2**)

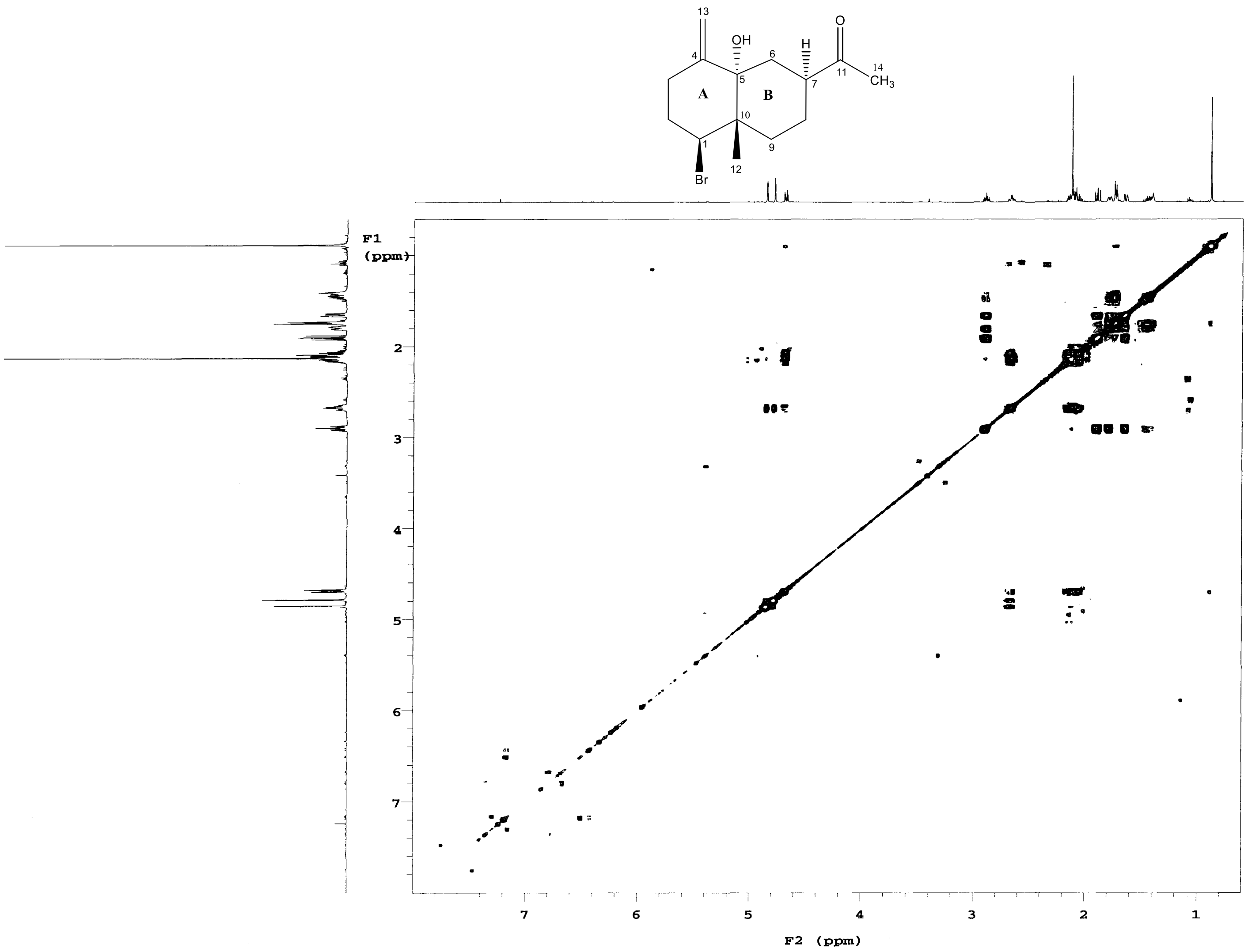

Figure S40: H,H COSY spectrum (600 MHz, $CDCl_3$) of 7-acetyl-aplysiol (**2**)

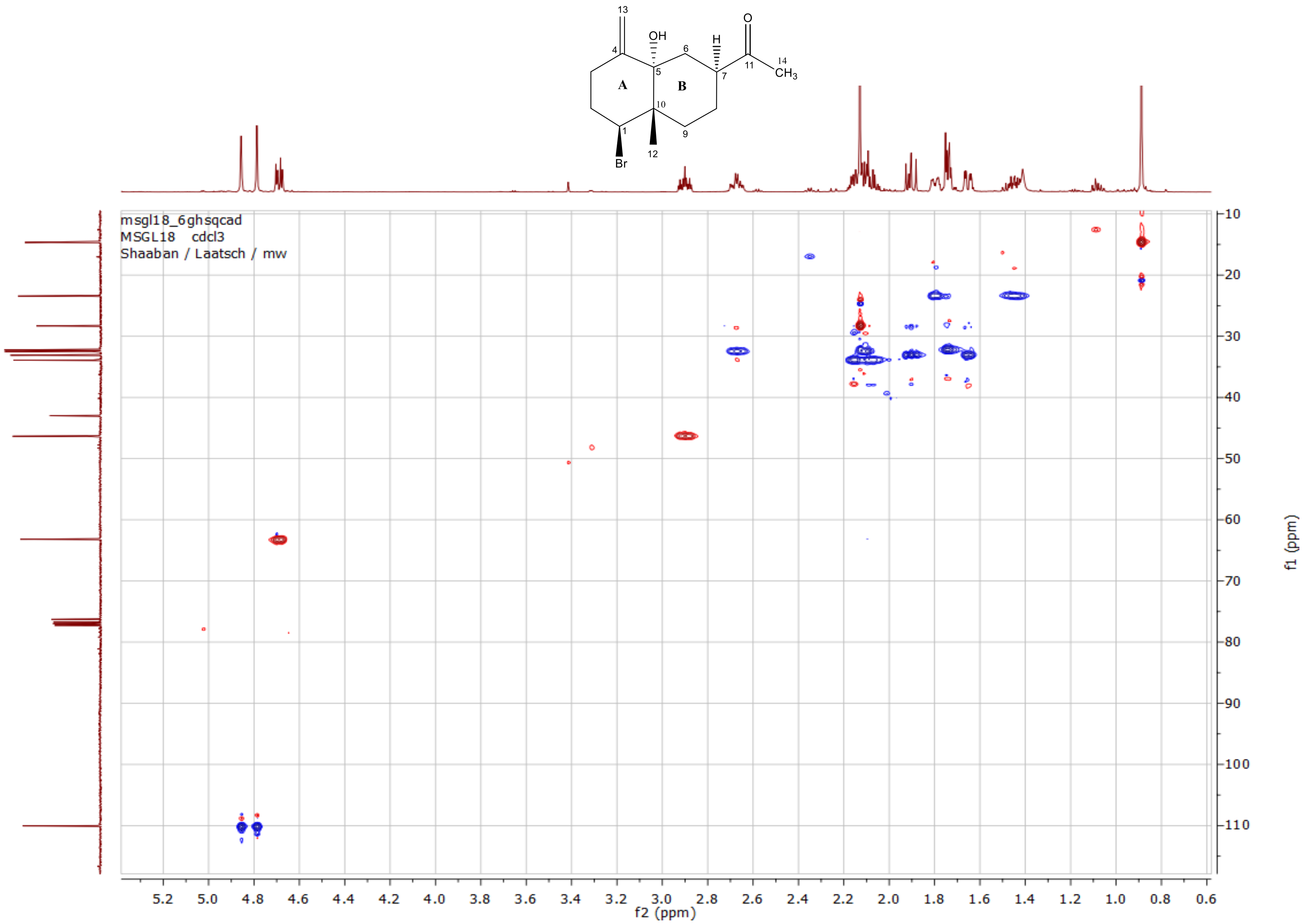

Figure S41: HMQC spectrum (600 MHz, $CDCl_3$) of 7-acetyl-aplysiol (**2**)

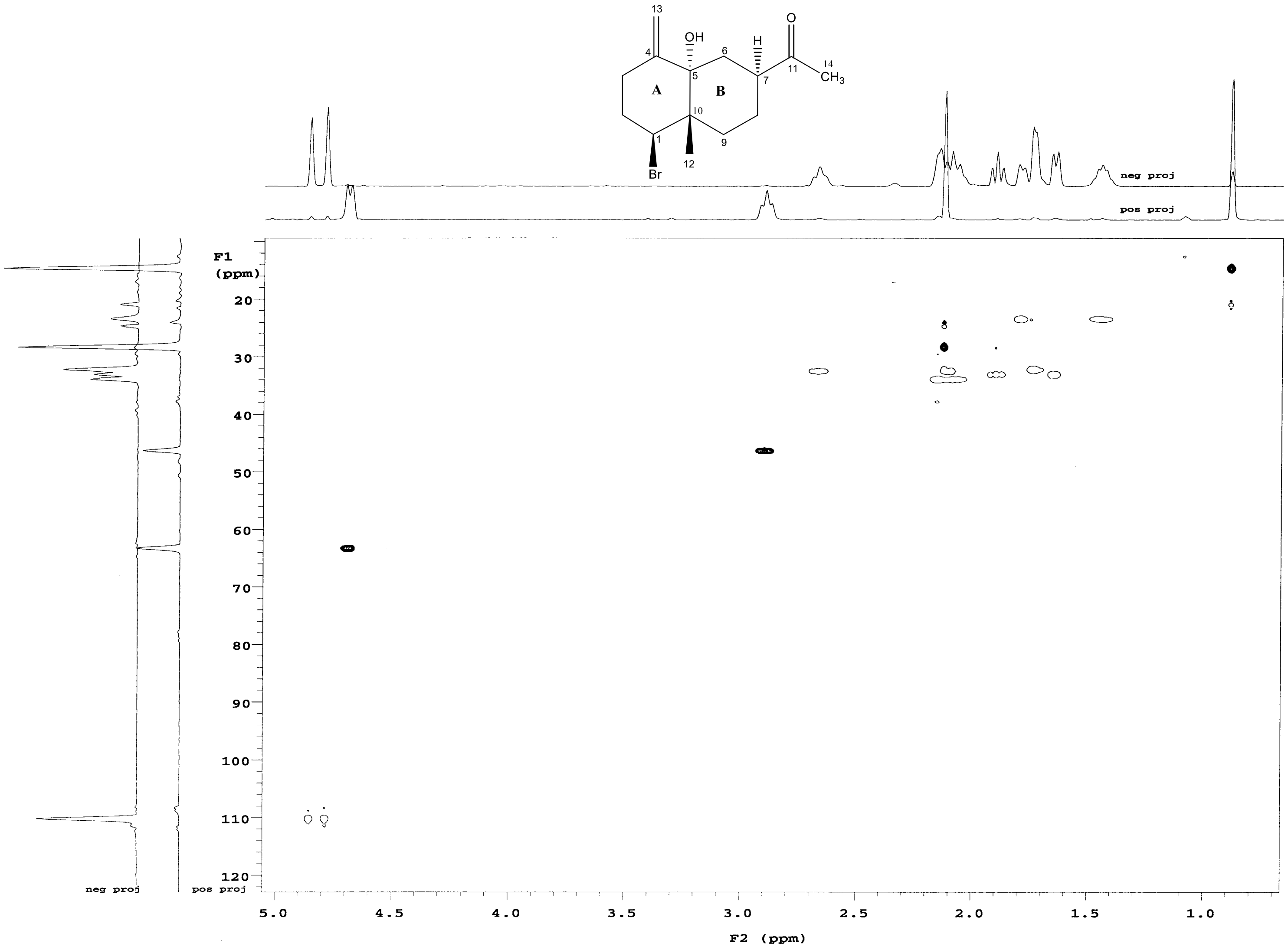

Figure S42: HSQC spectrum (600 MHz, $CDCl_3$) of 7-acetyl-aplysiol (**2**)

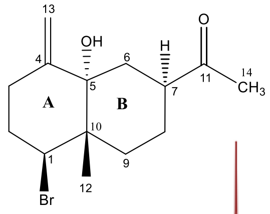

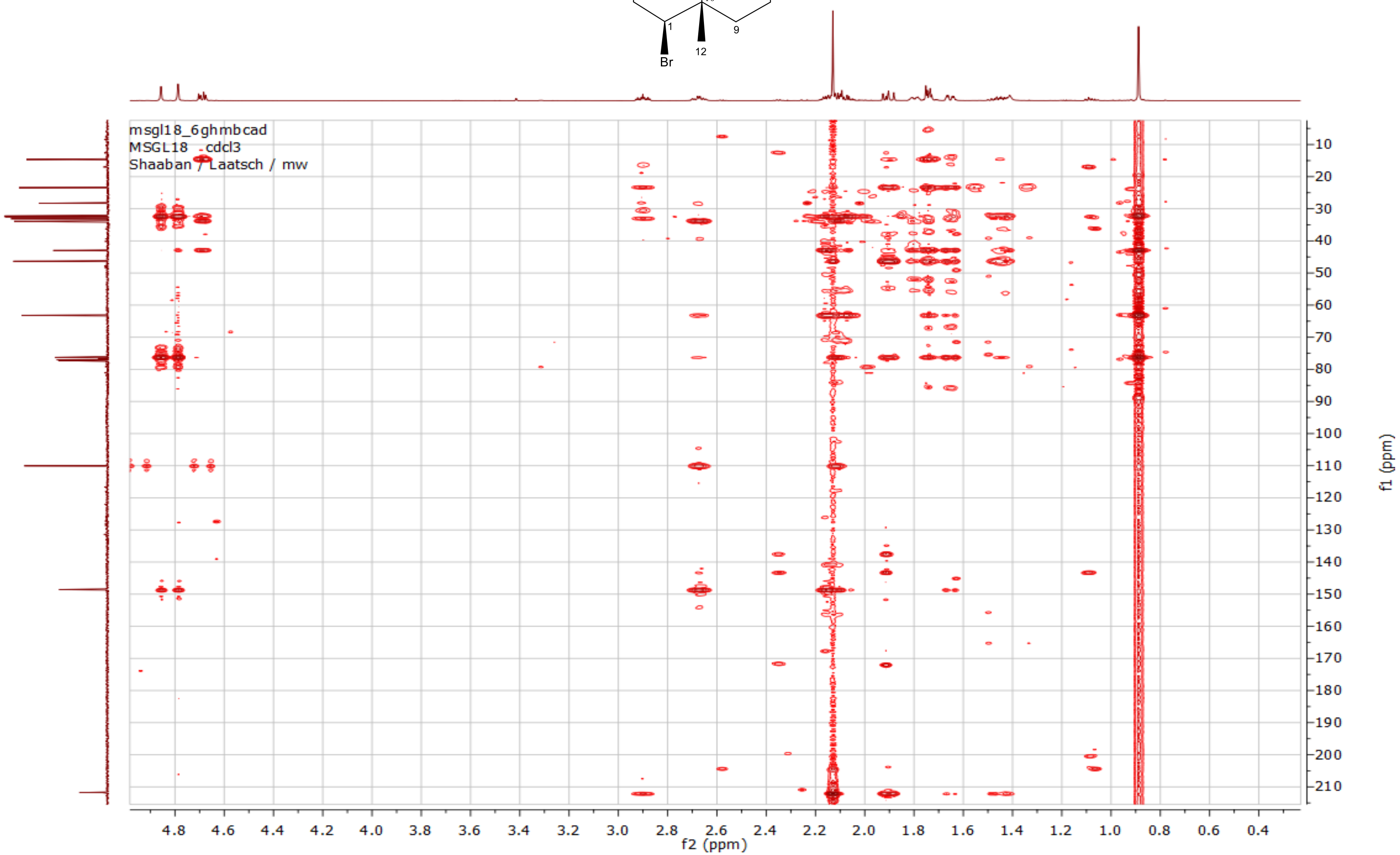

Figure S43: HMBC spectrum (600 MHz, $CDCl_3$) of 7-acetyl-aplysiol (**2**)

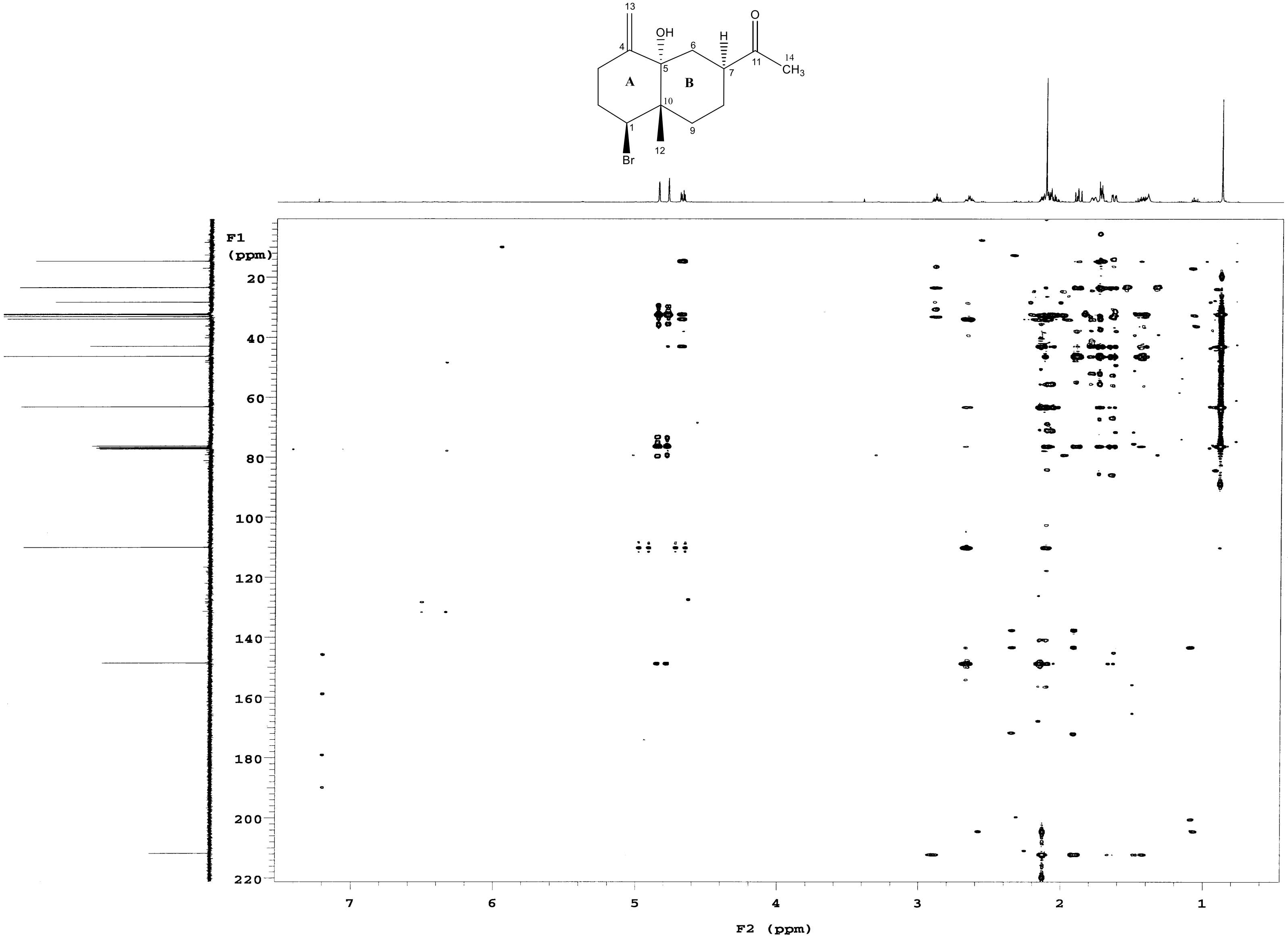

Figure S44: HMBC spectrum (600 MHz, $CDCl_3$) of 7-acetyl-aplysiol (**2**)

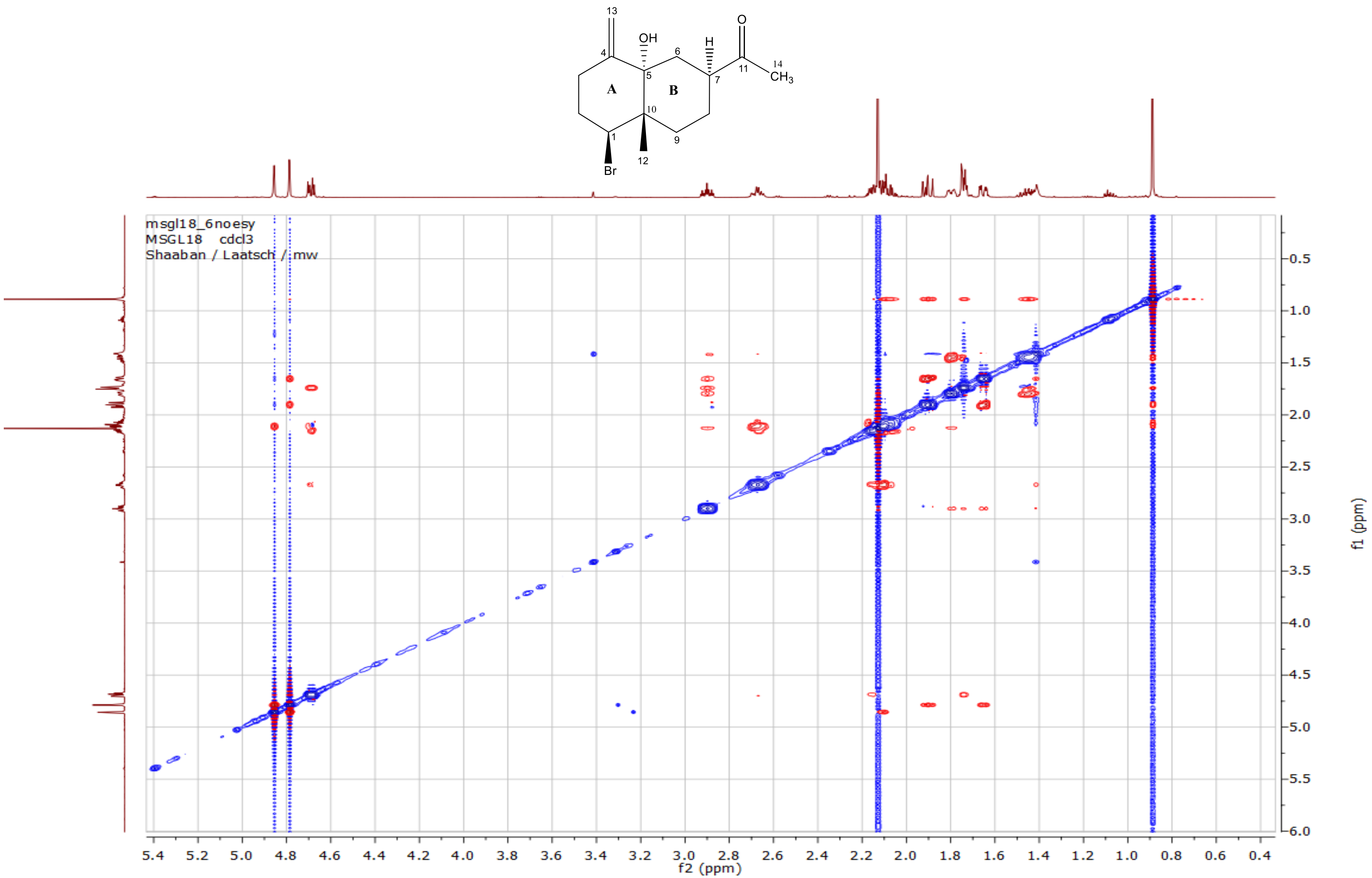

Figure S45: NOESY spectrum (600 MHz, $CDCl_3$) of 7-acetyl-aplysiol (**2**)

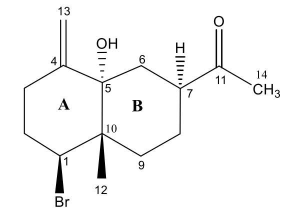

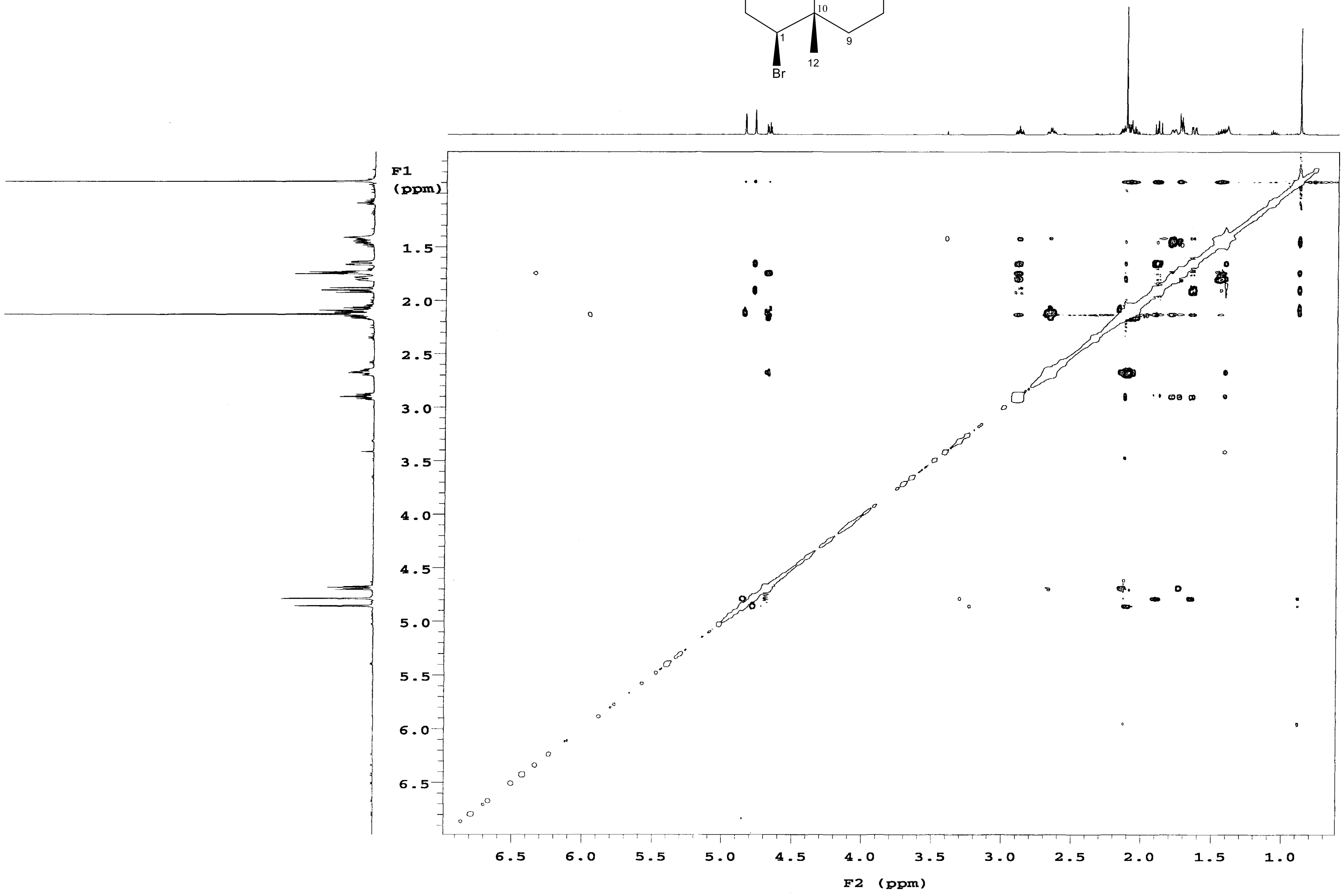

Figure S46: NOESY spectrum (600 MHz, $CDCl_3$) of 7-acetyl-aplysiol (**2**)

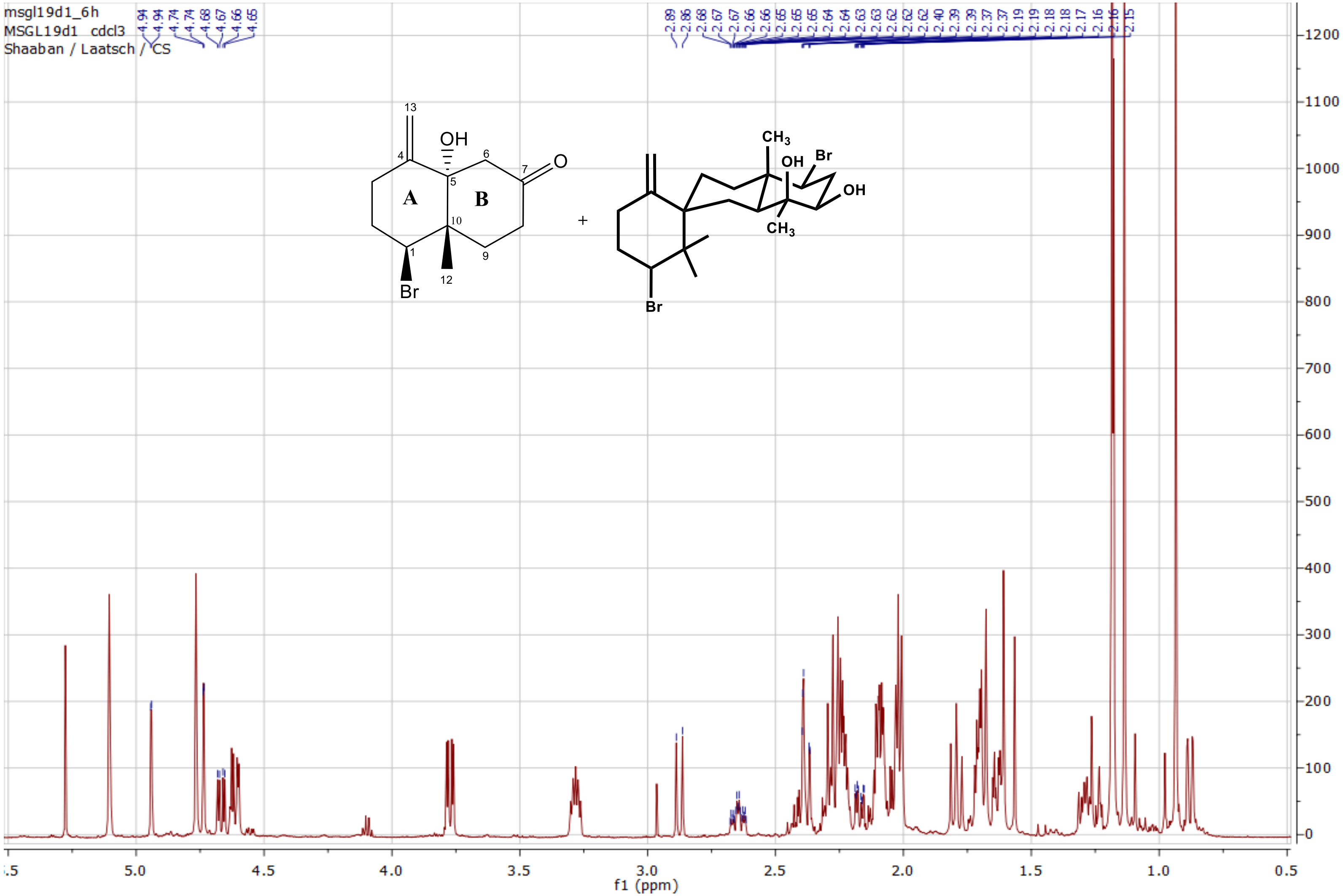

Figure S47: ^{1}H NMR spectrum (600 MHz, $CDCl_3$) of aplysiol-7-one (**3**) and 10-hydroxykahukuene B

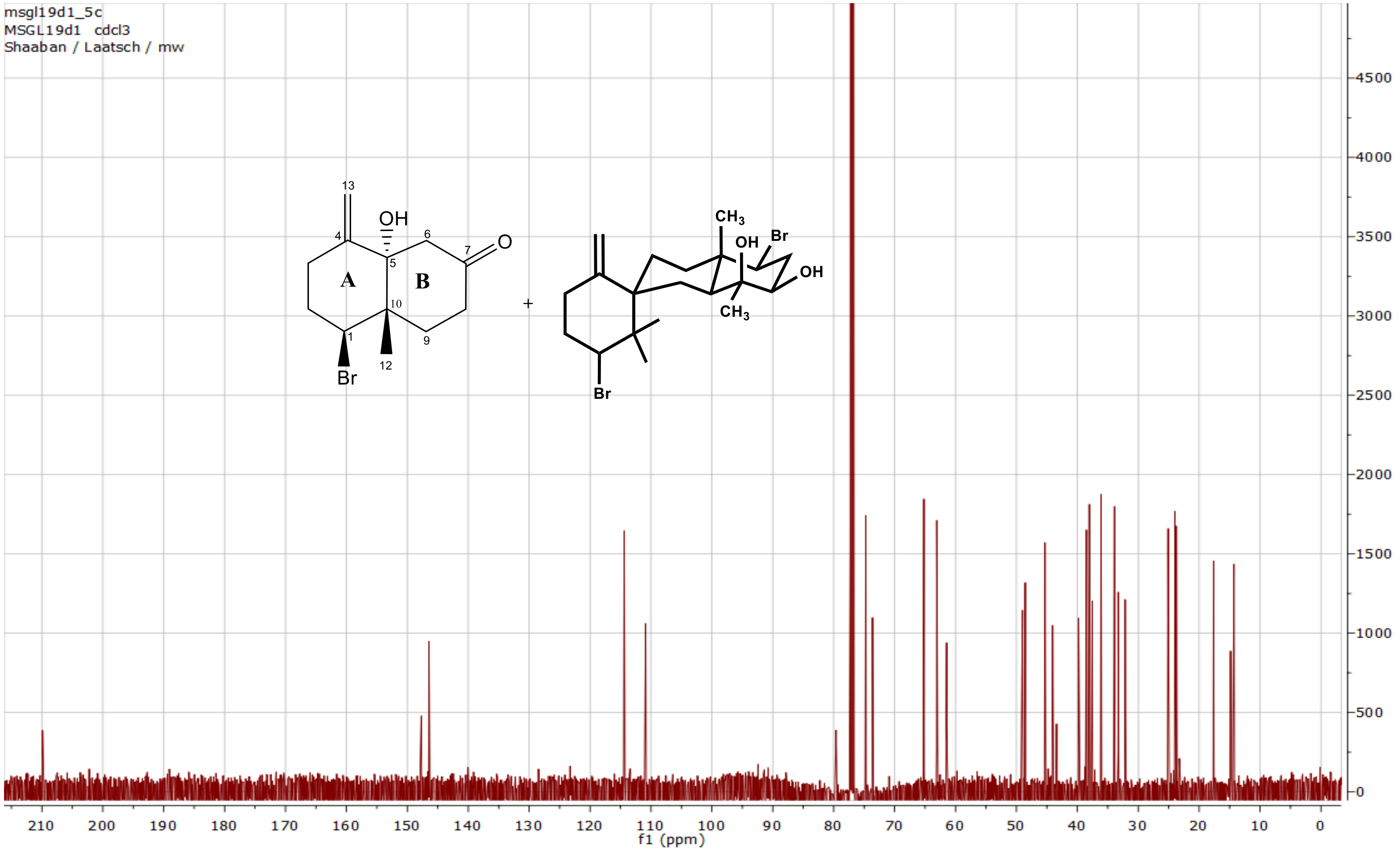

Figure S48: ^{13}C NMR spectrum (125 MHz, $CDCl_3$) of aplysiol-7-one (**3**) and 10-hydroxykahukuene B

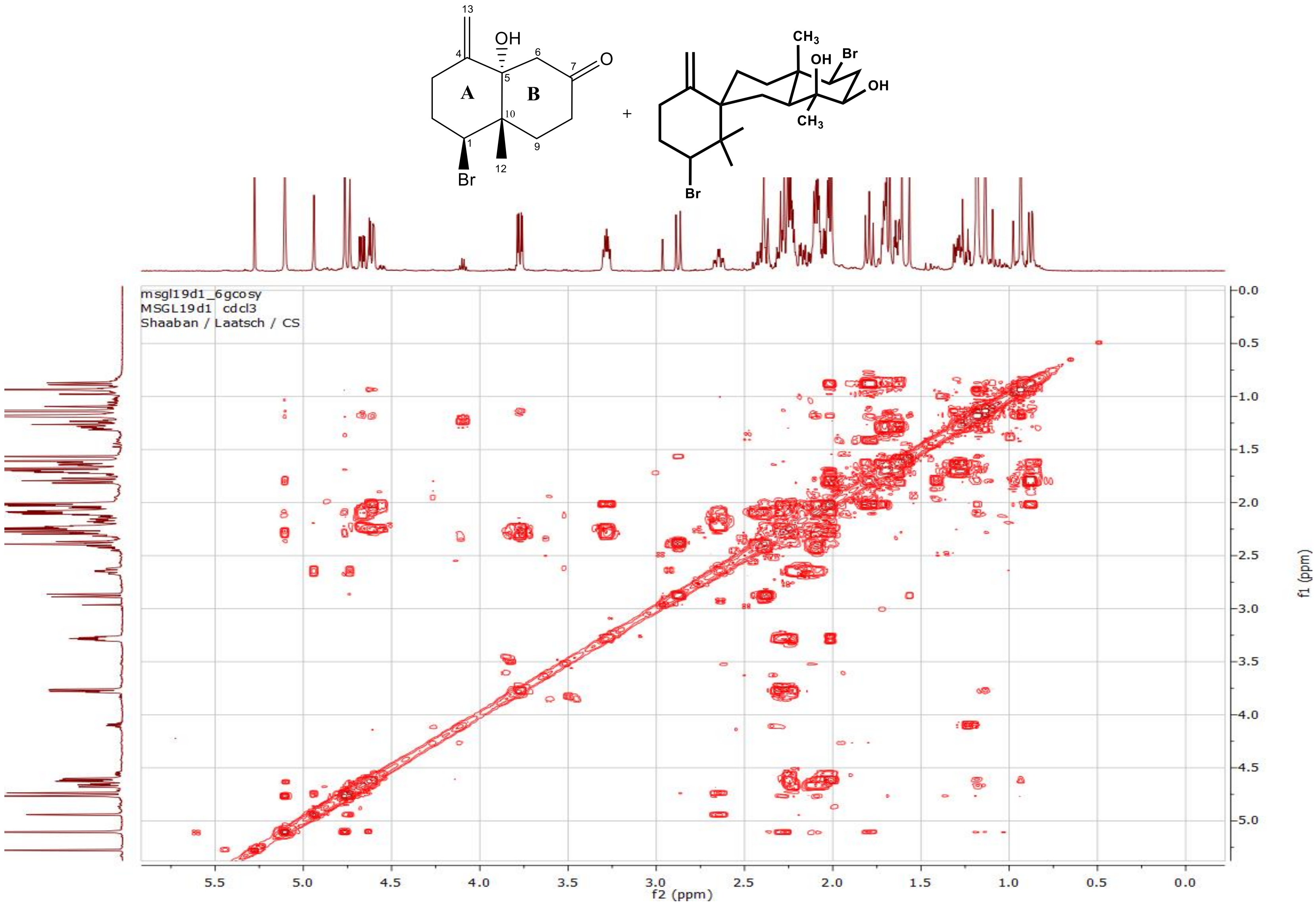

Figure S49: H,H COSY spectrum (600 MHz, $CDCl_3$) of aplysiol-7-one (**3**) and 10-hydroxykahukuene B

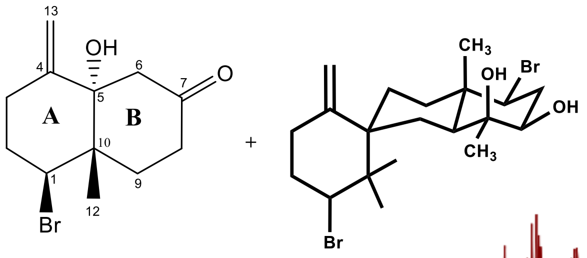

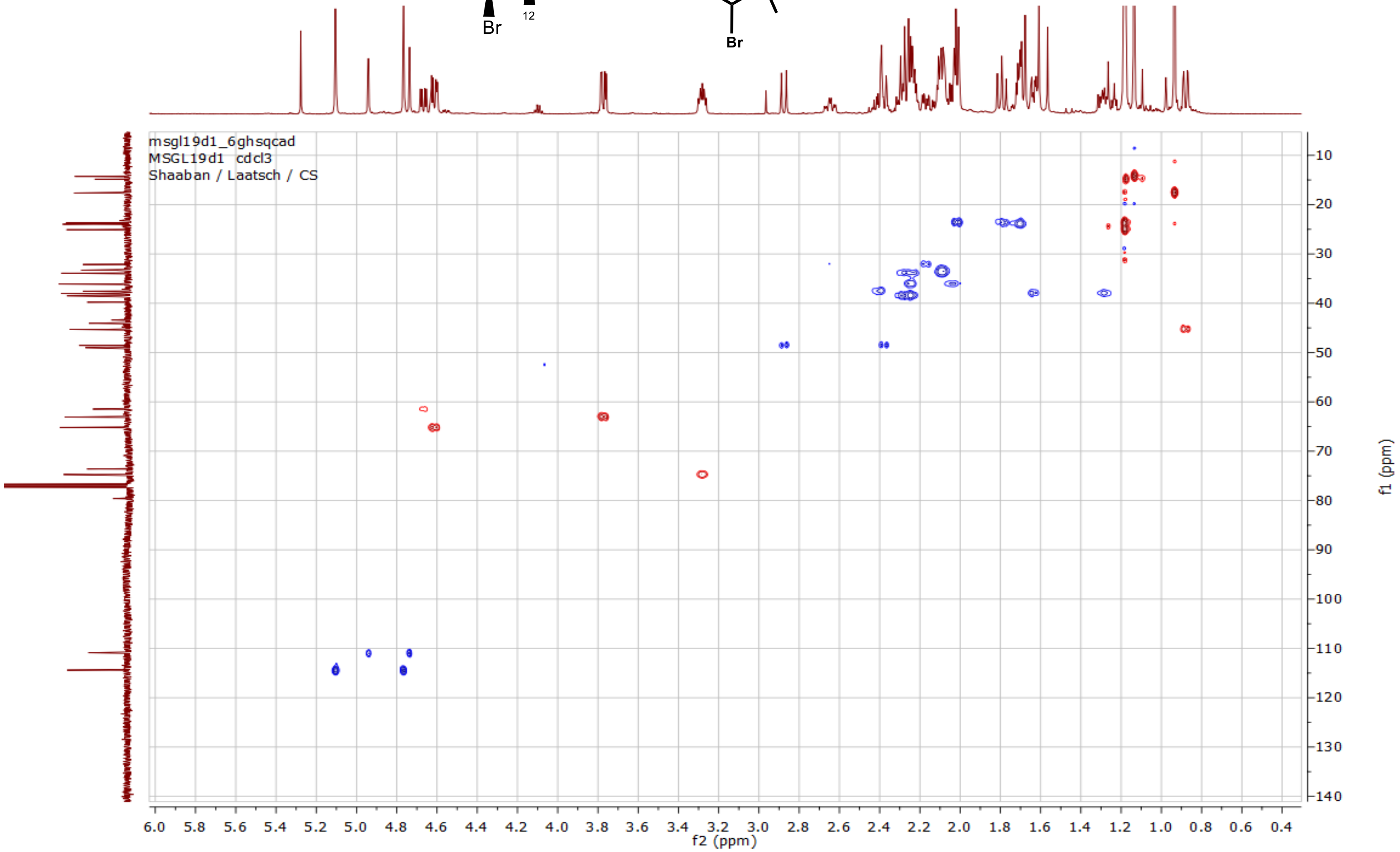

Figure S50: HMQC spectrum (600 MHz, $CDCl_3$) of aplysiol-7-one (**3**) and 10-hydroxykahukuene B

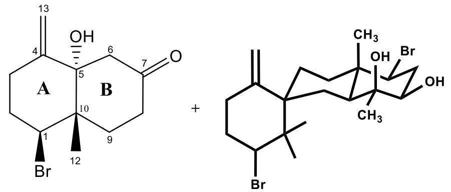

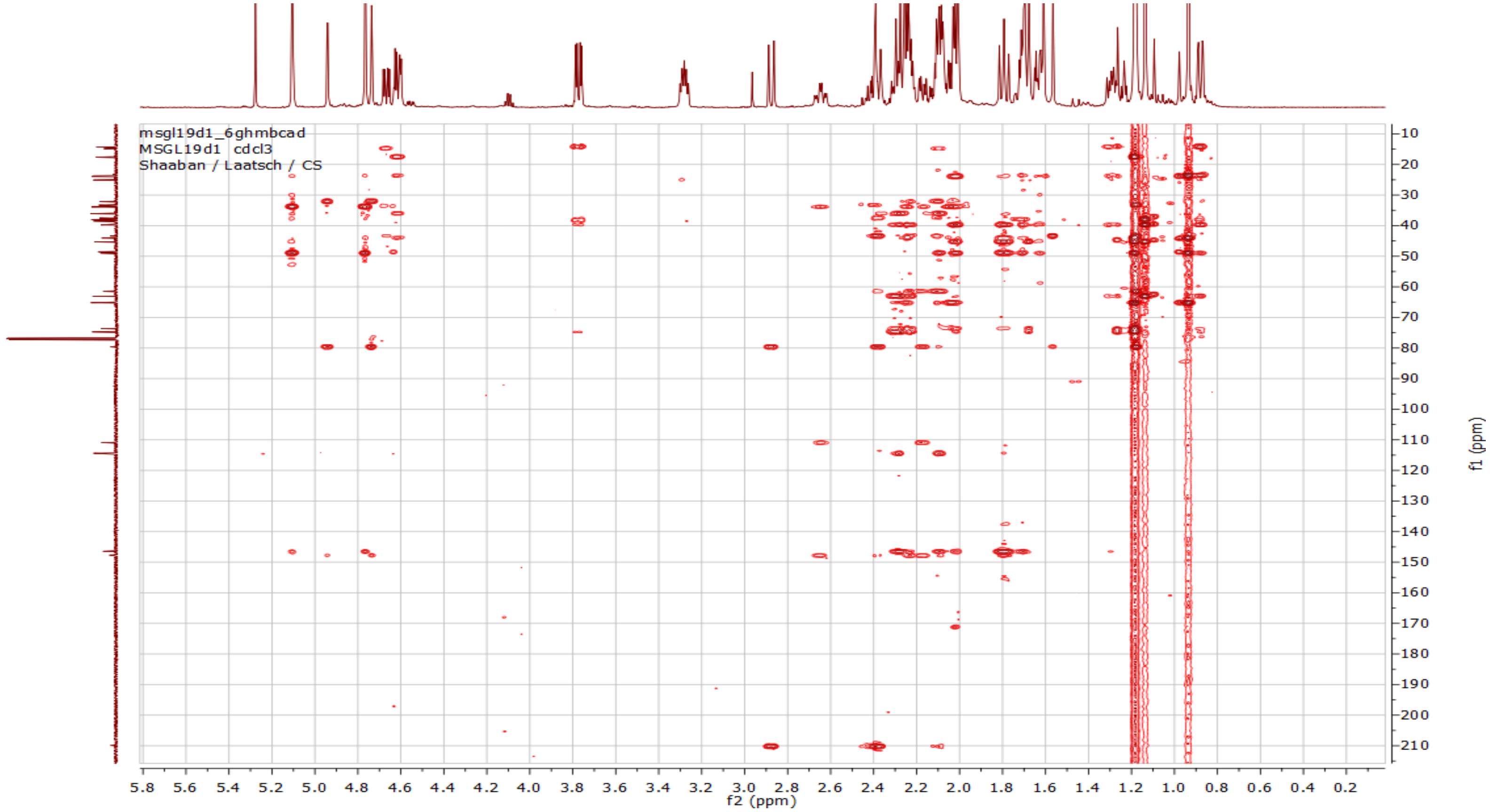

Figure S51: HMBC spectrum (600 MHz, $CDCl_3$) of aplysiol-7-one (**3**) and 10-hydroxykahukuene B

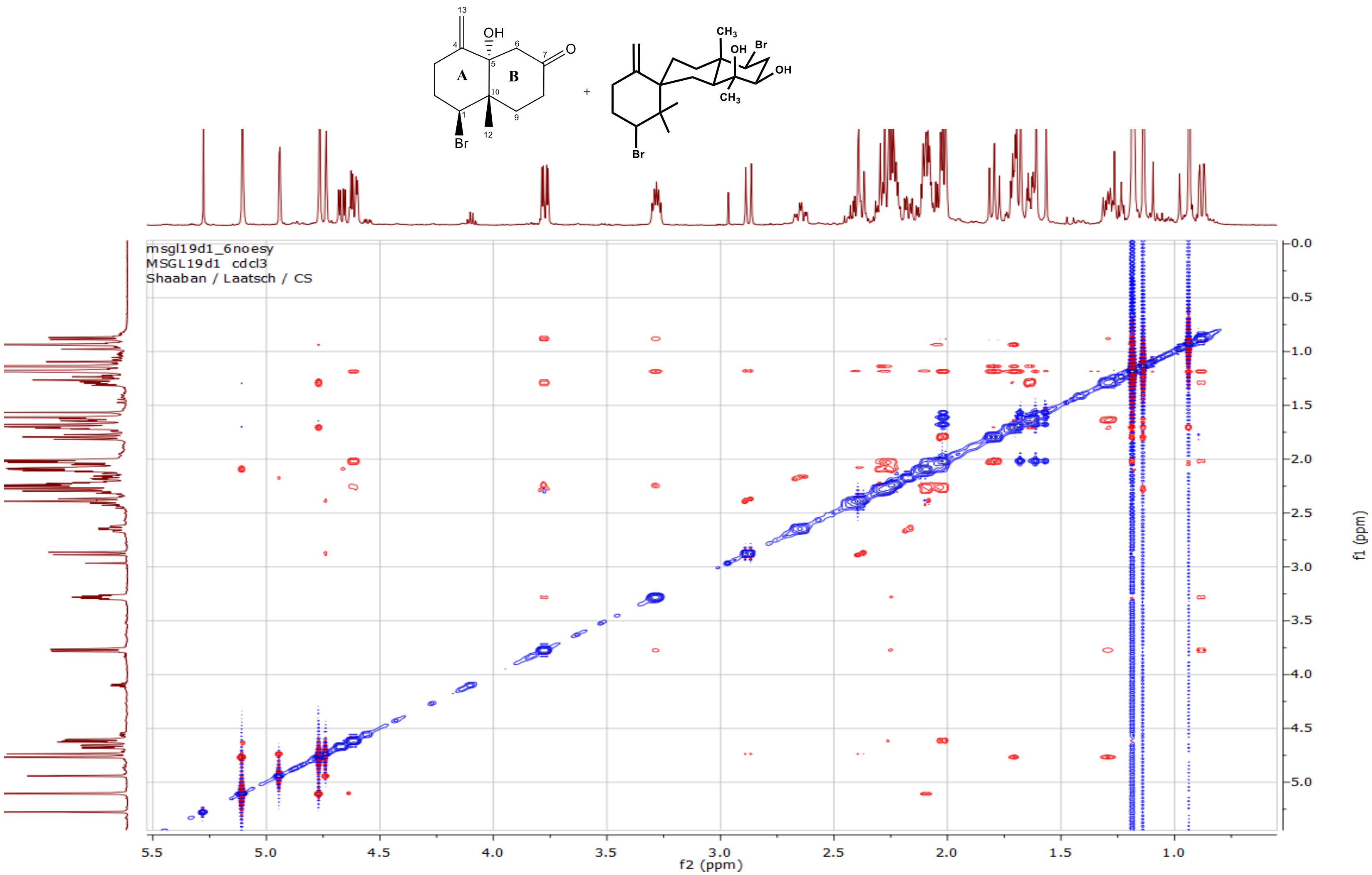

Figure S52: NOESY spectrum (600 MHz, $CDCl_3$) of aplysiol-7-one (**3**) and 10-hydroxykahukuene B

Display Report

Analysis Info

Analysis Name	Z:\Data\2016\1602\sam040216\mshaaba00038 low_2_01_85866.d	Acquisition Date	04.02.2016 10:21:26
Method	hystar_pl.m	Operator	BDAL@DE
Sample Name	mshaaba00038 low	Instrument / Ser#	micrOTOF 10237
Comment			

Acquisition Parameter

Source Type	ESI	Ion Polarity	Positive	Set Nebulizer	1.2 Bar
Focus	Not active			Set Dry Heater	180 °C
Scan Begin	50 m/z	Set Capillary	4500 V	Set Dry Gas	6.0 l/min
Scan End	1600 m/z	Set End Plate Offset	-500 V	Set Divert Valve	Source

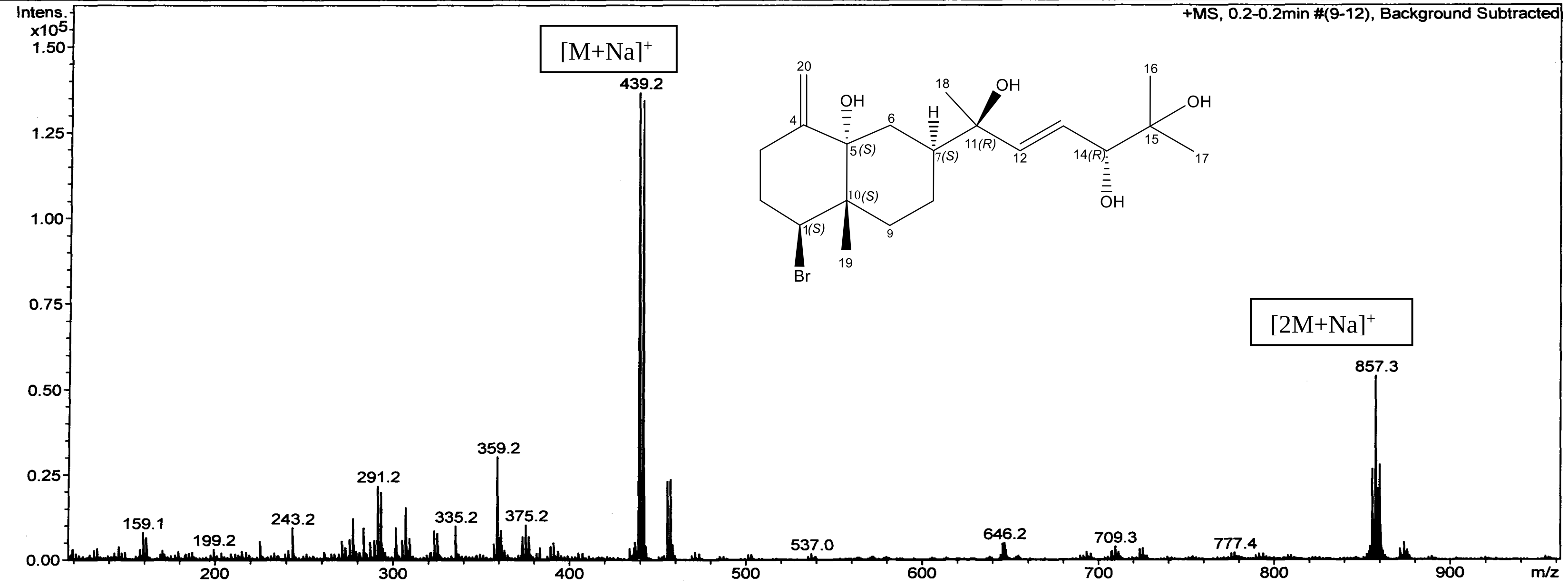

Figure S53: (+)-ESI mass spectrum of dihydroaplysia-5,11,14,15-tetrol (**5**)

Display Report

Analysis Info

Analysis Name	Z:\Data\2016\1602\sam040216\mshaaba00038 low_2_01_85866.d	Acquisition Date	04.02.2016 10:21:26
Method	hystar_pl.m	Operator	BDAL@DE
Sample Name	mshaaba00038 low	Instrument / Ser#	micrOTOF 10237
Comment			

Acquisition Parameter

Source Type	ESI	Ion Polarity	Positive	Set Nebulizer	1.2 Bar
Focus	Not active			Set Dry Heater	180 °C
Scan Begin	50 m/z	Set Capillary	4500 V	Set Dry Gas	6.0 l/min
Scan End	1600 m/z	Set End Plate Offset	-500 V	Set Divert Valve	Source

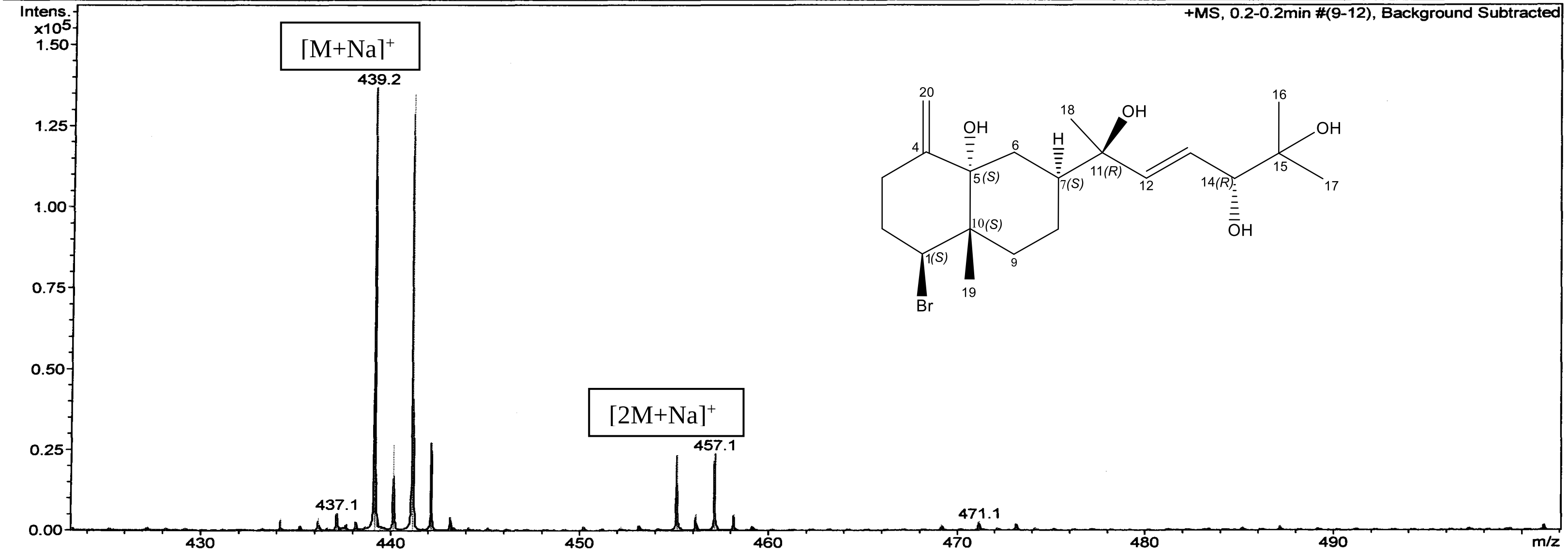

Bruker Compass DataAnalysis 4.0 printed: 04.02.2016 11:08:16 Page 1 of 1

Figure S54: (+)-ESI mass spectrum of dihydroaplysia-5,11,14,15-tetrol (**5**)

Display Report

Analysis Info

Analysis Name	Z:\Data\2016\1602\sam040216\neg\mshaaba00038 low_2_01_85851.d	Acquisition Date	04.02.2016 08:01:49
Method	hystar_nl.m	Operator	BDAL@DE
Sample Name	mshaaba00038 low	Instrument / Ser#	micrOTOF 10237
Comment			

Acquisition Parameter

Source Type	ESI	Ion Polarity	Negative	Set Nebulizer	1.6 Bar
Focus	Not active			Set Dry Heater	180 °C
Scan Begin	50 m/z	Set Capillary	3800 V	Set Dry Gas	8.0 l/min
Scan End	1600 m/z	Set End Plate Offset	-500 V	Set Divert Valve	Source

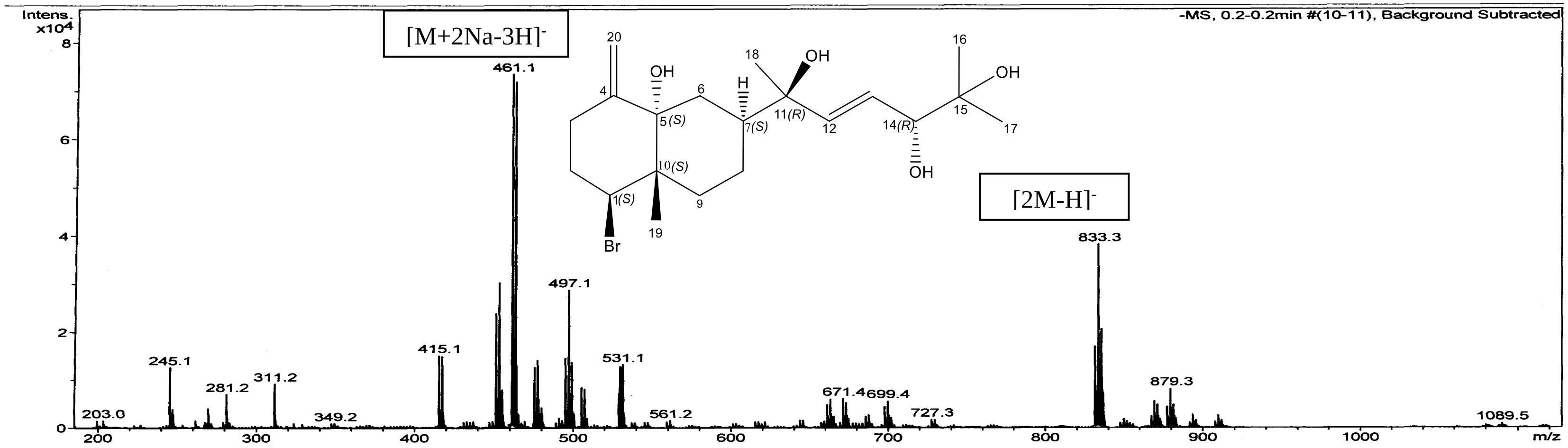

Bruker Compass DataAnalysis 4.0 printed: 04.02.2016 09:25:26 Page 1 of 1

Figure S55: (-)-ESI mass spectrum of dihydroaplysia-5,11,14,15-tetrol (**5**)

Mass Spectrum SmartFormula Report

Analysis Info

Analysis Name	Z:\Data\2016\1602\sam040216\mshaaba00038 low_2_01_85866.d	Acquisition Date	04.02.2016 10:21:26
Method	hystar_pl.m	Operator	BDAL@DE
Sample Name	mshaaba00038 low	Instrument / Ser#	micrOTOF 10237
Comment			

Acquisition Parameter

Source Type	ESI	Ion Polarity	Positive	Set Nebulizer	1.2 Bar
Focus	Not active			Set Dry Heater	180 °C
Scan Begin	50 m/z	Set Capillary	4500 V	Set Dry Gas	6.0 l/min
Scan End	1600 m/z	Set End Plate Offset	-500 V	Set Divert Valve	Source

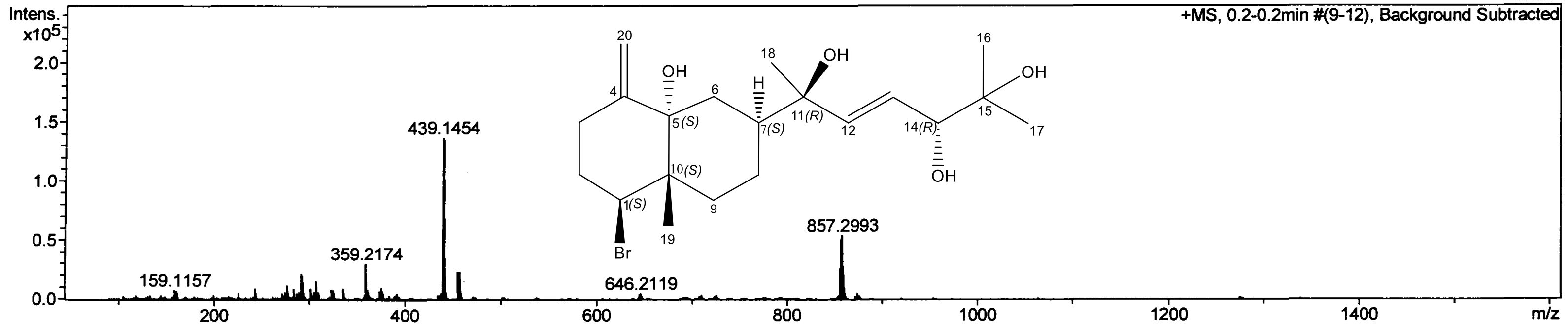

Meas. m/z	#	Formula	m/z	err [ppm]	Mean err [ppm]	rdb	N-Rule	e⁻ Conf	mSigma	Std I	Std Mean m/z	Std I VarNorm	Std m/z Diff	Std Comb Dev
439.1454														
	1	C 16 H 29 Br N 6 Na O 2	439.1428	-6.0	-5.4	4.5	ok	even	6.89	0.0068	0.0026	0.0032	0.0021	0.4849
	2	C 20 H 33 Br Na O 4	439.1454	0.1	0.7	3.5	ok	even	15.60	0.0152	0.0011	0.0054	0.0021	0.3696
	3	C 21 H 29 Br N 4 Na	439.1468	3.1	3.8	8.5	ok	even	28.26	0.0278	0.0020	0.0100	0.0021	0.4768
	4	C 33 H 20 Na	439.1457	0.7	11.1	23.5	ok	even	478.49	0.5013	0.0065	0.2294	0.0067	0.8868
	5	C 28 H 20 N 2 Na O 2	439.1417	-8.4	2.0	19.5	ok	even	480.60	0.5165	0.0044	0.2383	0.0067	0.8489
	6	C 15 H 28 Na O 13	439.1422	-7.3	0.4	1.5	ok	even	482.57	0.5648	0.0032	0.2469	0.0047	0.8178
	7	C 21 H 24 N 2 Na O 7	439.1476	4.9	13.9	10.5	ok	even	545.68	0.5399	0.0073	0.3532	0.0025	0.9278
	8	C 22 H 20 N 6 Na O 3	439.1489	8.0	16.9	15.5	ok	even	547.78	0.5364	0.0084	0.3578	0.0024	0.9393
	9	C 16 H 24 N 4 Na O 9	439.1435	-4.2	2.2	6.5	ok	even	548.35	0.5587	0.0029	0.3560	0.0024	0.8545
	10	C 17 H 20 N 8 Na O 5	439.1449	-1.2	7.7	11.5	ok	even	550.48	0.5544	0.0052	0.3630	0.0024	0.9038
	11	C 18 H 16 N 12 Na O	439.1462	1.9	10.8	16.5	ok	even	553.93	0.5528	0.0062	0.3722	0.0023	0.9190

Figure S56: (+)-ESI HR mass spectrum of dihydroaplysia-5,11,14,15-tetrol (**5**)

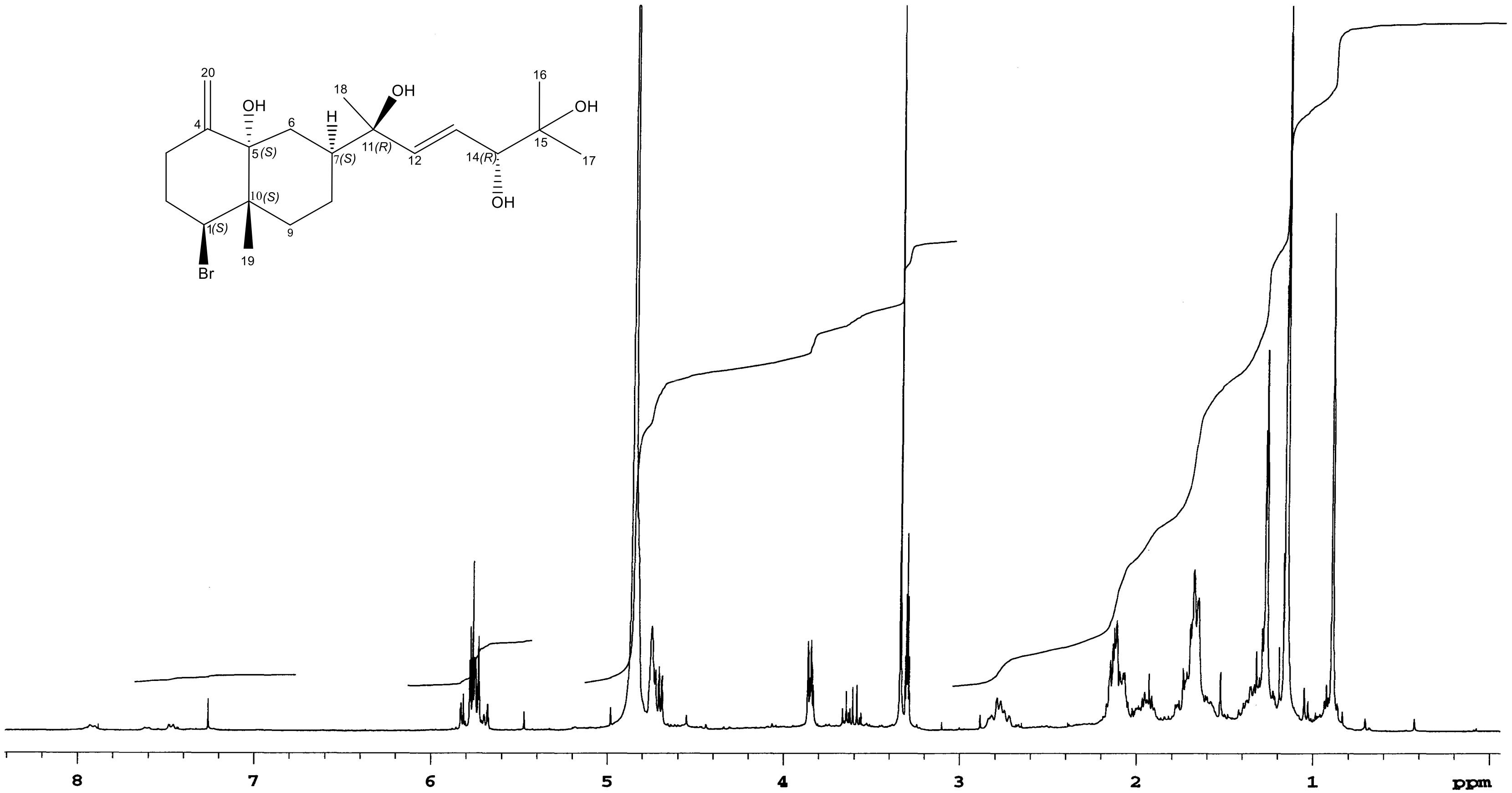

Figure S57: ^{1}H NMR spectrum (300 MHz, CD_3OD) of dihydroaplysia-5,11,14,15-tetrol (**5**)

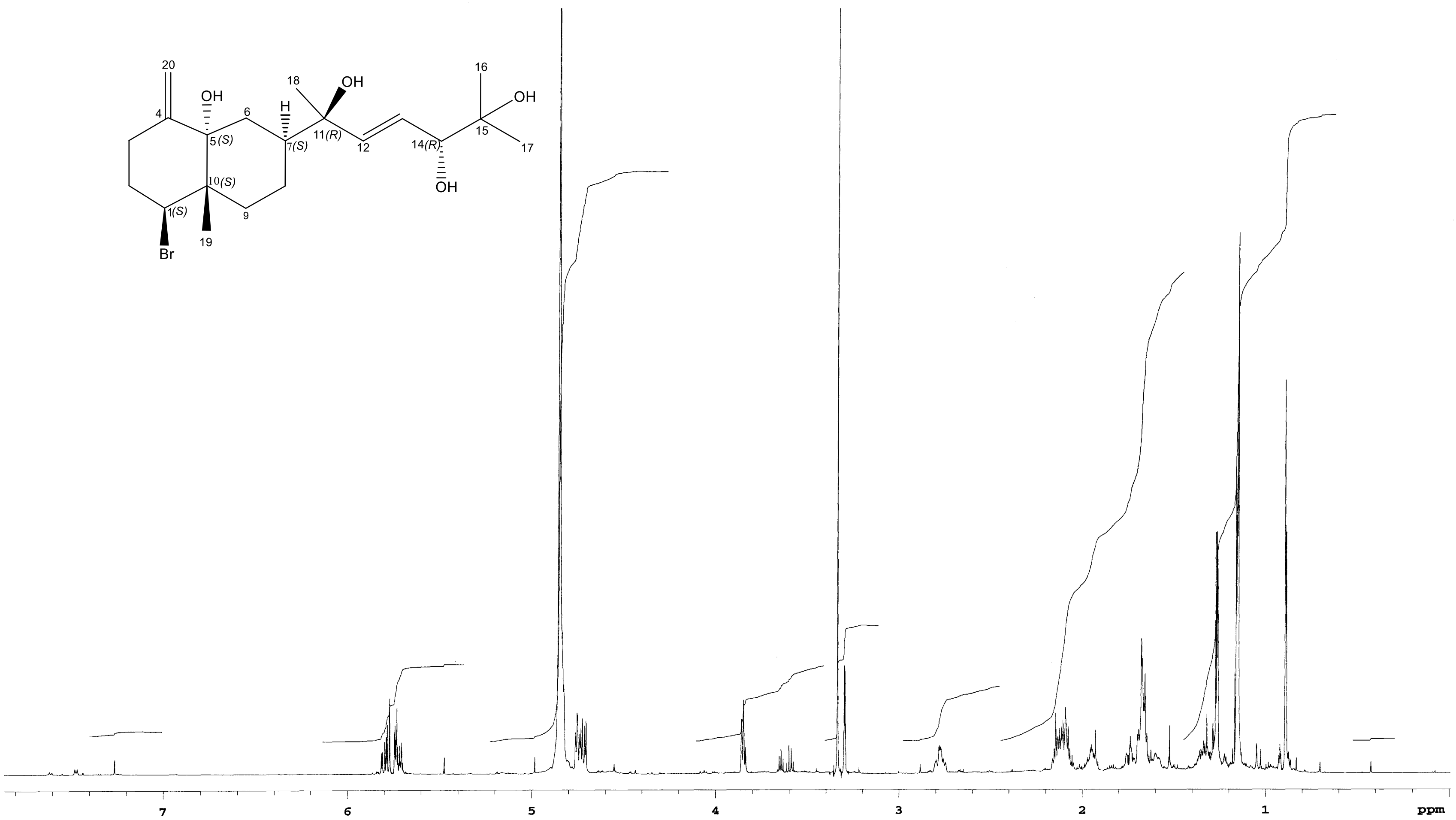

Figure S58: ^{1}H NMR spectrum (600 MHz, CD_3OD) of 10,13-dihydroxy-11-ene-aplysiadiol (**5**)

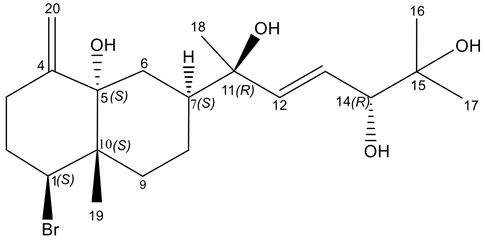

MSGL2 cd3od
Shaaban / Laatsch / CS

SPECTRAL LINES FOR TH= 4.9
FROM -2.5 PPM TO 197.5 PPM
RFL= 7922.1 RFP= 6159.0

INDEX	FREQ	PPM	HEIGHT
1	19061.1	151.65	13.5
2	19056.5	151.61	8.3
3	19053.3	151.58	7.7
4	17655.3	140.46	8.5
5	17576.2	139.83	14.6
6	17572.9	139.81	10.7
7	17546.2	139.60	9.2
8	16100.0	128.09	19.2
9	16083.0	127.95	10.2
10	16071.9	127.87	9.4
11	13723.0	109.18	13.0
12	13720.3	109.16	20.5
13	10105.6	80.40	8.9
14	10098.2	80.34	11.7
15	10087.6	80.26	9.5
16	10082.1	80.21	9.9
17	9713.9	77.28	11.1
18	9709.3	77.25	10.4
19	9707.0	77.23	10.1
20	9476.3	75.39	10.1
21	9470.4	75.34	18.9
22	9263.2	73.70	11.9
23	9259.1	73.66	7.5
24	9256.8	73.65	8.7
25	8198.6	65.23	22.9
26	6265.8	49.85	12.2
27	6223.4	49.51	28.3
28	6201.8	49.34	85.2
29	6180.6	49.17	173.0

INDEX	FREQ	PPM	HEIGHT
29	6180.6	49.17	173.0
30	6159.0	49.00	200.0
31	6137.8	48.83	175.1
32	6116.2	48.66	86.2
33	6095.0	48.49	30.1
34	5552.3	44.17	29.0
35	5493.9	43.71	10.5
36	5487.4	43.66	12.2
37	5465.8	43.48	20.6
38	4476.2	35.61	40.
39	4275.9	34.02	14.
40	4269.5	33.97	14.
41	4235.9	33.70	41.
42	4179.3	33.25	15.
43	4115.3	32.74	14.
44	3307.0	26.31	11.
45	3302.9	26.28	9.
46	3296.4	26.23	10.
47	3292.7	26.20	10.
48	3277.1	26.07	9.
49	3267.0	25.99	16.
50	3162.9	25.16	11.
51	3157.0	25.12	17.
52	3151.9	25.08	11.
53	2890.4	23.00	11.
54	2883.1	22.94	10.
55	2800.2	22.28	9.
56	2793.8	22.23	9.
57	1912.8	15.22	29.

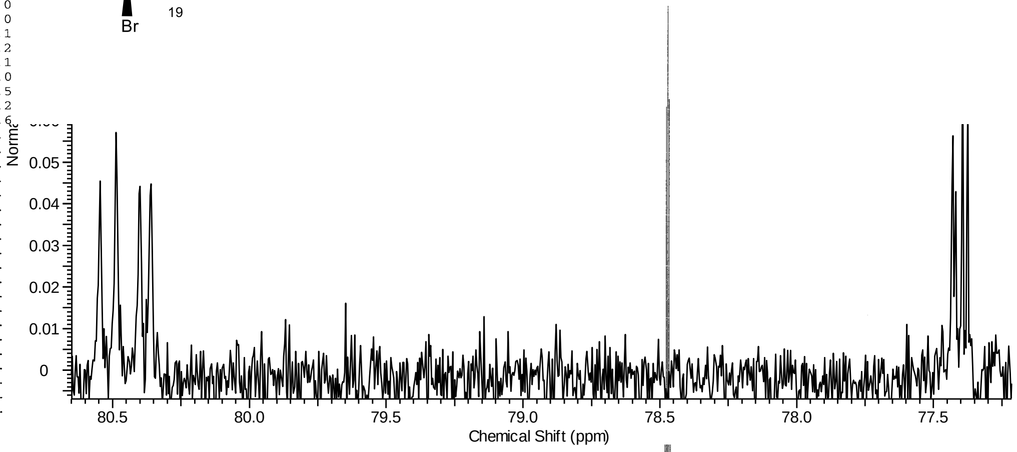

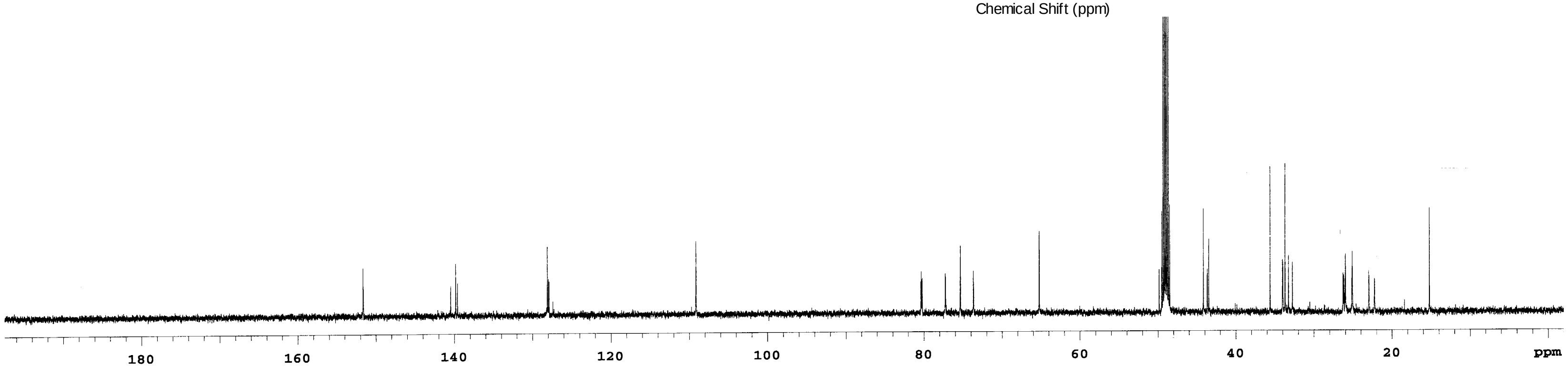

Figure S59: ^{13}C NMR spectrum (125 MHz, CD_3OD) of dihydroaplysia-5,11,14,15-tetrol (**5**) with magnified section of the two signals between 77-81 ppm.

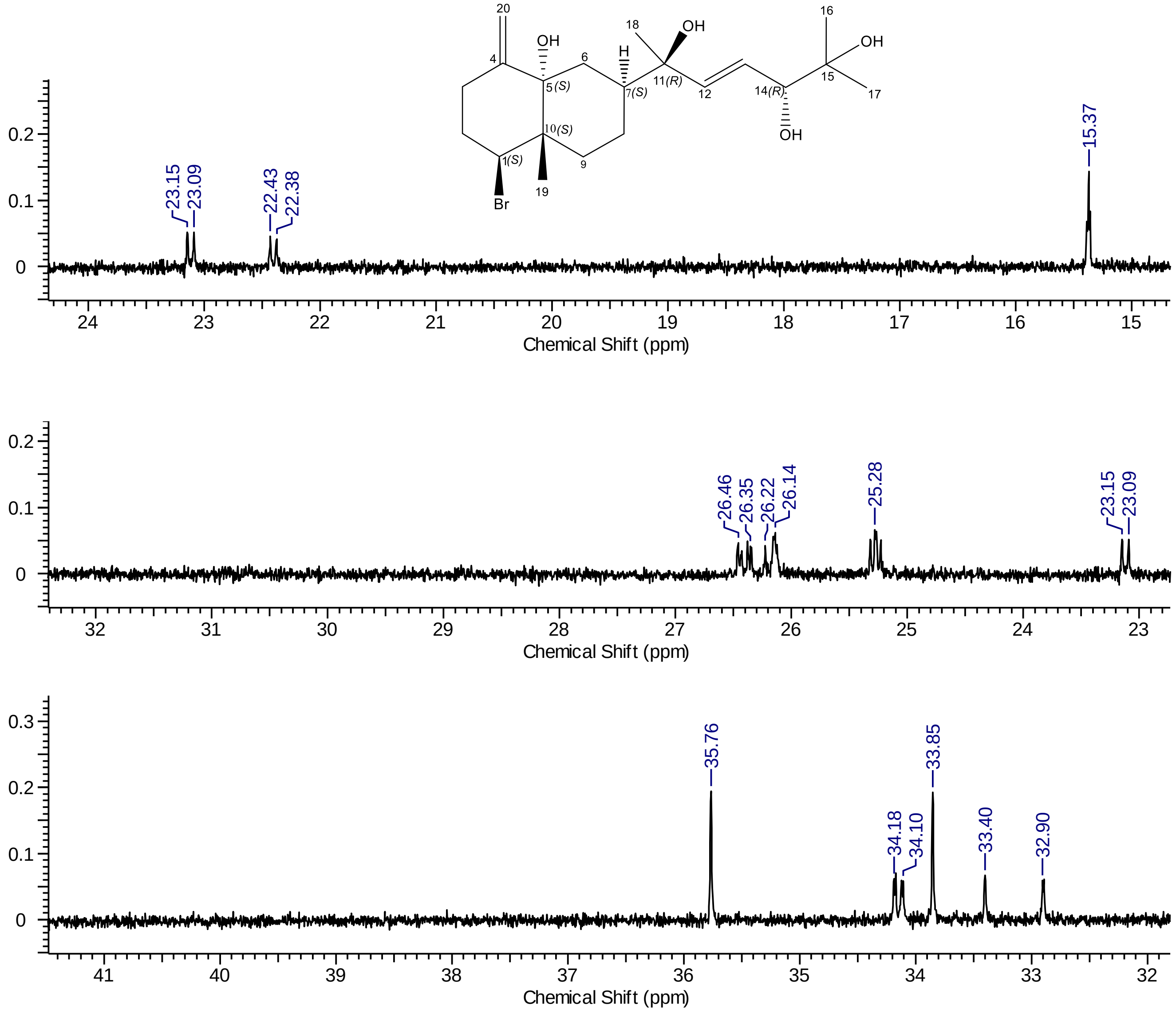

Figure S60: Magnified ^{13}C NMR spectrum (125 MHz, CD_3OD) of dihydroaplysia-5,11,14,15-tetrol (**5**)

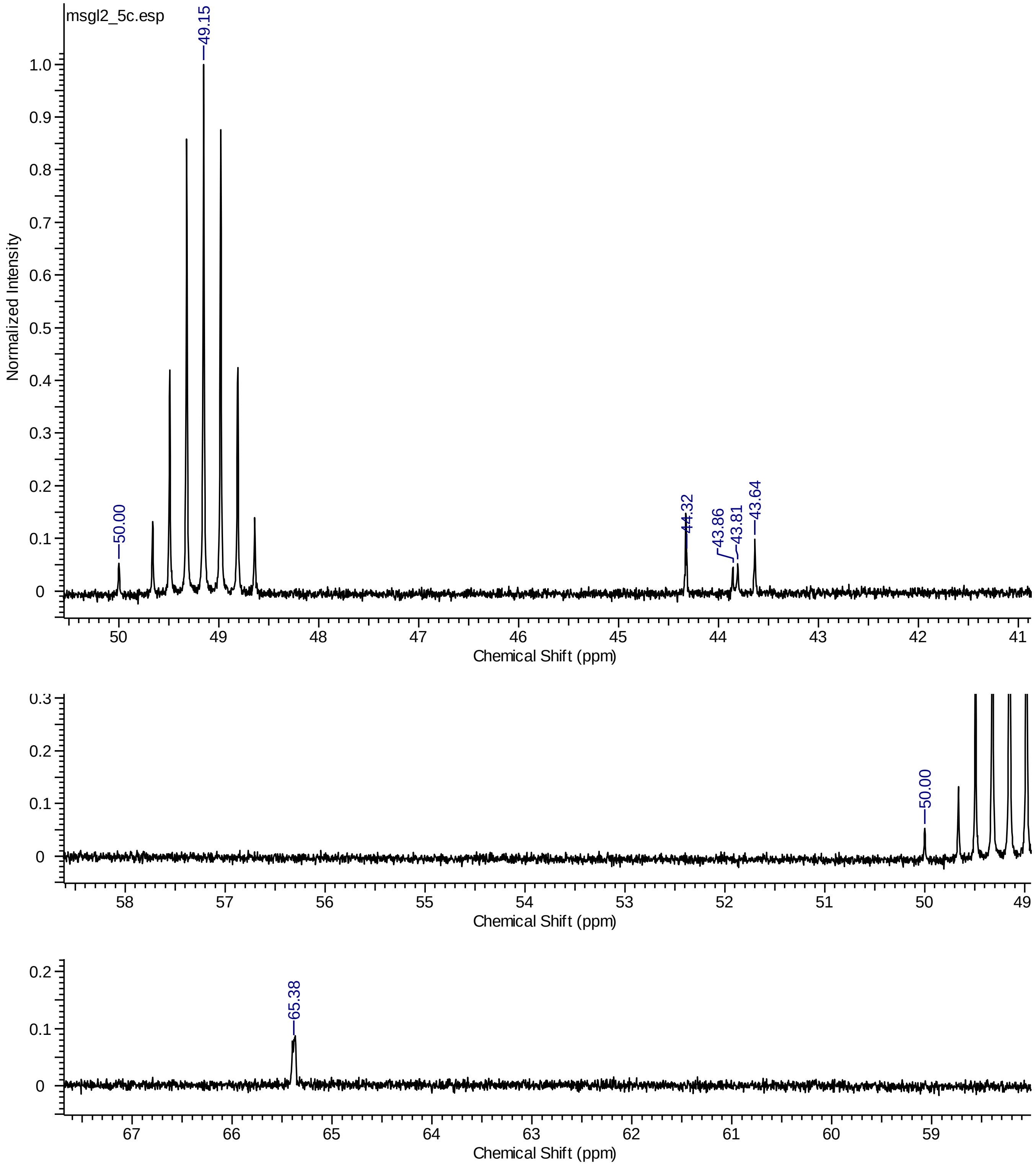

Figure S58 continued: Magnified ^{13}C NMR spectrum (125 MHz, CD_3OD) of dihydroaplysia-5,11,14,15-tetrol (**5**)

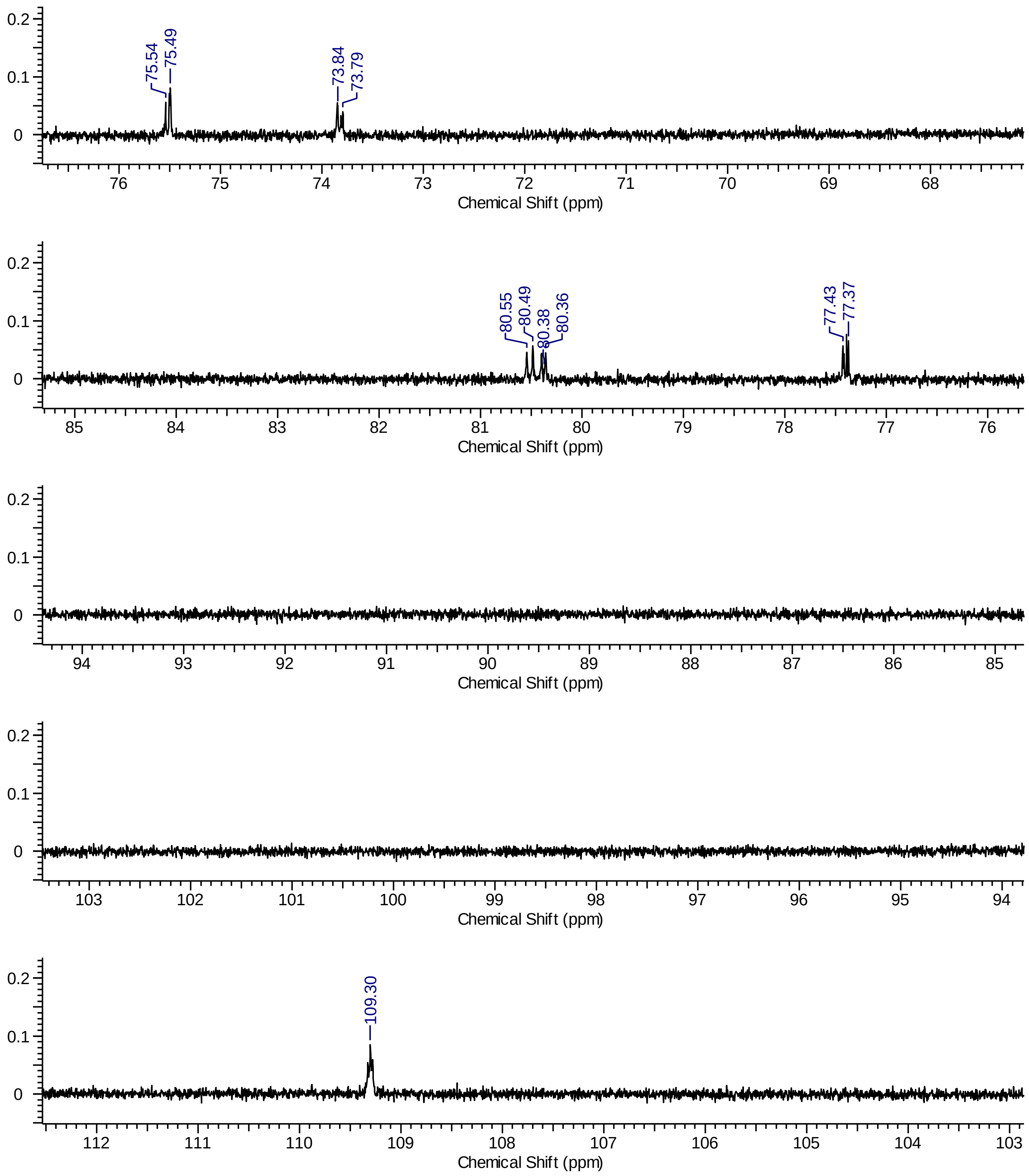

Figure S58 continued: Magnified ^{13}C NMR spectrum (125 MHz, CD_3OD) of dihydroaplysia-5,11,14,15-tetrol (**5**)

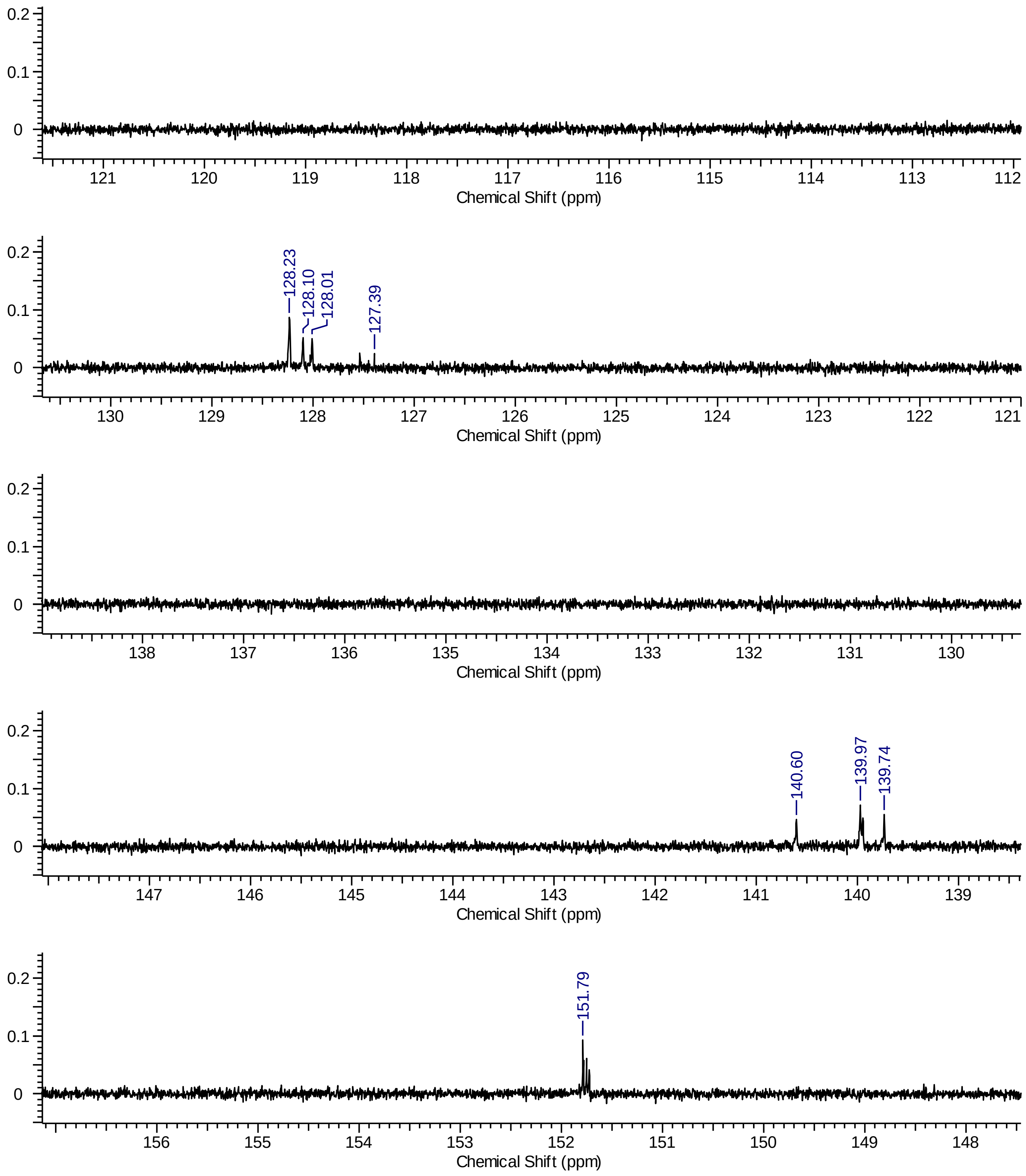

Figure S58 continued: Magnified ^{13}C NMR spectrum (125 MHz, CD_3OD) of dihydroaplysia-5,11,14,15-tetrol (**5**)

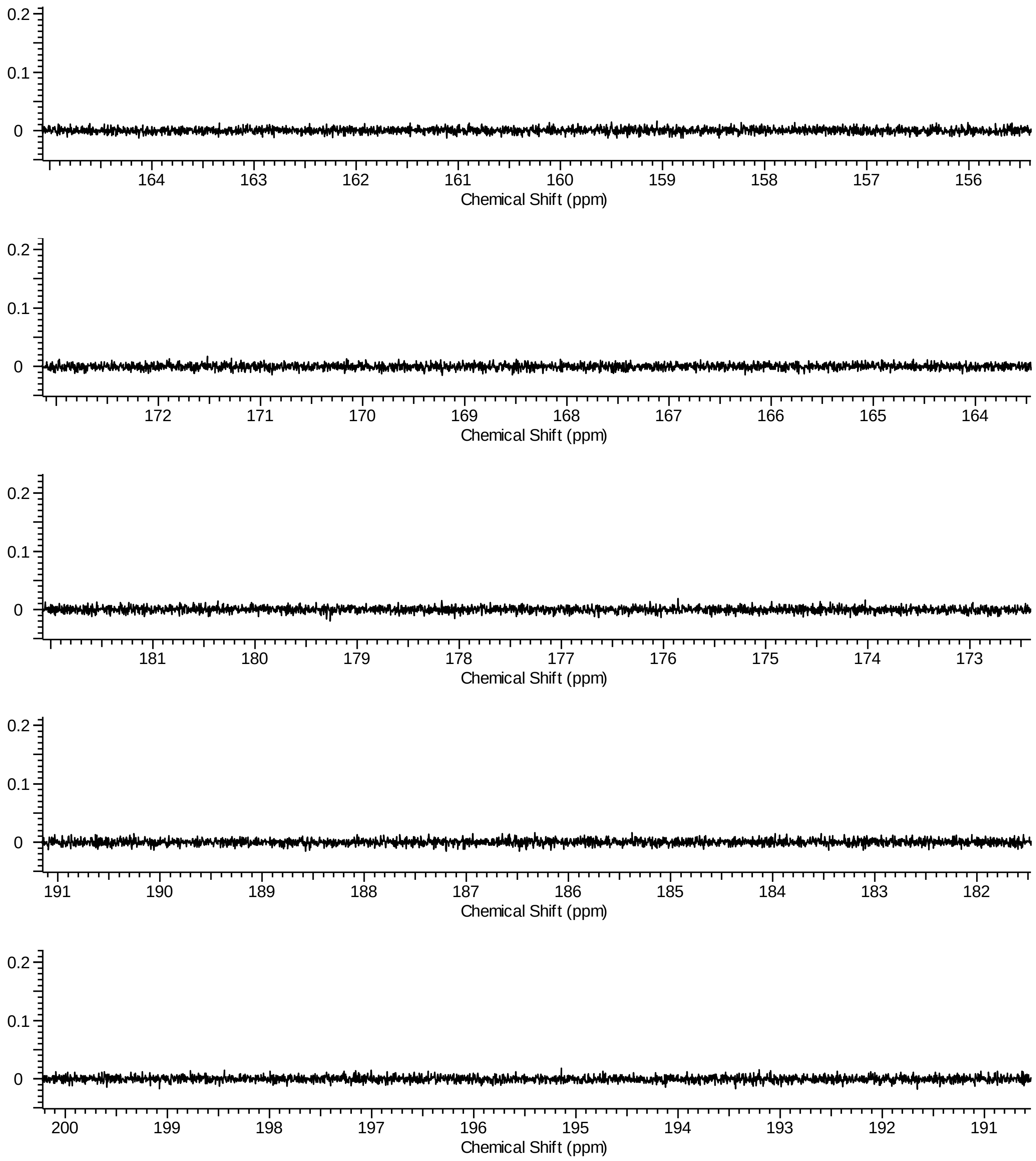

Figure S58 continued: Magnified ^{13}C NMR spectrum (125 MHz, CD_3OD) of dihydroaplysia-5,11,14,15-tetrol (**5**)

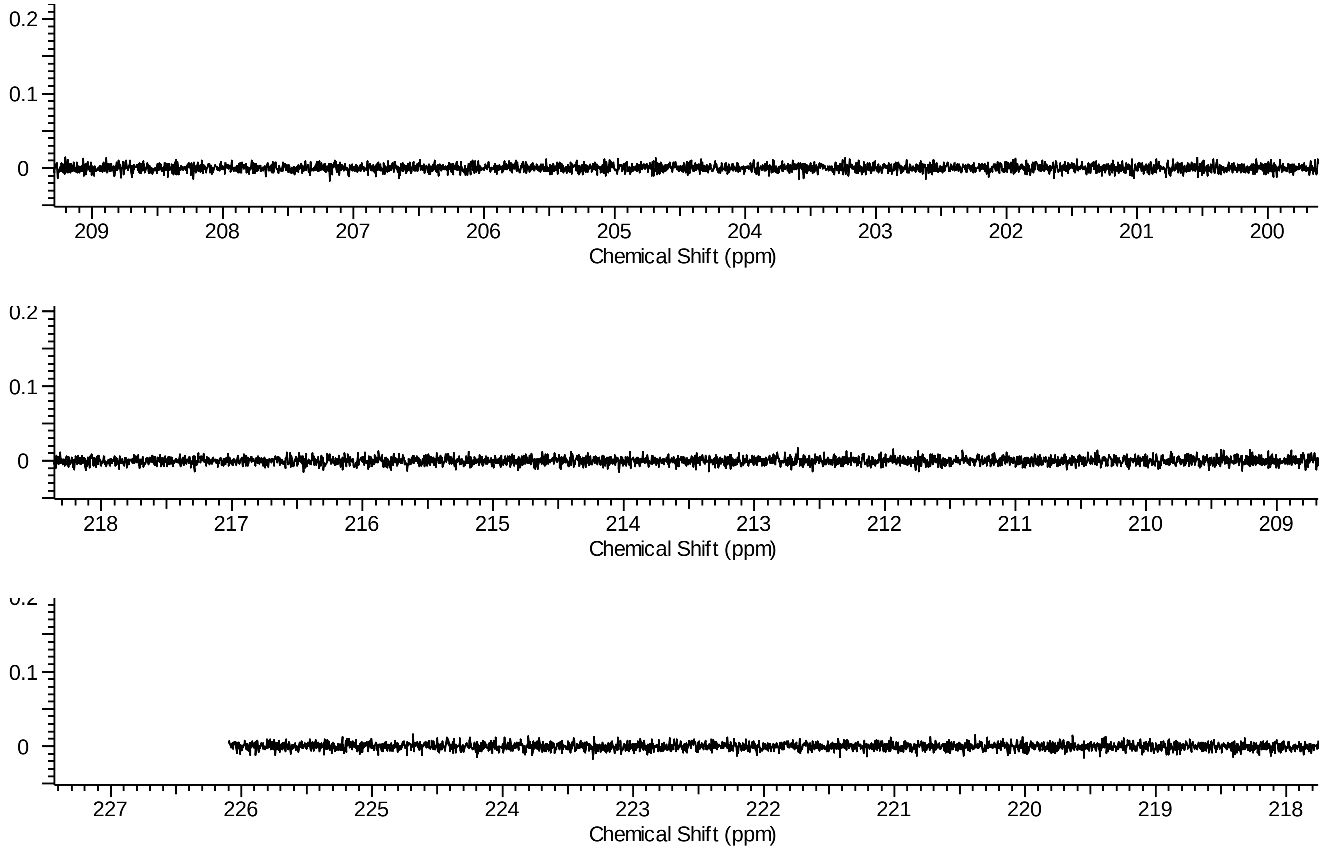

Figure S58 continued: Magnified ^{13}C NMR spectrum (125 MHz, CD_3OD) of dihydroaplysia-5,11,14,15-tetrol (**5**)

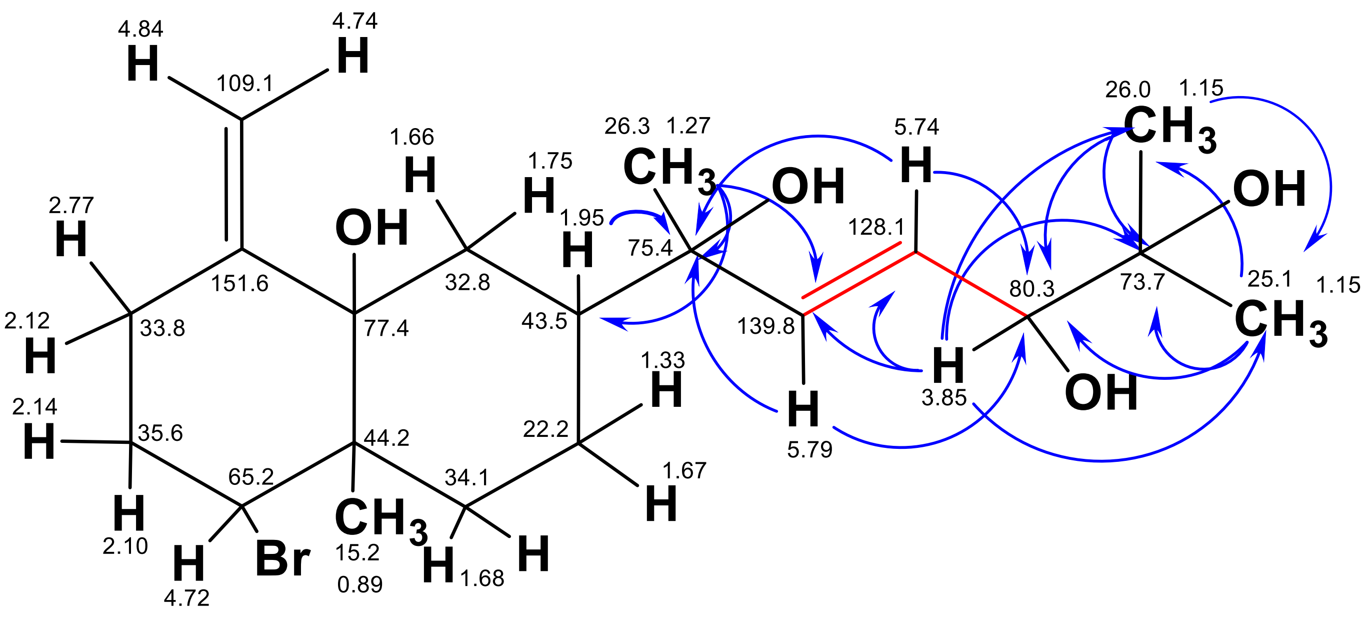

Figure S61: H,H COSY (▬) and HMBC (⌒) connectivities of 11,14-dihydroaplysia-5,11,14,15-tetrols (**5**); correlations in the decalin ring were the same as in **1-3**.

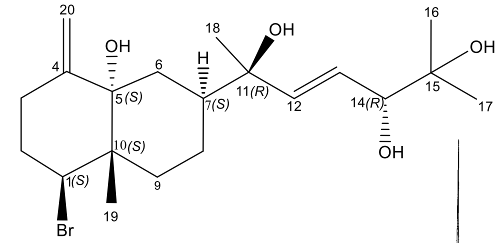

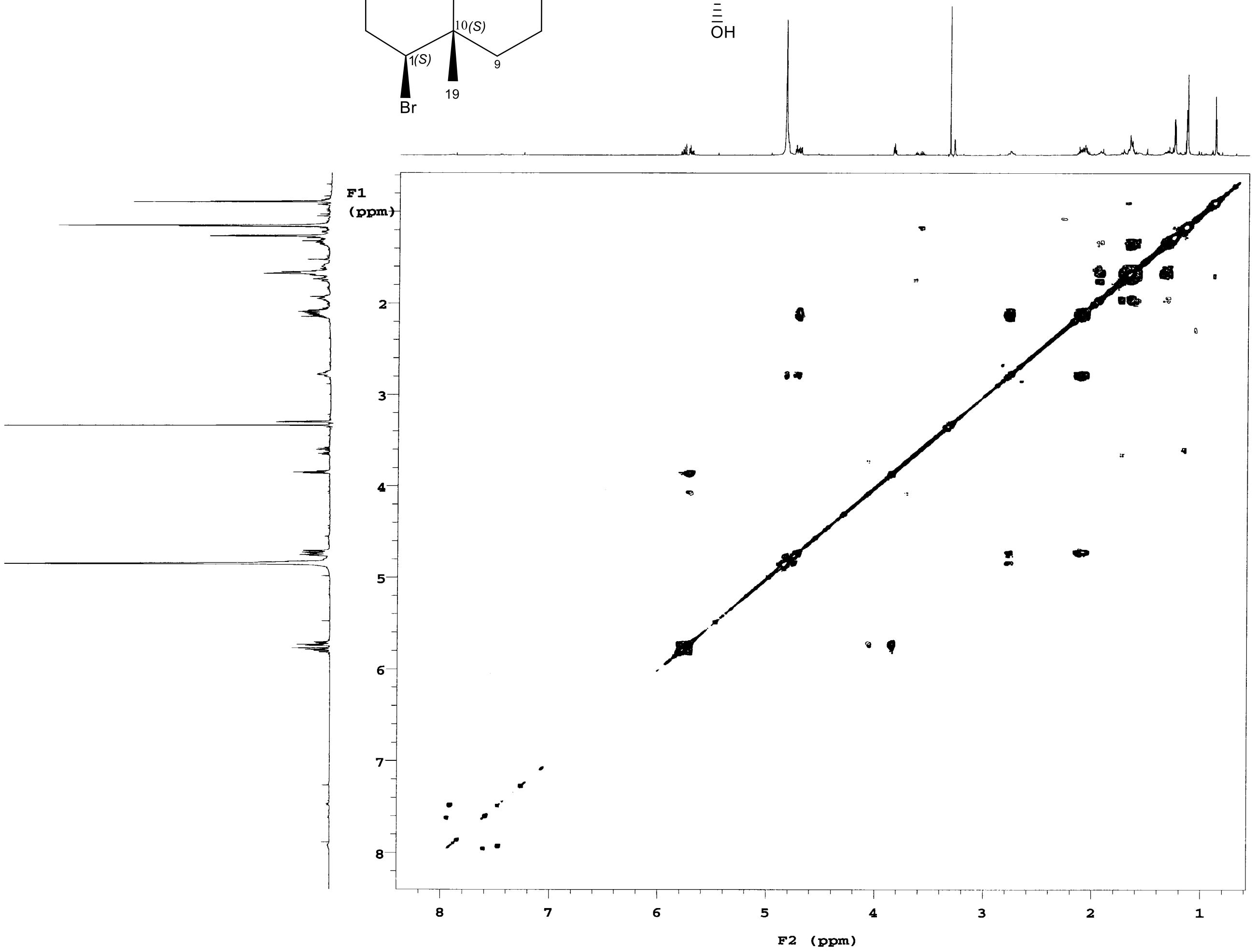

Figure S62: H,H COSY spectrum (500 MHz, CD_3OD) of dihydroaplysia-5,11,14,15-tetrol (**5**)

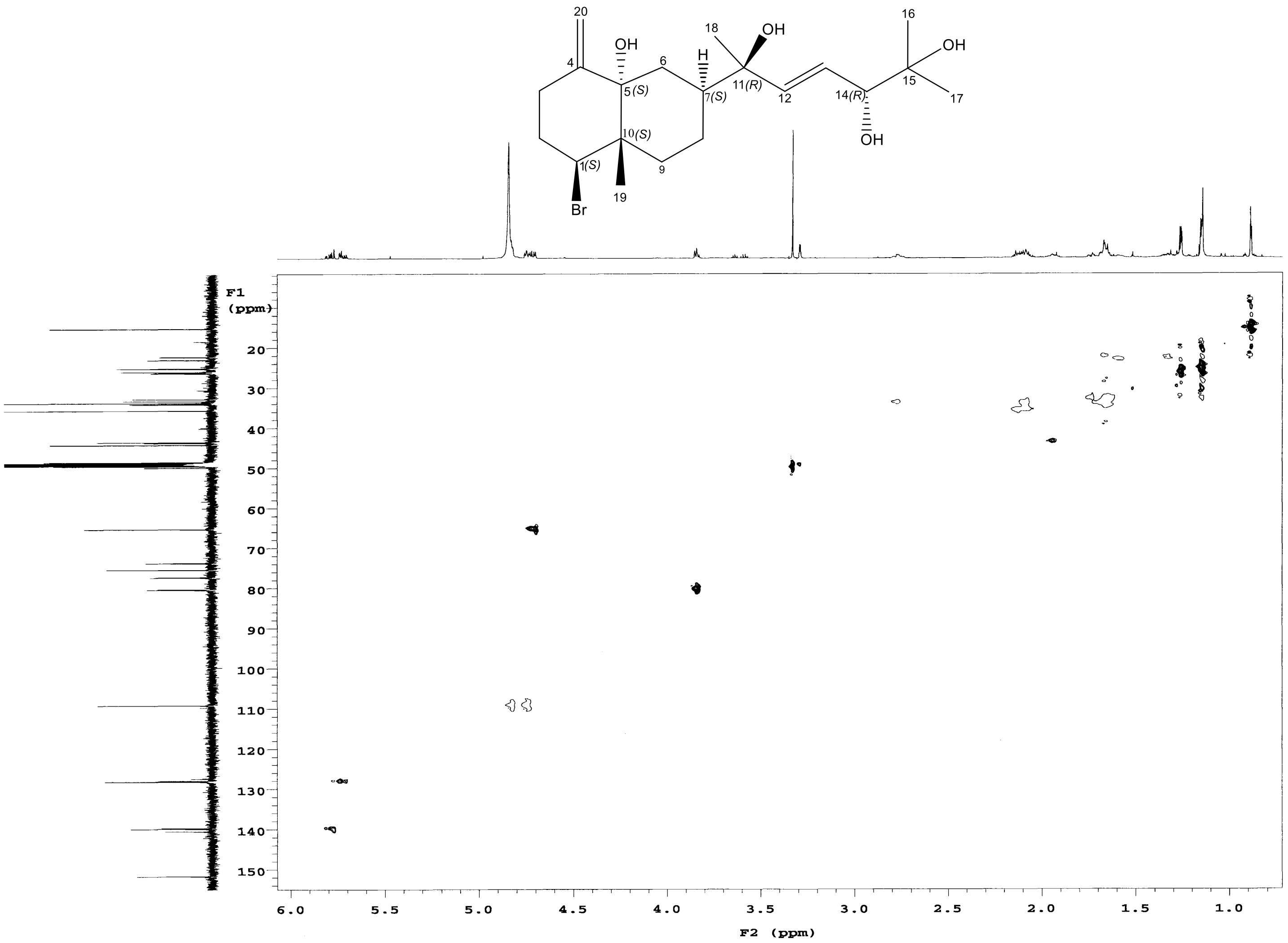

Figure S63: HMQC spectrum (500 MHz, CD_3OD) of dihydroaplysia-5,11,14,15-tetrol (**5**)

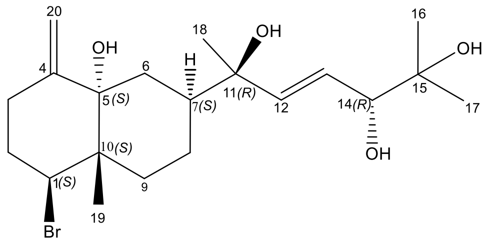

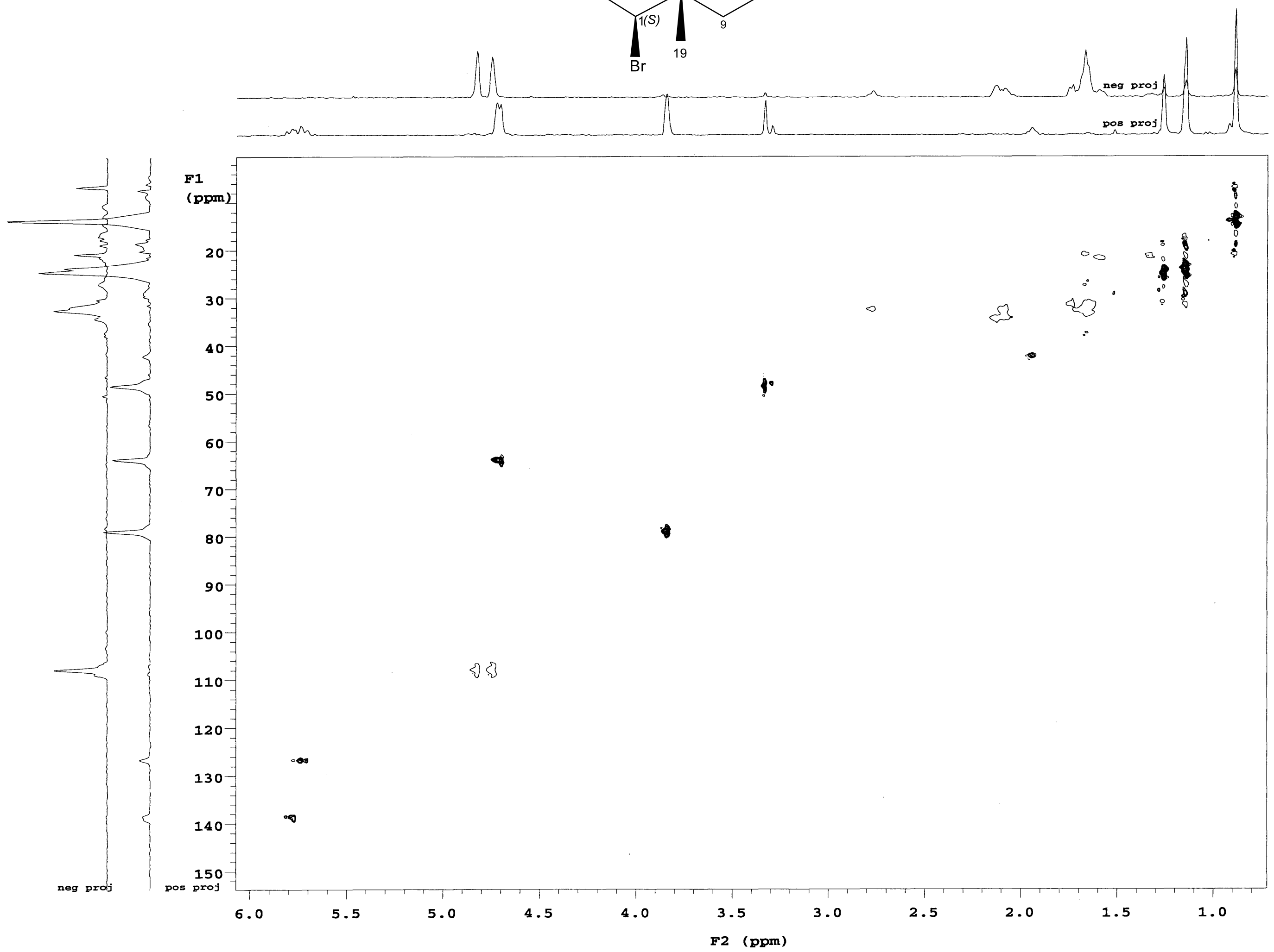

Figure S64: HSQC spectrum (500 MHz, CD_3OD) of dihydroaplysia-5,11,14,15-tetrol (**5**)

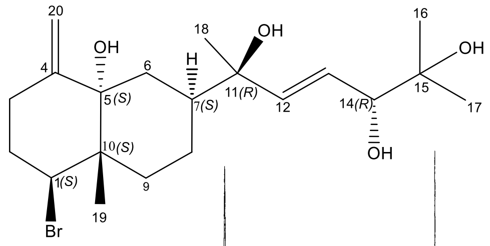

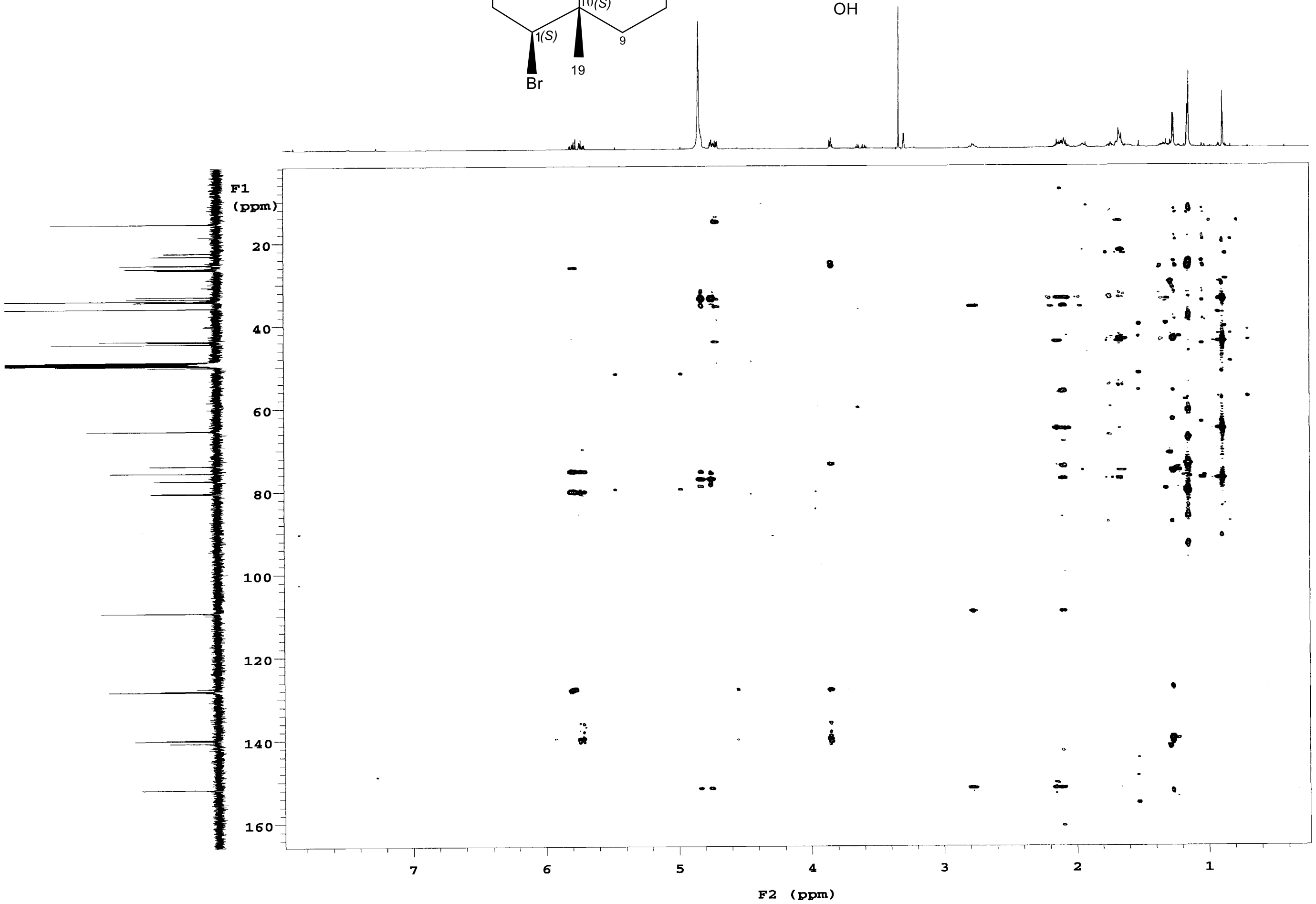

Figure S65: HMBC spectrum (500 MHz, CD_3OD) of dihydroaplysia-5,11,14,15-tetrol (**5**)

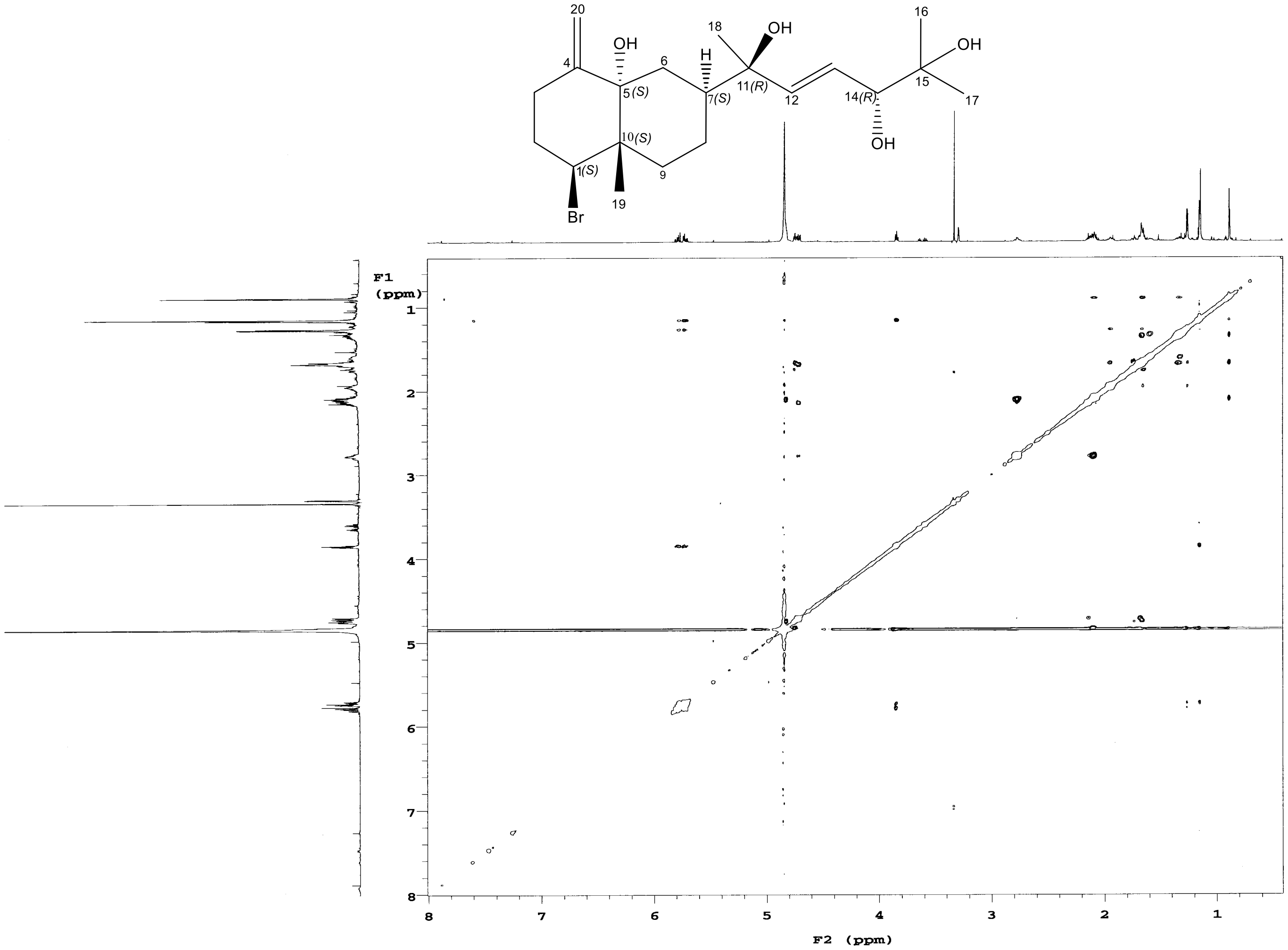

Figure S66: NOESY spectrum (500 MHz, CD_3OD) of dihydroaplysia-5,11,14,15-tetrol (**5**)

Display Report

**Analysis Info**		Acquisition Date	12.02.2016 10:29:10
Analysis Name	Z:\Data\2016\1602\sam120216\mshaaba00045 low_1_01_86174.d		
Method	hystar_pl.m	Operator	BDAL@DE
Sample Name	mshaaba00045 low	Instrument / Ser#	micrOTOF 10237
Comment			

**Acquisition Parameter**					
Source Type	ESI	Ion Polarity	Positive	Set Nebulizer	1.2 Bar
Focus	Not active			Set Dry Heater	180 °C
Scan Begin	50 m/z	Set Capillary	4500 V	Set Dry Gas	6.0 l/min
Scan End	1600 m/z	Set End Plate Offset	-500 V	Set Divert Valve	Source

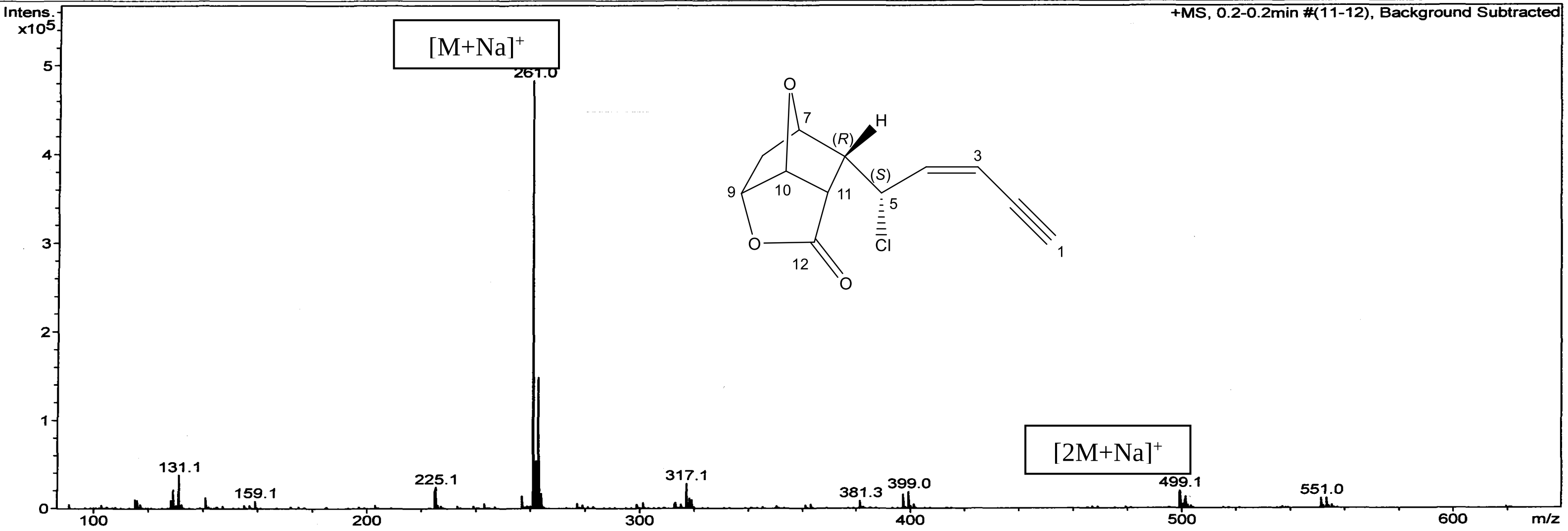

Bruker Compass DataAnalysis 4.0 printed: 12.02.2016 11:04:57 Page 1 of 1

Figure S67: (+)-ESI mass spectrum of 5-*epi*-maneolactone (**6**)

Mass Spectrum SmartFormula Report

Analysis Info

Analysis Name	Z:\Data\2016\1602\sam120216\mshaaba00045 low_1_01_86174.d	Acquisition Date	12.02.2016 10:29:10
Method	hystar_pl.m	Operator	BDAL@DE
Sample Name	mshaaba00045 low	Instrument / Ser#	micrOTOF 10237
Comment			

Acquisition Parameter

Source Type	ESI	Ion Polarity	Positive	Set Nebulizer	1.2 Bar
Focus	Not active			Set Dry Heater	180 °C
Scan Begin	50 m/z	Set Capillary	4500 V	Set Dry Gas	6.0 l/min
Scan End	1600 m/z	Set End Plate Offset	-500 V	Set Divert Valve	Source

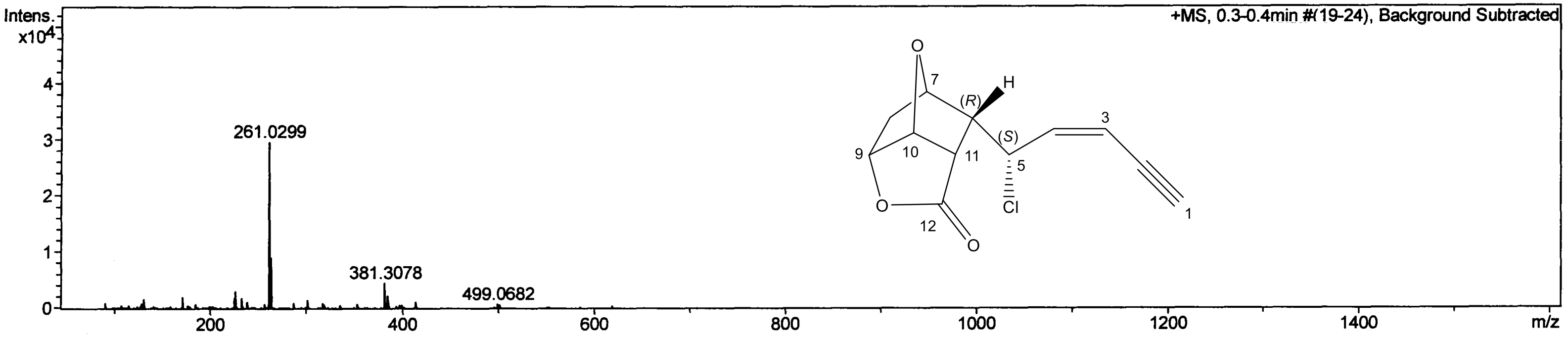

Meas. m/z	#	Formula	m/z	err [ppm]	Mean err [ppm]	rdb	N-Rule	e⁻ Conf	mSigma	Std I	Std Mean m/z	Std I VarNorm	Std m/z Diff	Std Comb Dev
261.0299														
	1	C 8 H 7 Cl N 6 Na O	261.0262	-14.2	-13.6	7.5	ok	even	8.69	0.0120	0.0036	0.0056	0.0007	0.9656
	2	C 12 H 11 Cl Na O 3	261.0289	-3.9	-3.2	6.5	ok	even	13.72	0.0216	0.0009	0.0077	0.0007	0.8761

Figure S68: (+)-ESI HR mass spectrum of 5-*epi*-maneolactone (**6**)

Display Report

Analysis Info

Analysis Name	Z:\Data\2016\1602\sam120216\mshaaba00045 low_1_01_86174.d	Acquisition Date	12.02.2016 10:29:10
Method	hystar_pl.m	Operator	BDAL@DE
Sample Name	mshaaba00045 low	Instrument / Ser#	micrOTOF 10237
Comment			

Acquisition Parameter

Source Type	ESI	Ion Polarity	Positive	Set Nebulizer	1.2 Bar
Focus	Not active			Set Dry Heater	180 °C
Scan Begin	50 m/z	Set Capillary	4500 V	Set Dry Gas	6.0 l/min
Scan End	1600 m/z	Set End Plate Offset	-500 V	Set Divert Valve	Source

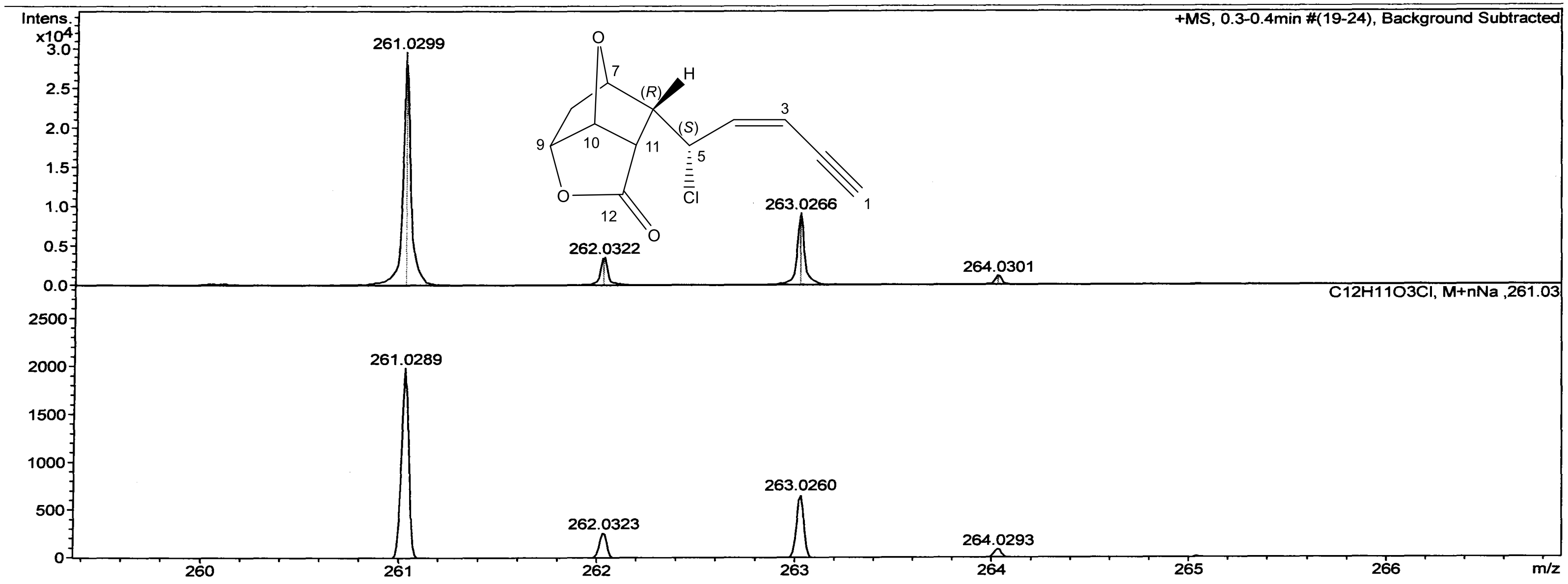

Bruker Compass DataAnalysis 4.0 printed: 15.02.2016 15:04:32 Page 1 of 1

Figure S69: (+)-ESI HR mass spectrum of 5-*epi*-maneolactone (**6**)

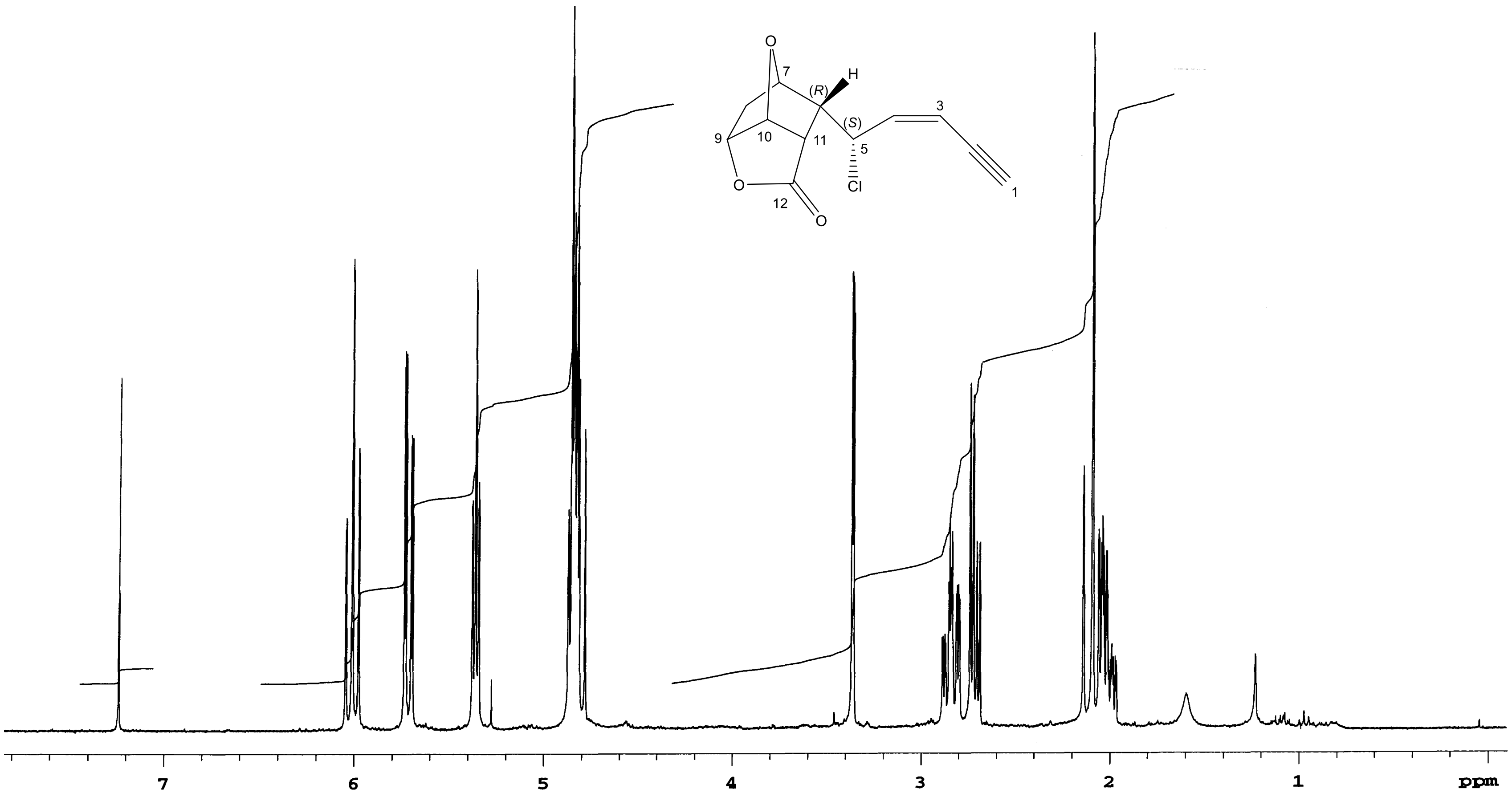

Figure S70: ^{1}H NMR spectrum (300 MHz, $CDCl_3$) of 5-*epi*-maneolactone (**6**)

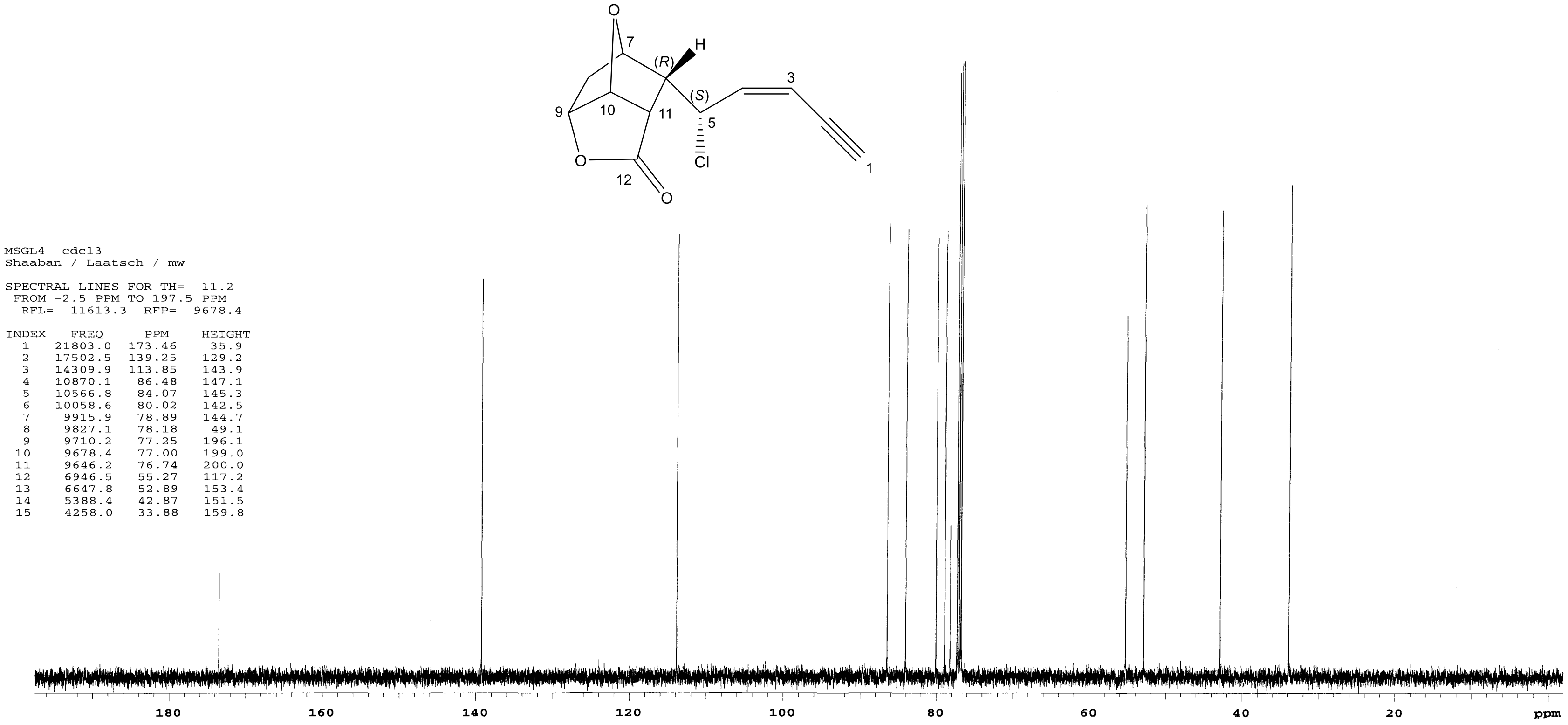

Figure S71: ^{13}C NMR spectrum (125 MHz, $CDCl_3$) of 5-*epi*-maneolactone (**6**)

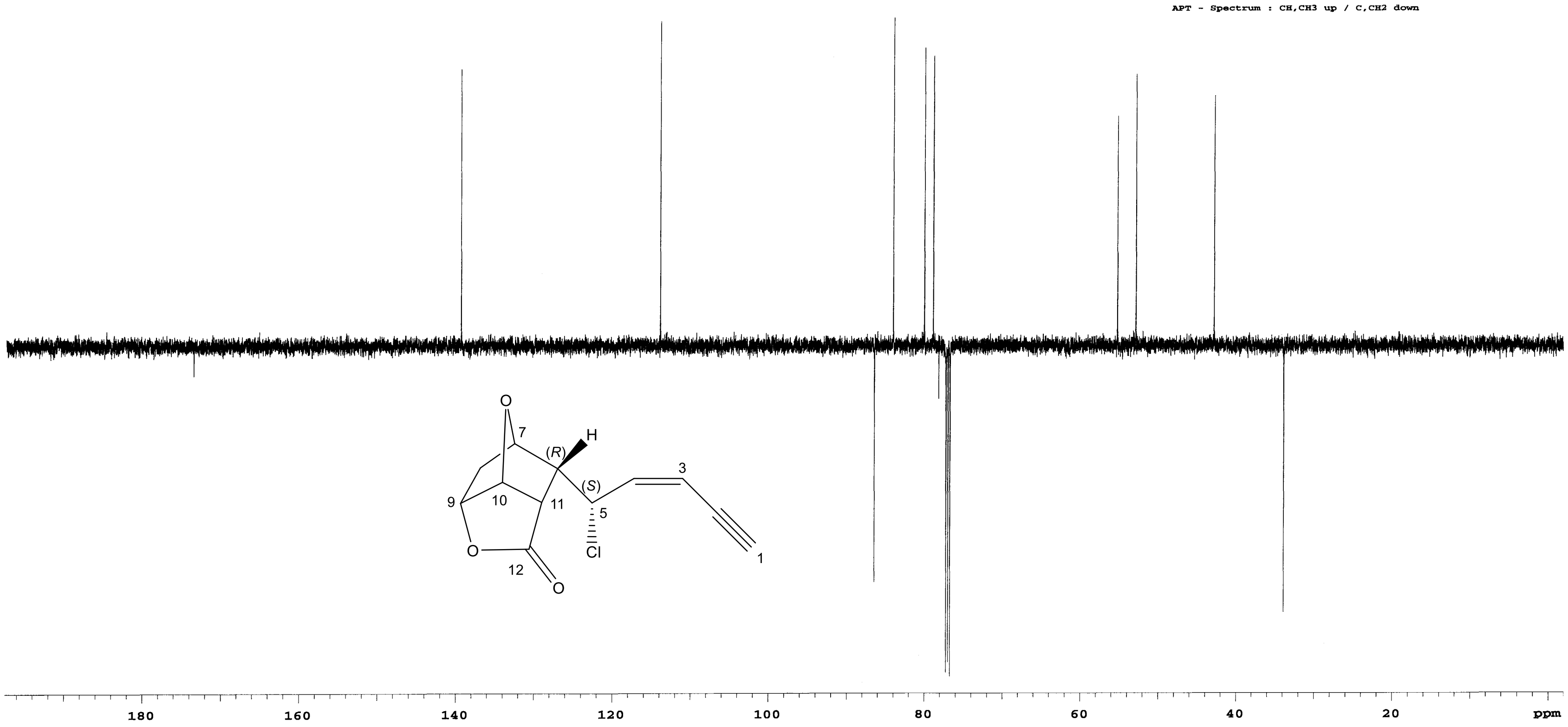

Figure S72: APT NMR spectrum (125 MHz, $CDCl_3$) of 5-*epi*-maneolactone (**6**)

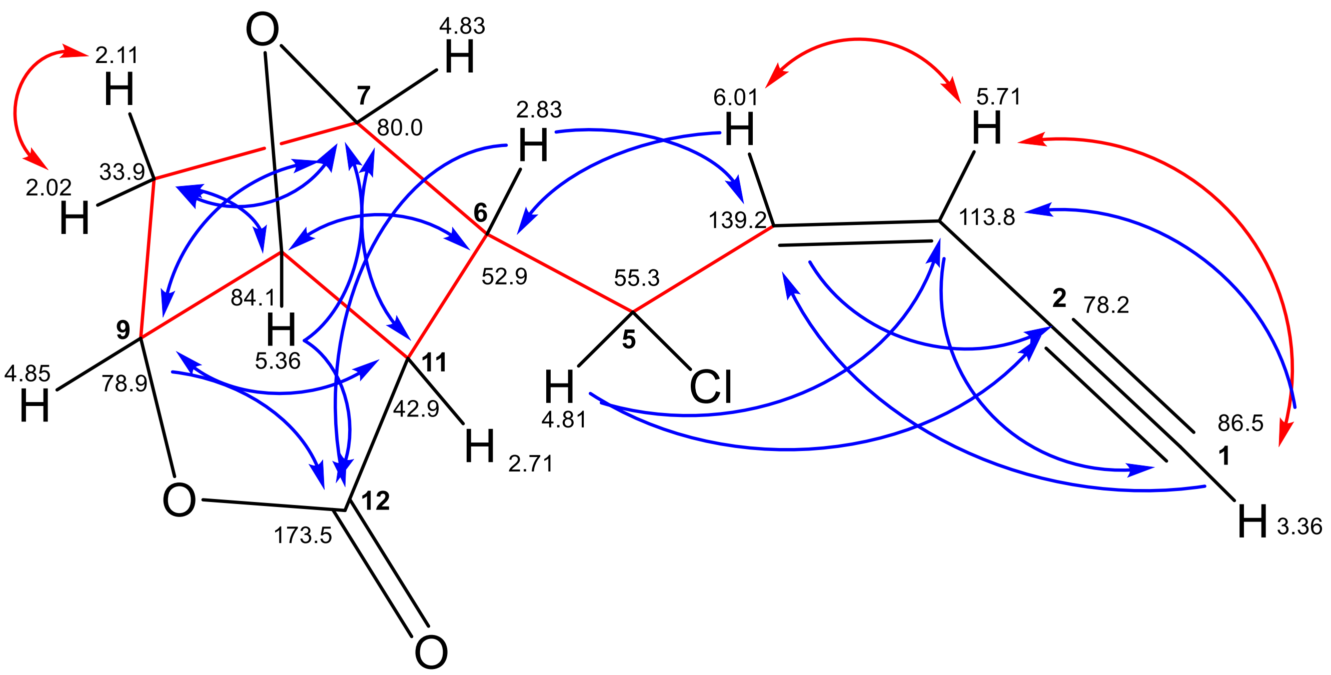

Figure S73: H,H COSY (———) and selected HMBC ( ) correlations of 5-*epi*-maneolactone (**6**).

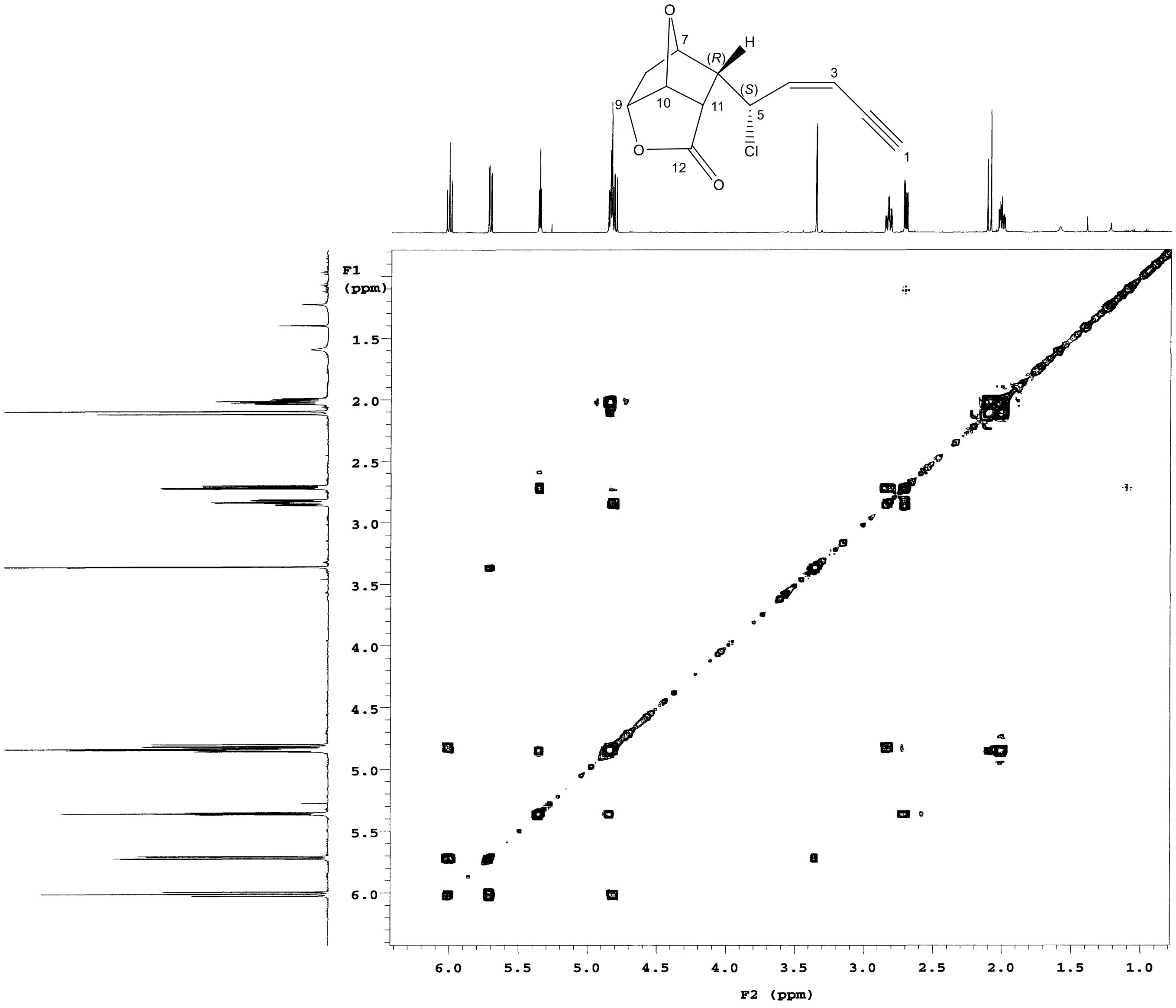

Figure S74: H,H COSY spectrum (500 MHz, $CDCl_3$) of 5-*epi*-maneolactone (**6**)

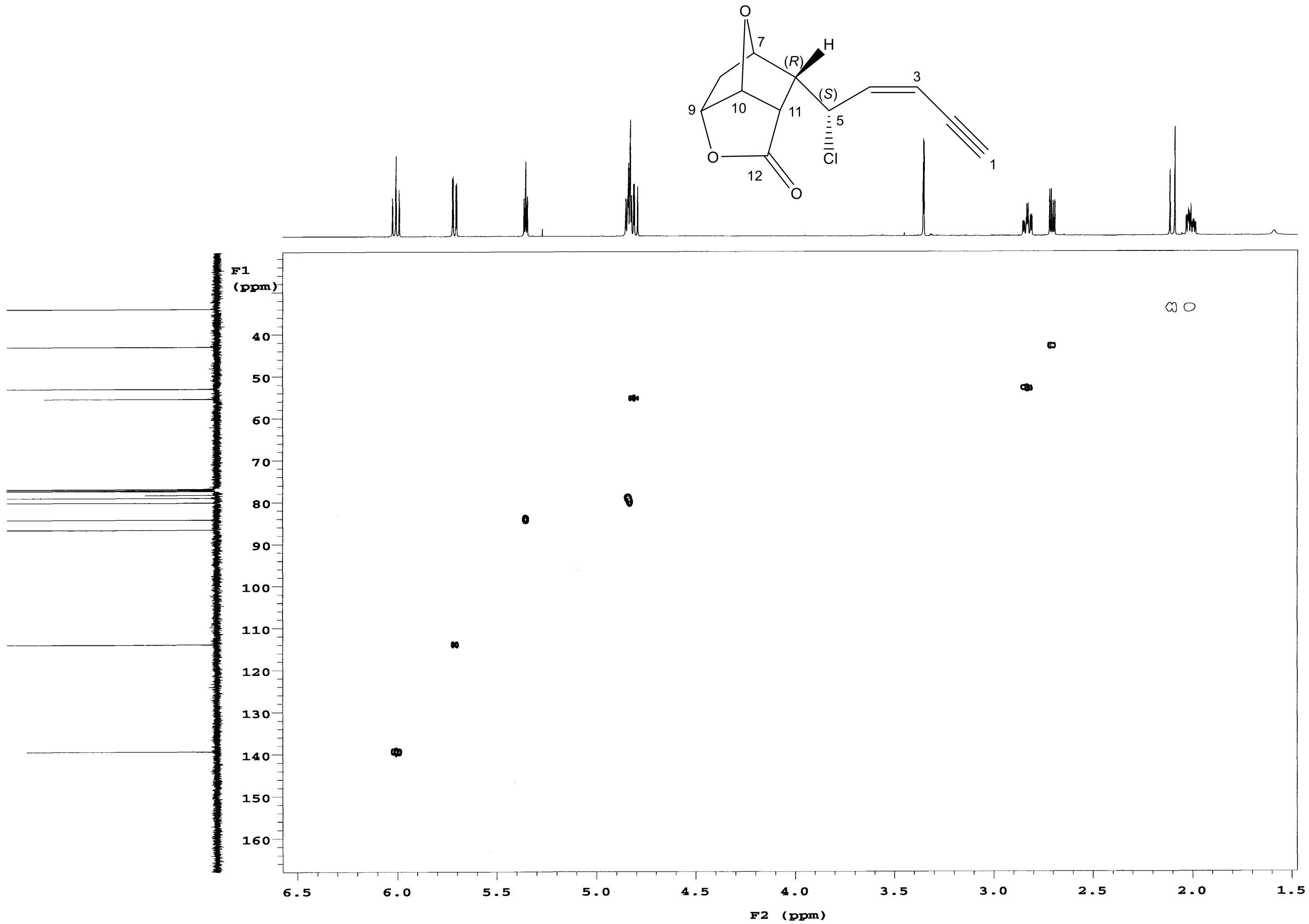

Figure S75: HMQC spectrum (500 MHz, $CDCl_3$) of 5-*epi*-maneolactone (**6**)

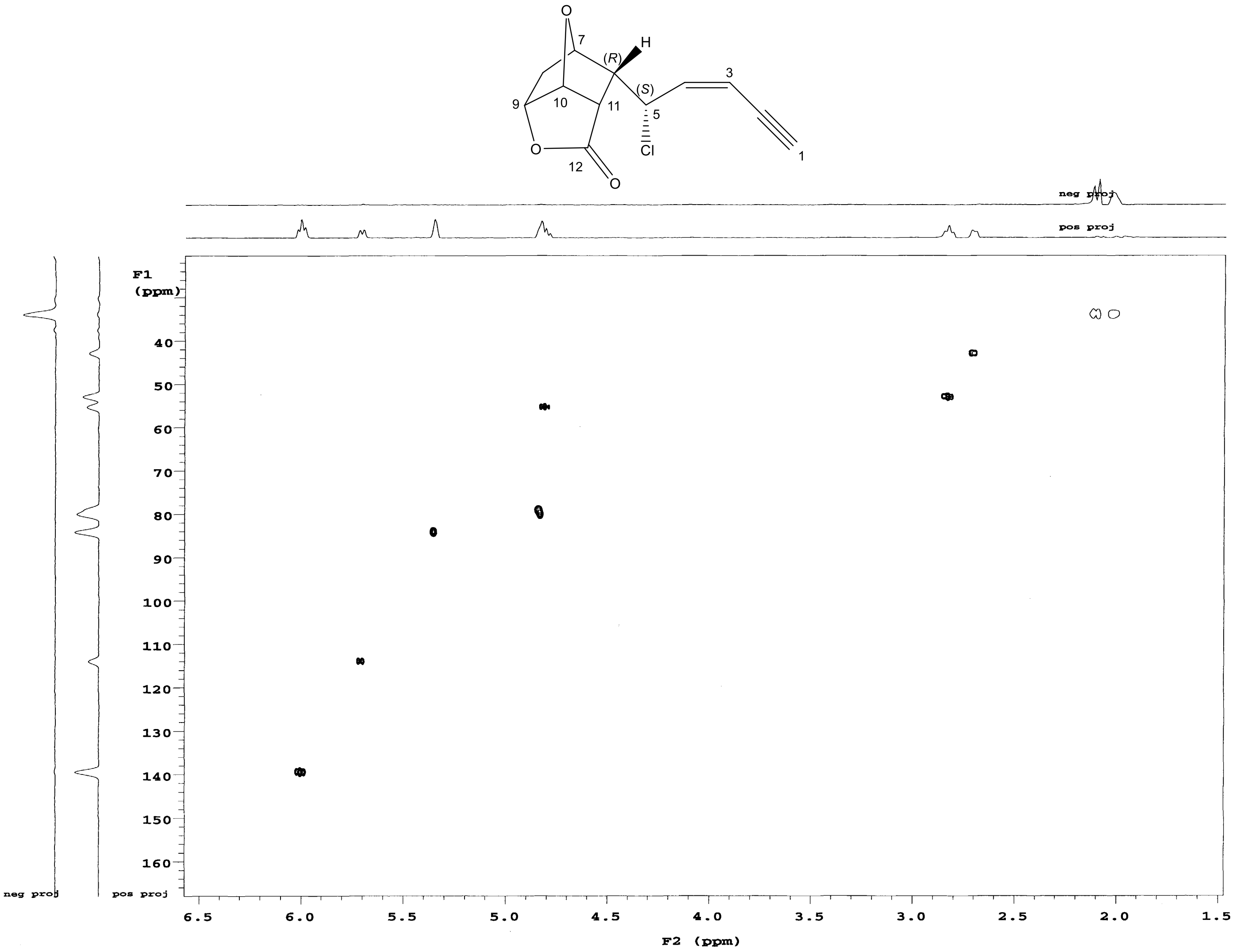

Figure S76: HSQC spectrum (500 MHz, $CDCl_3$) of 5-*epi*-maneolactone (**6**)

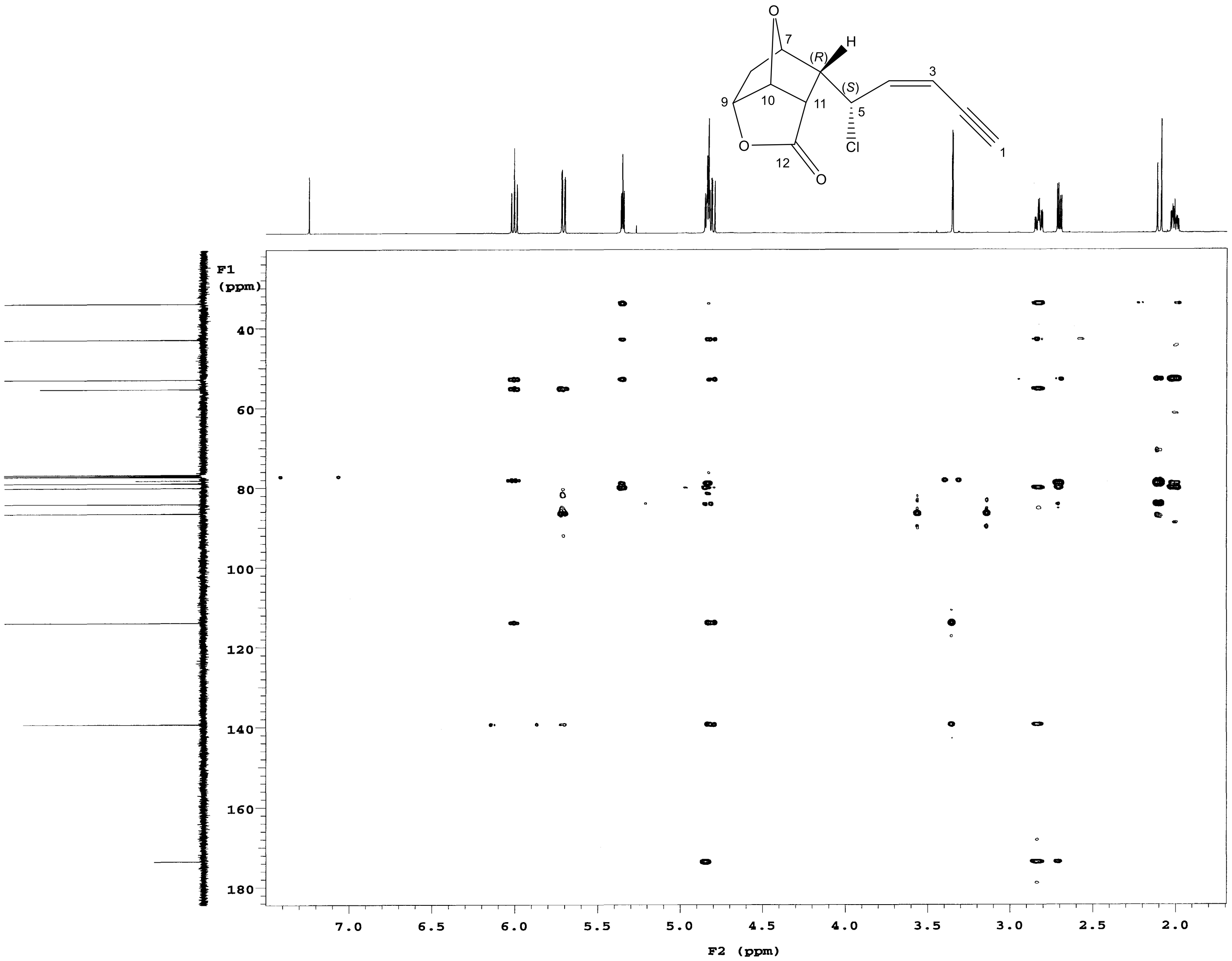

Figure S77: HMBC spectrum (500 MHz, $CDCl_3$) of 5-*epi*-maneolactone (**6**)

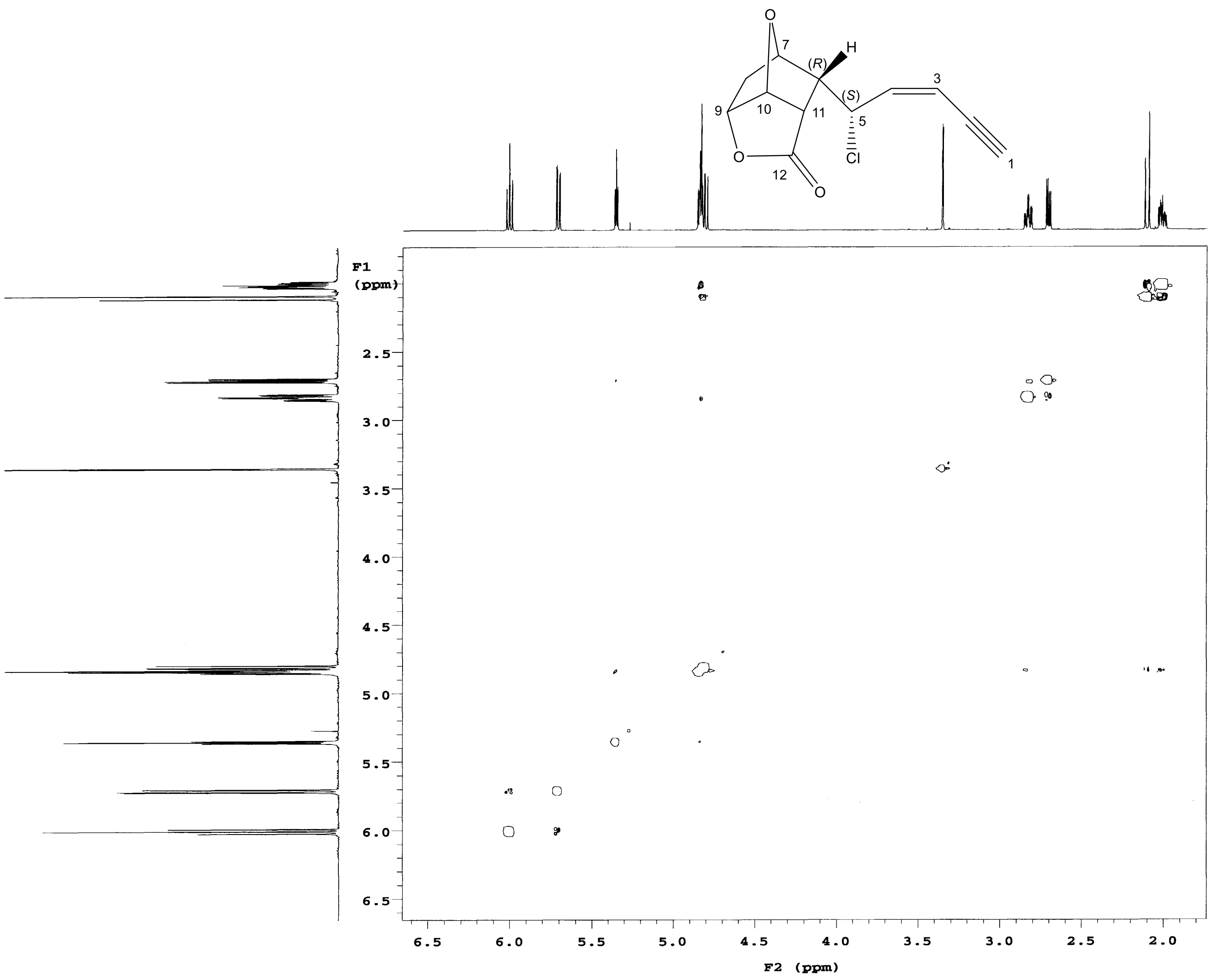

Figure S78: NOESY spectrum (500 MHz, $CDCl_3$) of 5-*epi*-maneolactone (**6**)
